# A Phase I Double Blind, Placebo-Controlled, Randomized Study of the Safety and Immunogenicity of an Adjuvanted HIV-1 Gag-Pol-Nef Fusion Protein and Adenovirus 35 Gag-RT-Int-Nef Vaccine in Healthy HIV-Uninfected African Adults

**DOI:** 10.1371/journal.pone.0125954

**Published:** 2015-05-11

**Authors:** Gloria Omosa-Manyonyi, Juliet Mpendo, Eugene Ruzagira, William Kilembe, Elwyn Chomba, François Roman, Patricia Bourguignon, Marguerite Koutsoukos, Alix Collard, Gerald Voss, Dagna Laufer, Gwynn Stevens, Peter Hayes, Lorna Clark, Emmanuel Cormier, Len Dally, Burc Barin, Jim Ackland, Kristen Syvertsen, Devika Zachariah, Kamaal Anas, Eddy Sayeed, Angela Lombardo, Jill Gilmour, Josephine Cox, Patricia Fast, Frances Priddy

**Affiliations:** 1 Kenya AIDS Vaccine Initiative, University of Nairobi, Nairobi, Kenya; 2 Uganda Virus Research Institute-IAVI, Entebbe, Uganda; 3 Medical Research Council (MRC)/Uganda Virus Research Institute (UVRI), Uganda, Research Unit on AIDS, Entebbe, Uganda; 4 Zambia Emory HIV Research Program, Lusaka, Zambia; 5 University Teaching Hospital, Lusaka, Zambia; 6 GlaxoSmithKline Vaccines, Rixensart, Belgium; 7 International AIDS Vaccine Initiative (IAVI), New York, NY, United States of America; 8 IAVI, Johannesburg, South Africa; 9 IAVI, Human Immunology Laboratory, London, United Kingdom; 10 EMMES Corporation, Rockville, MD, United States of America; 11 Global BioSolutions, Melbourne, Australia; Glaxo Smith Kline, DENMARK

## Abstract

**Background:**

Sequential prime-boost or co-administration of HIV vaccine candidates based on an adjuvanted clade B p24, RT, Nef, p17 fusion protein (F4/AS01) plus a non-replicating adenovirus 35 expressing clade A Gag, RT, Int and Nef (Ad35-GRIN) may lead to a unique immune profile, inducing both strong T-cell and antibody responses.

**Methods:**

In a phase 1, double-blind, placebo-controlled trial, 146 healthy adult volunteers were randomized to one of four regimens: heterologous prime-boost with two doses of F4/AS01_E_ or F4/AS01_B_ followed by Ad35-GRIN; Ad35-GRIN followed by two doses of F4/AS01_B_; or three co-administrations of Ad35-GRIN and F4/AS01_B_. T cell and antibody responses were measured.

**Results:**

The vaccines were generally well-tolerated, and did not cause serious adverse events. The response rate, by IFN-γ ELISPOT, was greater when Ad35-GRIN was the priming vaccine and in the co-administration groups. F4/AS01 induced CD4+ T-cells expressing primarily CD40L and IL2 +/- TNF-α, while Ad35-GRIN induced predominantly CD8+ T-cells expressing IFN-γ +/- IL2 or TNF-α. Viral inhibition was induced after Ad35-GRIN vaccination, regardless of the regimen. Strong F4-specific antibody responses were induced. Immune responses persisted at least a year after the last vaccination. The complementary response profiles, characteristic of each vaccine, were both expressed after co-administration.

**Conclusion:**

Co-administration of an adjuvanted protein and an adenovirus vector showed an acceptable safety and reactogenicity profile and resulted in strong, multifunctional and complementary HIV-specific immune responses.

**Trial Registration:**

ClinicalTrials.gov NCT01264445

## Introduction

Although an effective prophylactic HIV-1 vaccine is likely to require the induction of broad and potent Env-specific antibody responses, CD8+ T lymphocyte responses that control HIV replication and CD4+ T lymphocytes that help generate and maintain HIV-specific cellular and humoral responses may also be necessary. Many T-cell based vaccines assessed in humans induce responses that are skewed to either CD4+ or CD8+ T-cell responses [1]. Cellular immune responses are critical in containing viral load; CD8 T cells generated within days of HIV infection result in lowering viral loads and slowing the rate of CD4+ T-cell decline. In long-term non-progressors, CD8+ T cells with multiple functions appear to control viral load for extended periods of time [2–4]. The important role of T cells in control of SIV infection has been demonstrated in multiple non-human primate studies and confirms what has been seen in humans, moreover depletion of T cells in SIV-infected macaques leads to uncontrolled viremia [5]. Finally, potent CD8+ T cell responses induced by vaccination of macaques have led to dramatic reduction of SIV to undetectable levels in infected animals [6]. In future development, a regimen capable of inducing CD4+ and CD8+ T cell responses would be combined with an HIV envelope (Env) immunogen to induce neutralizing and/or non-neutralizing functional antibodies.

Phase 1 studies in Europe and the US, respectively, suggest that the F4 HIV vaccine (clade B p24, RT, Nef, p17 fusion protein) formulated with the AS01 adjuvant system has an acceptable safety and reactogenicity profile and induces robust CD4+ T-cell response and antibody responses in HIV-1-uninfected volunteers and HIV-1-infected patients [7, 8] and that the Ad35-GRIN vaccine (expressing clade A Gag, RT, Int and Nef) is safe and induces a robust CD8+ T-cell response [9]. Adenoviral vectors can effectively transduce host cells and induce high magnitude CD8+ T cell responses in a high proportion of vaccinees, without production of infectious adenovirus or integration into the host genome [10–12]. Several observations have shown that Adenoviral vectors are a good prime for T-cell response but the mechanism is not yet understood [13, 14]. Because immunity to Ad35 varies among populations and could affect vaccine responses, safety and immunogenicity was evaluated in participants without preexisting Ad35 immunity. We hypothesized that the combination—either sequential prime boosts or co-administration of F4/AS01 and Ad35-GRIN—might induce complementary HIV-1 specific CD4+ and CD8+ T-cell responses. We also evaluated if the order of administration (i.e. Ad35-GRIN both as a prime for F4/AS01 and as a boost) influenced the quality of T cell response, as well as the quantity of antibodies produced. This paper summarizes the evaluation of several regimens, with a view to constructing the ideal future HIV vaccine candidate, combining T- and B-cell immunogens that will induce optimal responses in both arms of the immune response.

## Materials and Methods

### Ethics and regulatory approval

The study protocol was approved by the ethics committees of Kenyatta National Hospital, University of Nairobi, Uganda Virus Research Institute, University of Zambia and Emory University, and reviewed by the responsible regulatory authorities in each country. Each study participant provided written informed consent prior to undertaking any study procedures.

### Participants and study design

**Fig 1:**
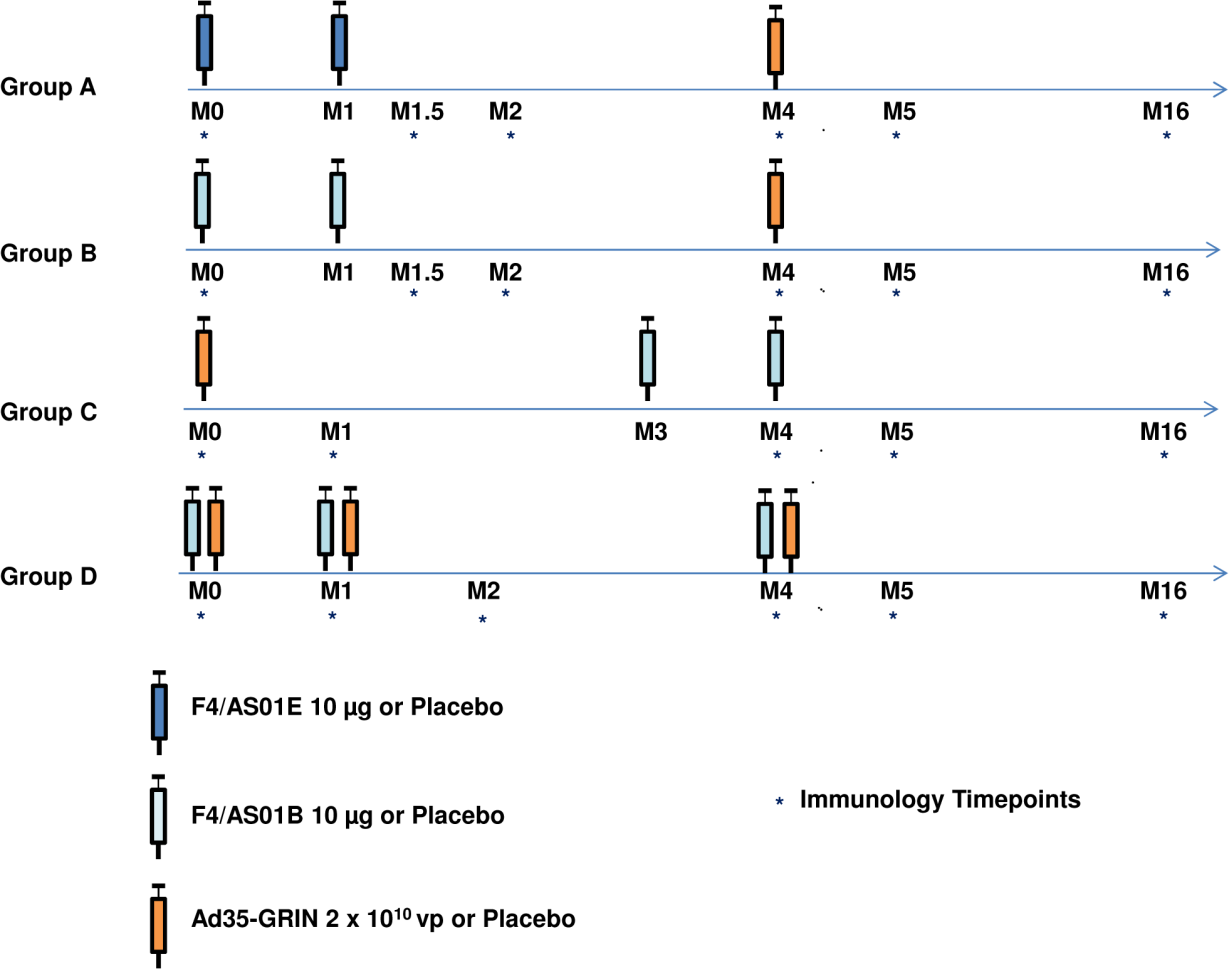
Study Schema. Enrolled volunteers were randomized to one of the 4 regimens (groups A, B, C, or D), with approximately 28 vaccine and 7 placebo recipients per group, receiving 0.5 mL (F4/AS01_E_ or F4/AS01_B_) and/or 1.0 mL (Ad35-GRIN) injections of vaccine or placebo. Vaccinations were given at baseline, month 1 (M1) or month 3 (M3), and month 4 (M4).

Eligible adults were recruited at centers in Uganda, Kenya and Zambia using informational seminars. The first screening was on 10 Jan 2011, the first enrolment was on 28 Feb 2011, the last enrolment was 13 Aug 2011, and the last follow-up was on 28 Feb 2013. Volunteers were healthy, aged 18–40 years, at lower risk for HIV infection with confirmed negative serology for HIV-1 and HIV-2 infection, willing to use an effective method of contraception, and testing negative for Ad35-specific neutralizing antibodies (EC90 titer <16). Women were not pregnant and not lactating. Volunteers with chronic medical disease, including hepatitis B or C infection, were excluded. Volunteer comprehension of the study was ascertained using an assessment of understanding tool with true/false questions. Volunteers were screened up to 42 days before vaccination (up to 90 days for Ad35 neutralizing antibody), and followed for 64 weeks post first administration. Allocation schedules were computer generated. Investigators, volunteers, laboratory personnel and clinical monitors were blind to treatment assignments. Ongoing HIV risk assessments and prevention counseling were offered to volunteers during the trial. The study was a multi-center, double-blind, randomized, placebo-controlled phase 1 trial of heterologous prime-boost or co-administration 3-dose regimens (Fig 1). Enrolled volunteers were randomized to one of the 4 regimens (groups A, B, C, or D), with approximately 28 vaccine and 7 placebo recipients per group, receiving 0.5 mL (F4/AS01) and/or 1.0 mL (Ad35-GRIN) injections of vaccine or placebo in the deltoid muscle of the non-dominant arm. Vaccinations were given at baseline, month 1 or month 3, and month 4. In the co-administration group (D), the F4/AS01 and Ad35-GRIN vaccines or placebos were administered into the same deltoid muscle, approximately 2–4 cm apart.

#### Study Vaccines

The F4/AS01 HIV candidate vaccine consists of 10 μg of F4, a lyophilized recombinant fusion protein expressed in Escherichia coli and comprising 4 HIV-1 clade B antigens: p24 (BH10), RT (reverse transcriptase) (HXB2) mutated to remove the RT polymerase activity, Nef (Bru-Lai), and p17 (BH10). The vaccine antigen was prepared as a lyophilized pellet containing F4 in sucrose, ethylenediaminetetraacetic acid, arginine, polysorbate 80, and sodium sulfite in phosphate buffer. The F4 vaccine was manufactured according to the principles of Good Manufacturing by Practices (GMP) by GlaxoSmithKline Biologicals, Rixensart, Belgium. The AS01_B_ adjuvant is an Adjuvant System containing 50 μg 3-*O*-desacyl-4’-monophosphoryl lipid A (MPL), 50 μg QS-21 (*Quillaja saponaria* Molina, fraction 21; Antigenics Inc., a wholly owned subsidiary of Agenus Inc., Lexington MA, USA) and liposomes. AS01_E_ contained half the quantity of immunostimulants of AS01_B_. F4 was reconstituted in the AS01 liquid adjuvant immediately prior to vaccination and administered as 0.5mL containing 10 μg of F4/AS01 by intramuscular injection.

The Ad35-GRIN is a recombinant replication-defective adenovirus serotype 35 containing HIV -1 subtype A gag, RT, integrase, and nef genes (abbreviated as GRIN). The genes were designed as a fusion product, and codon optimized for human cell expression and translation. Mutations were introduced into the sequence to abrogate functional activity. Ad35-GRIN vaccine was produced in HER96 cells by Transgene (Strasbourg, France) according to the principles of GMP. The vaccine was prepared in formulation buffer composed of Tris 10 mM pH 8.5, Sucrose 342.3 g/L, 1mM MgCl_2_, Tween80 54 mg/L and 150mm NaCl in water for injection and administered as 1.0mL containing 2x10^10^ viral particles by intramuscular injection. The placebo was saline (NaCl 0.9%), produced and released by GSK Vaccines; this was given as 0.5mL or 1.0mL by intramuscular injection.

### Safety Assessments

Safety and tolerability were assessed clinically and by routine laboratory tests. Volunteers recorded local and systemic reactogenicity on memory cards for 14 days post-vaccination, which were reviewed by clinicians. Data on spontaneously reported adverse events were collected during the vaccination period. Laboratory safety assessments, including full blood count and chemistries, were performed on the day of vaccination, 7 and 28 days after each vaccination, at week 36 and at end of study. Urinalysis was performed on the day of vaccination, 14 days after each vaccination and at end of study. Women were not vaccinated unless the urine pregnancy test was negative prior to each vaccination. Safety testing was done at the respective study center laboratories. All safety data were graded according to the Division of AIDS Table for Grading the Severity of Adult and Pediatric Adverse Events (DAIDS AE Grading Table), Version 1.0, December 2004. Serious adverse events (SAEs) were defined in accordance with International Conference on Harmonization—Good Clinical Practice (ICH-GCP) guidelines. HIV-1/2 EIAs (Vironostika HIV Uni-Form II Ag/Ab, Bio Merieux) were performed at weeks 4 or 12, 16, 20, 36, 48, and 64. Plasma from volunteers with reactive results was tested for HIV infection using the Abbott real time PCR kit (Abbott Molecular).

During the preclinical assessment of F4/AS01_B_, while tested together with a DNA based vaccine with or without Imiquimod application, lens opacities were noted in a minipig model, however the relationship to F4/AS01_B_ vaccination was not clear. No lens opacities were observed in a repeated toxicological study in rabbits. Despite a weak biological plausibility, the inconclusive nature of this preclinical observation led to further ophthalmological evaluations in all subsequent clinical trials investigating F4/AS01_B_, alone or in combination with other vaccine components. In the present study ophthalmologic examination with slit-lamp was performed at baseline and at the end of the study at two of the four study centers (48% of volunteers).

### Immunogenicity Assessments

All immunogenicity assays were performed in a blinded fashion under Good Clinical Laboratory Practices (GCLP) [15, 16]. Samples were collected at baseline (M0) and at last study visit (M16) for all groups, and depending on the regimen—2 weeks and 1 month after the 2^nd^ F4/AS01 (M1.5 or M2 or M5) and 1 month after Ad35-GRIN administered alone (M1 or M5) (Fig 1). Peripheral blood mononuclear cells (PBMC) were isolated using density gradient separation from heparinized whole blood, frozen in a mixture of fetal bovine serum (Sigma-Aldrich, St Louis, MO, USA) and DMSO (90:10 ratio) using a Kryo 560–16 rate controlled freezer (Planer, Sunbury-On-Thames, UK). PBMC were stored and shipped in vapor phase liquid nitrogen to the central testing laboratories (IAVI Human Immunology Laboratory, Imperial College, London and CEVAC, Ghent, Belgium) as previously described [7, 9, 17].

#### Interferon-gamma (IFN-γ) ELISPOT assay

Cellular immunogenicity was assessed by IFN-γ ELISPOT as previously described [9]. PBMC were thawed, overnight rested and counted using a Vi-Cell XR counter (Beckman Coulter, UK). The PBMC for the IFN-γ ELISPOT assay were plated at 2 x 10^5^ viable cells per well in quadruplicate with peptides at 1.5μg/mL representing the vaccine inserts as described previously [9, 18]. Peptide pools of 15-mer peptides overlapping by 11 amino acids with 90% purity by HPLC covering the sequences of Clade B p17, p24, RT, or Nef matched F4 antigens (Eurogentec, Belgium) or Clade A gag, RT, Int or Nef matched GRIN antigens (AnaSpec Inc, Fremont, CA) were used. A cytomegalovirus (CMV) pp65 peptide pool (quality control), phytohaemagglutinin **(**PHA) at 10μg/mL and a mock stimulus (DMSO/medium) were also used as previously described [18]. Spot forming cells (SFC) were counted using an automated AID ELISPOT reader (Autoimmun Diagnostika, Strassberg, Germany). HIV-1-specific T-cell responses were expressed as the frequency of SFC and percentage of responders to F4, GRIN, individual vaccine components or any peptide pool. A positive response was defined as the average number of background-subtracted spots of >38 SFC/m PBMC for each peptide pool and had to satisfy all quality control criteria [18]. Sample integrity was excellent across all sites with a median viability of 97.3%. CMV and PHA responses were consistent across time for each volunteer.

#### T-cell responses by intracellular cytokine staining (ICS)

HIV-1-specific CD4+ and CD8+ T-cell responses were evaluated by intracellular cytokine staining (ICS) to assess the expression of interleukin-2 (IL-2), interferon-γ (IFN-γ), tumor necrosis factor-α (TNF-α) and CD40-ligand (CD40L) using frozen PBMC isolated from venous blood [7]. A viability marker (LIVE/DEAD, Molecular Probes, Eugene, OR, USA) and CD3 marker were added to the staining panel. The same vaccine insert-matched peptides that were used in the ELISPOT assay were used for ICS stimulation. CMV-pp65 peptide pool was used as quality control. HIV-1-specific T-cell responses were expressed as the frequency of total CD4+ T-cells co-expressing CD40L (denoted as CD40L+ CD4+ T cells) and at least one cytokine or total CD8+ T-cells expressing at least one cytokine (IL-2, TNF-α or IFN-γ), the cytokine co-expression profile and the percentage of responders to F4, GRIN and individual vaccine components. If cytokine secretion was undetectable at pre-vaccination, then a subject was considered a responder if the proportion of CD40L+CD4+ T-cells or CD8+ T-cells expressing at least one cytokine was ≥ the assay cut-off. The cut-off was based on the 95th percentile of all volunteers at pre-vaccination (rounded to the next 0.01—with a min 0.03%). In subjects with detectable cytokine secretion at pre-vaccination, response was defined as a greater than 2-fold increase in CD40L+CD4+ T-cells or CD8+ T cells expressing at least one cytokine from baseline. Background values (culture with no peptides) were subtracted from HIV-1 specific values. For the ICS, the F4- and GRIN-specific CD4+ and CD8+ T-cell responses were estimated from the sum of the specific CD4+ and CD8+ T-cell frequencies in response to each individual antigen.

#### Viral inhibition assay (VIA)

A VIA assay qualified for use in vaccine trials as described previously was used [19]. VIA activity was assessed only in Groups B-D at three (B and C) or 4 timepoints (D). Briefly, antibody-expanded pre-vaccination CD4+ T cells were infected with a panel of HIV-viruses and cultured with pre- and post-vaccination antibody-expanded CD8+ T cells. The following HIV-1 isolates were used along with the subtype and Genbank accession numbers where known in parenthesis; IIIB (K03455, subtype B), ELI (K03454, subtype A/D), U455 (M62320, subtype A), 97ZA012 (AF286227, subtype C), CH77 (JN944909, subtype B), CH106 (JN944897, subtype B), 247FV2 (subtype C, generously donated by George Shaw, University of Birmingham, Alabama) and CBL-4 (formerly RUT, subtype D). The following viruses were obtained through the NIH AIDS Reagent Program, Division of AIDS, NIAID, NIH; HIV-1 97ZA012 from the UNAIDS Network for HIV Isolation and Characterization, HTLV-IIIB/H9 from Dr. Robert Gallo and HIV-1 ELI from Dr Jean-Marie Bechet and Dr Luc Montagnier. HIV-1 CBL-4 (provided by Dr Paul Clapham and Professor Robin Weiss) and HIV-1 U455 (provided by Dr R Downing) were obtained from the Centre for AIDS Reagents, National Institutes of Biological Standards and Control, UK). CD8+ T-cell-mediated inhibition was expressed as the log_10_ reduction in p24 content of CD8+ and CD4+ T-cell co-cultures, compared with infected CD4+ T cells alone. The threshold used for positive inhibition was determined from previous validation studies as reduction in measurable p24 production of >1.5 logs, the pre-vaccination response for the same virus must be negative (i.e., not cross-reactive) and the difference between the post-vaccination and pre-vaccination response should be ≥0.6 log_10_ inhibition.

#### Humoral Immune Response to vaccine F4 antigens (ELISA)

Immunoglobulin G (IgG) antibody titers to F4, p17, p24, RT and Nef were analyzed using standard in-house enzyme-linked immunosorbent assays (ELISA) as previously described [7]. The cut-off for seropositivity was ≥326 mELISA units (mEU)/ml for p17, ≥249 mEU/ml for p24, ≥386 mEU/ml for RT, ≥1722 mEU/ml for Nef and ≥133 mEU/ml for F4. For the antibody response to F4 antigens (ELISA), seropositivity rates and geometric mean antibody concentrations (GMCs) for each individual antigen and the F4 fusion protein were calculated with 95% CIs. For seropositivity rates, 95% CIs were computed using the exact method for binomial variables. The 95% CIs for GMCs were calculated by taking the anti-log of the 95% CI of the mean log10-transformed antibody values. For each individual F4 antigen and the fusion protein, antibody concentrations below the cut-off of the assay were given an arbitrary value of half the cut-off for the purpose of GMC calculation.

#### Ad35 Neutralizing Antibody Assay

Anti-Ad35 neutralization titers were measured using heat-inactivated serum samples at screening (the presence of pre-existing antibody to Ad35 was a criterion for exclusion), from 4 weeks after the first vaccination and 2 weeks after the second vaccination in a previously described, qualified cell-based assay [20]. Anti-Ad35 titers were calculated as the serum dilution allowing a 90% reduction of luciferase activity in infected cells (EC90). An EC90 cut-off of 16 (reciprocal of serum dilution) was set where a positive response was defined as EC90 ≥ 16 and a negative response as EC90 < 16.

### Sample Size and Pause Rules

#### Safety interim analyses

Blinded summary tables and listings of adverse events, including solicited reactogenicity events, were presented to an independent Safety Review Board (SRB). The SRB reviewed the blinded study data for the first 28 volunteers in groups A/B, for the first 14 volunteers in groups C and for the first 14 volunteers in group D at 2 weeks post month 1, and 2 weeks post month 4 vaccinations.

#### Randomization and blinding

Volunteers were randomized to vaccine or placebo in a 4:1 ratio, using a block size of 5, stratified according to site. The randomization schedule was prepared by statisticians at the data coordinating center, EMMES Corporation. The randomization list was sent to the site pharmacist of record for dispensing of vaccine and placebo assignments. Investigators at the study sites enrolled volunteers via an electronic enrollment system (administered by the data coordinating center), where allocation codes were assigned consecutively to eligible volunteers at the time of first vaccination.

Study staff (with the exception of the pharmacist), volunteers and laboratories were blinded with respect to volunteer assignment between adjuvant groups A vs. B as well as to the allocation of active study vaccine or placebo within each group. There was no blinding between group schedules A/B, C and D. Volunteers in Groups C or D knew their specific group assignment and were blinded only with respect to the administration of vaccine or placebo.

### Statistical Analysis

All study volunteers receiving at least one dose were included in the safety analyses. Two vaccine recipients that were confirmed by HIV RNA PCR testing to be HIV-infected during the study were excluded from the immunogenicity analysis, but included in the safety analysis until diagnosis. The proportions of volunteers with local and systemic reactogenicity for 14 days after each dose were summarized by treatment group. Frequency of reactogenicity and specific adverse events in the vaccine and placebo groups were compared by Fisher’s exact test. Immunogenicity results were summarized within each group at each time-point using descriptive statistics for continuous variables and percentages (with 95% CI) for categorical variables. The study was initially powered to demonstrate the non-inferiority of the immune response induced by F4/AS01E compared to F4/AS01B. The criterion used was the following: the upper limit of the 95% CI for the ratio of the magnitude of the CD40L+ CD4+ T cells expressing at least IL-2, between both groups at 2 weeks post second vaccination (Month1.5) should be below 1.5. Other exploratory comparisons were conducted between the vaccine groups.

Statistical analyses were performed with SAS software, version 9.2 (SAS Institute, Cary, NC). A two-sided *p* value of less than 0.05 was considered to indicate statistical significance.

## Results

### Volunteer Disposition

**Fig 2:**
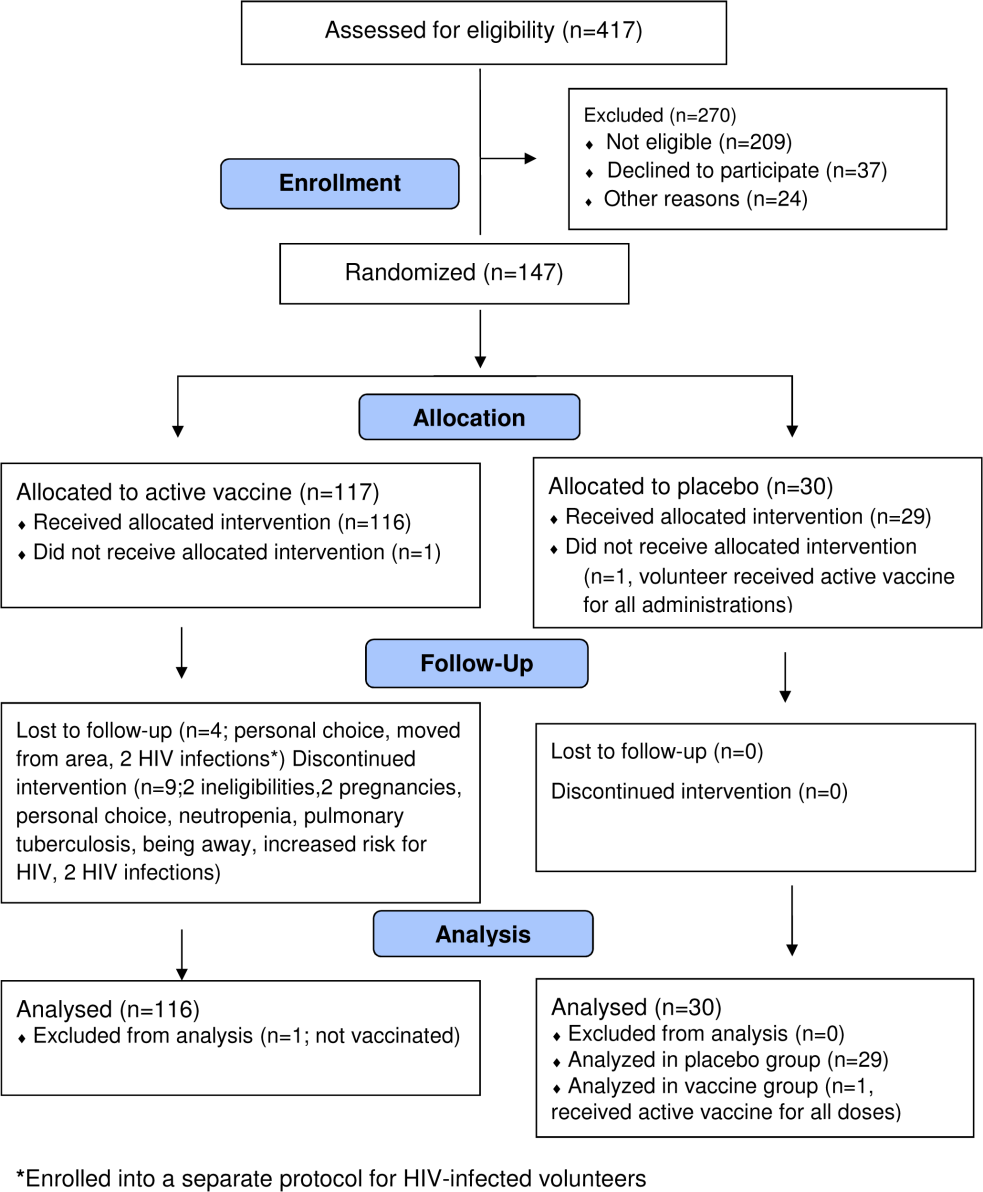
CONSORT Flow Diagram. Number of individuals assessed for eligibility, enrolled and randomized to study vaccine(s) and respective placebo, followed-up and analyzed.

From January 2011 through July 2011, 417 potential volunteers were screened for eligibility (Fig 2). Of 147 randomized study volunteers, 146 received ≥ 1 dose of vaccine or placebo, 141 (97%) received two vaccinations and 137 (94%) received all vaccinations. One volunteer randomized to a vaccine group did not receive any doses and one volunteer randomized to the placebo arm accidentally received vaccine, and was analyzed as part of the vaccine group. The placebo and vaccine groups were similar in age and gender (Table 1). A total of 142 volunteers (97%) completed 64 weeks follow-up of the study. No discontinuations or withdrawals were due to vaccine-related adverse events (Fig 2). All treated study volunteers were included in the safety analysis. Two vaccine recipients, confirmed by HIV RNA PCR testing to be HIV-infected during the study, were excluded from the immunogenicity analysis but were included in the safety analysis until diagnosis.

**Table 1:** Baseline characteristics by treatment group.

		Placebo	A (FE,FE,A)	B (FB,FB,A)	C (A,FB,FB)	D (A+FB x3)	Total
No of Volunteers		29	31	29	28	29	146
Sex	Female	12 (41.4%)	12 (38.7%)	7 (24.1%)	8 (28.6%)	13 (44.8%)	52 (35.6%)
	Male	17 (58.6%)	19 (61.3%)	22 (75.9%)	20 (71.4%)	16 (55.2%)	94 (64.4%)
Race	Black	29 (100.0%)	31 (100.0%)	29 (100.0%)	28 (100.0%)	29 (100.0%)	146 (100.0%)
ge (yrs)	Mean	27.6	25.8	27.4	24.3	27.1	26.5
	Range	18–38	19–39	19–39	18–35	18–37	18–39
Vaccinations Received	*Vac. #1	29 (100.0%)	31 (100.0%)	29 (100.0%)	28 (100.0%)	29 (100.0%)	146 (100.0%)
	Vac. #2	29 (100.0%)	31 (100.0%)	27 (93.1%)	26 (92.9%)	28 (96.6%)	141 (96.6%)
	Vac. #3	29 (100.0%)	31 (100.0%)	26 (89.7%)	25 (89.3%)	26 (89.7%)	137 (93.8%)

*Vac. = vaccination.

### Protocol Deviations

There were 190 protocol deviations in this study, mostly minor deviations involving protocol-specified study visit windows or schedule compliance or involving isolated inability to obtain complete collections of biological specimens (i.e., shortage of blood volume due to poor venous access). One volunteer was incorrectly administered vaccine and was analyzed with the vaccine group. One enrolled volunteer did not meet the eligibility requirements and was discontinued after the first vaccination. PBMCs were not collected at one of the clinical centers at Day 42. A symptom-directed physical exam was conducted at one of the clinical centers at the final visit, instead of protocol specified general physical exam, but general exams were performed once the deviation was identified. No protocol deviations were recorded as resulting in adverse events. The interpretation of the data presented here is not affected by the protocol deviations.

### Safety and Tolerability

**Fig 3:**
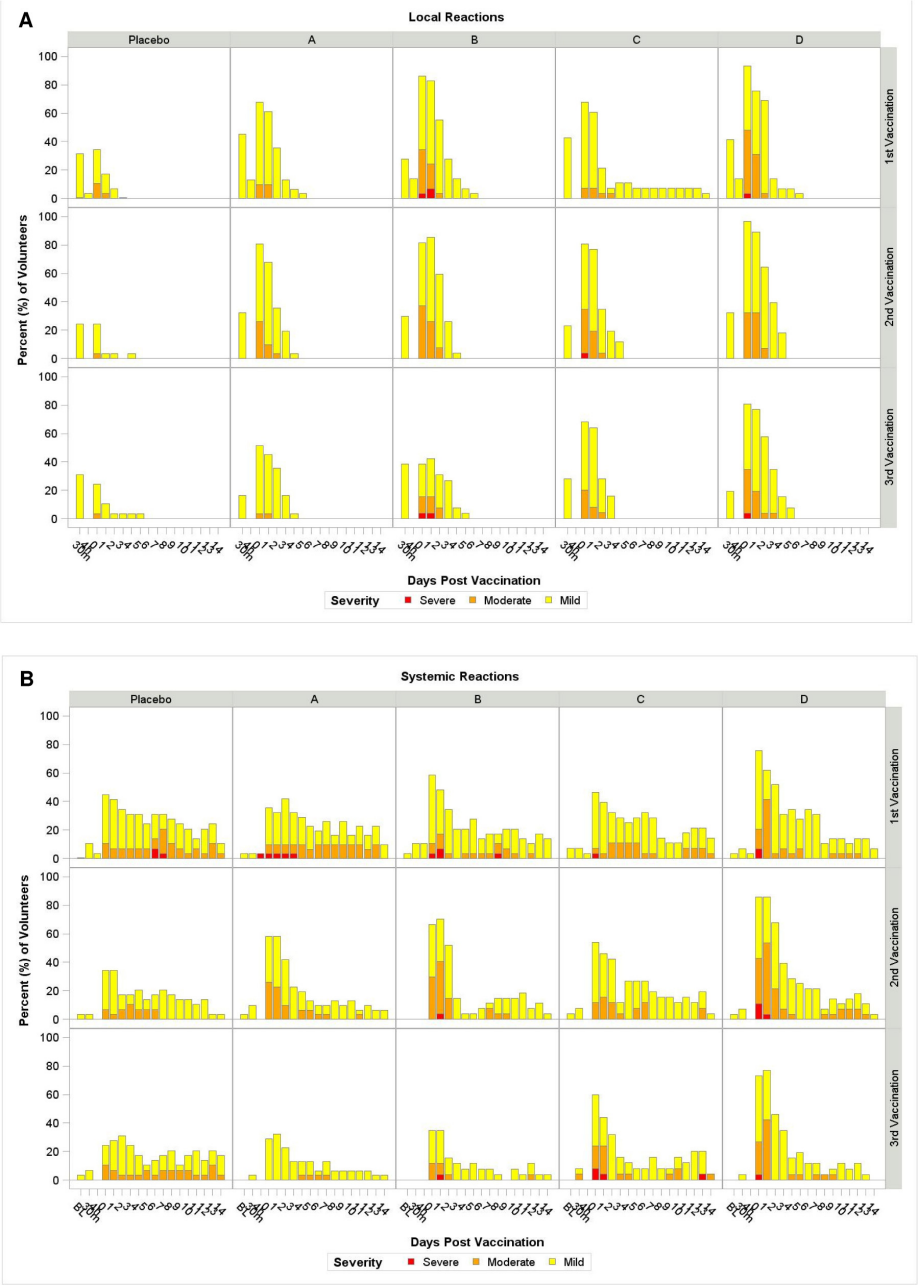
Time Course of Local and Systemic Reactions by group. The Y-axis represents the percentage of volunteers experiencing reactogenicity events. Panel **A** for local reactions and panel **B** for systemic reactions post first, second and third vaccinations with upper, middle and lower rows respectively for each group. The X-axis represents the days of occurrence of the events, Day 0 being the day of vaccination. Volunteers did a self-assessment of reactogenicity with a memory card on Day 0 (evening of vaccination) and daily through Day 14. The figure shows the maximum severity assessment grade recorded as per the volunteer’s and clinic’s assessments combined. The severity grade of the reactogenicity events is indicated by color codes (mild: yellow; moderate: orange; severe: red).

No vaccine-related serious adverse events occurred in any group (95% CI, 0%- 3%). All placebo recipients were grouped together for analysis. Overall, the vaccines were well tolerated in all groups. There were no statistically significant differences in moderate or worse local or systemic reactogenicity events among treatment and placebo groups. Injection site reactions were common after both F4/AS01 and Ad35-GRIN (Fig 3). Pain and tenderness were the most common local reactions. The majority were mild or moderate. Five volunteers had severe (Grade 3) pain and/or tenderness; all events were self-limited and resolved in 1–5 days (S1 Text). Systemic reactogenicity was also common in all groups, including the placebo group. Chills, headache, malaise, fatigue, myalgia and arthralgia were the most common systemic events. Most were mild or moderate. Nineteen volunteers had severe (grade 3) reactions: 9 after F4/AS01, 1 after Ad35-GRIN, 6 after co-administration, one after Ad35-GRIN and again after F4/AS01, and 2 among placebo recipients (Supplementary data). All events were transient and resolved spontaneously. There were no vaccine-related severe or very severe clinical adverse events or laboratory abnormalities. There was no evidence of vaccine-induced seropositivity as measured by the 4th generation HIV Ag/Ab ELISA. At entry into a long-term follow up study, two rapid HIV tests were used (Alere Determine HIV 1/2 and Trinity Uni-Gold). Two vaccine recipients, one in Group A and one in Group C had reactive Alere Determine results but no evidence of HIV infection by RNA PCR.

HIV infection occurred in two vaccine recipients who received the co-administered vaccines (Group D), after receiving all vaccinations. One volunteer reported a new sexual partner of unknown HIV status; the other reported no risk factors. Both remain clinically well with CD4+T-cell counts >500 without antiretroviral treatment.

### Immunogenicity

#### HIV-1 Specific Cellular Immune Responses ELISPOT and ICS

**Fig 4:**
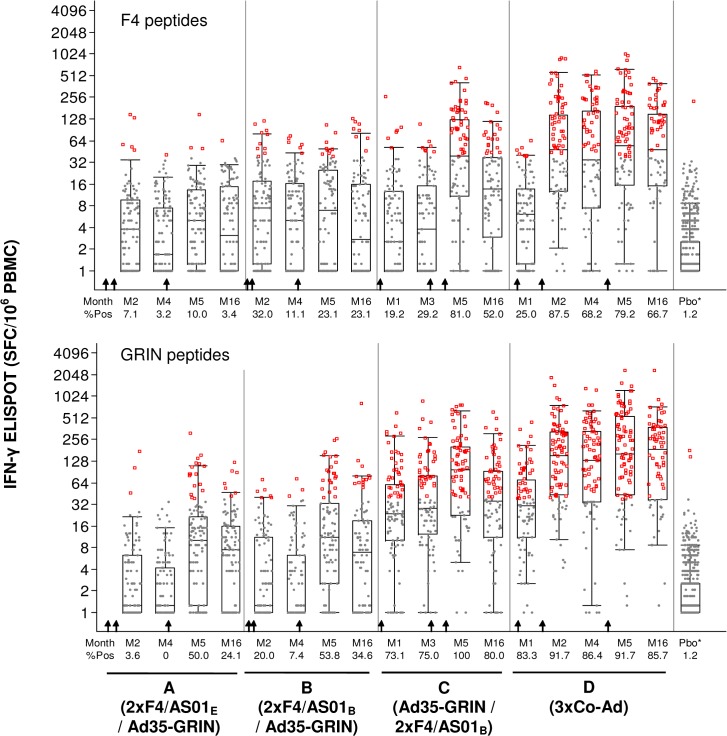
IFN-γ ELISPOT Response Magnitude to Any F4 and GRIN Antigens by Time Post Vaccination and Dose Groups. The y-axis shows the SFC/10^6^ PBMC on a half-log scale and the x-axis shows the time points post vaccination in months (M). **A.** F4 (any of p24+RT+Nef+p17 peptide pools) and **B.** GRIN (any of Gag+RT+Int+Nef peptides pools). Gray dots: response below the cut-off to any of the 8 peptide pools; red circles: response above the cut-off to any of the 8 peptide pools. For the vaccine groups, the overlaid box plot summarizes the overall responses (i.e., the median, 1st and 3rd quartiles and Percentile 95^th^). All baseline and placebo (Pbo*) groups are combined in the far right box plot. The arrows indicate when the vaccines were given for each group (lower X-axis).

IFN-γ ELISPOT responses were detected in all groups: The co-administration group had higher responses to any antigen than the Ad35GRIN-F4/AS01 sequential group; both had higher responses than the F4/AS01-Ad35-GRIN groups (Fisher’s exact test; p<0.0001), regardless of adjuvant concentration (Fig 4A and 4B, S1 Table). In groups A-C the maximum (peak) geometric mean of all responses to any F4 or GRIN antigen occurred at Month 5 (M5), one month after last vaccination. For group D the response was similar at M5 and Month 16 (M16) one year after last vaccination. The overall response rate to any F4 and GRIN peptide pools at M5 was significantly greater in groups C (81% and 100%) and D (79 and 92%) than in groups A (10% and 50%) and B (23% and 54%). Likewise the overall response rate to any F4 and GRIN peptide pools at M16 was significantly greater in groups C (52% and 80%) and D (67 and 86%) than in groups A (3.4% and 24%) and B (23% and 35%). Amongst all 249 placebo and baseline samples, only 1 volunteer (0.4%) had positive responses, all at a single time point.

After the third vaccination at M5, overall the most frequent ELISPOT responses were to subtype A GRIN peptides, GRIN-RT was the most frequently recognized with 43, 35, 91 and 92% response rates in Groups A-D (S1 Fig). The next most frequent GRIN-specific responses were to clade A Gag and then Int and Nef. At M5, responses to subtype B F4 peptides showed a similar hierarchy where the most frequently recognized peptide was also RT with 10, 19, 81 and 75% response rates in Groups A-D.

**Fig 5:**
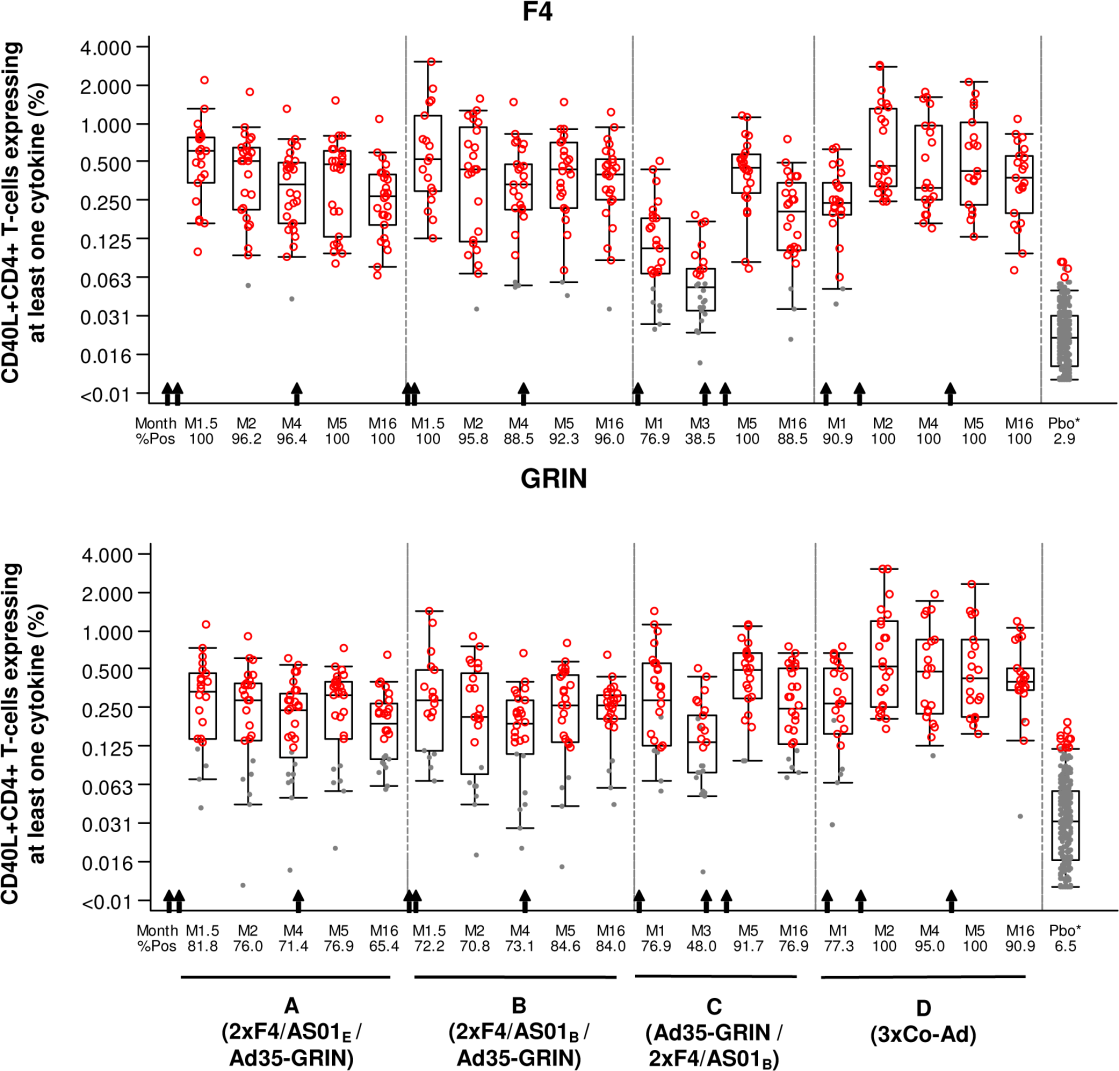
Kinetics of CD40L+CD4+ T-cell responses. The magnitude of the CD4+ T cells expressing CD40L and at least one cytokine among IL-2, TNF-α and IFN-γ is shown for **A.** F4 (any of p24+RT+Nef+p17 peptide pools) and **B.** GRIN (any of Gag+RT+Int+Nef peptides pools). M: Months. Gray dots: response below the cut-off to any of the 8 peptide pools; red circles: response above the cut-off to any of the 8 peptide pools. All baseline and placebo (Pbo*) groups are combined in the far right box plot. The arrows indicate when the vaccines were given for each group (lower X-axis).

**Fig 6:**
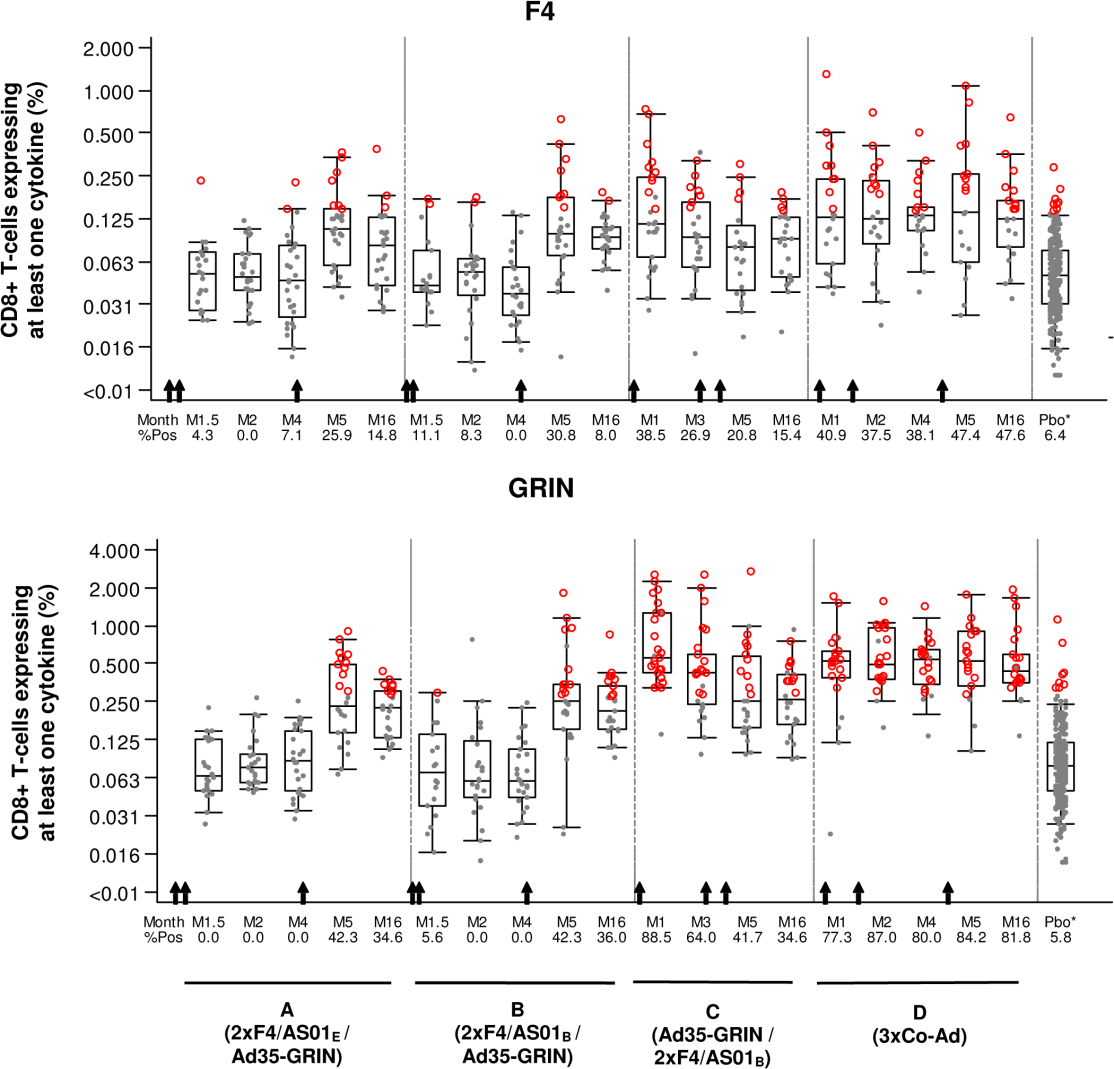
Kinetics of CD8+ T cell responses. The magnitude of the CD8+ T cells expressing at least one cytokine among IL-2, TNF-α and IFN-γ is shown for **A.** F4 (any of p24+RT+Nef+p17 peptide pools) and **B.** GRIN (any of Gag+RT+Int+Nef peptides pools). M: Months. Gray dots: response below the cut-off to any of the 8 peptide pools; red circles: response above the cut-off to any of the 8 peptide pools. All baseline and placebo (Pbo*) groups are combined in the far right box plot. The arrows indicate when the vaccines were given for each group (lower X-axis).

**Fig 7:**
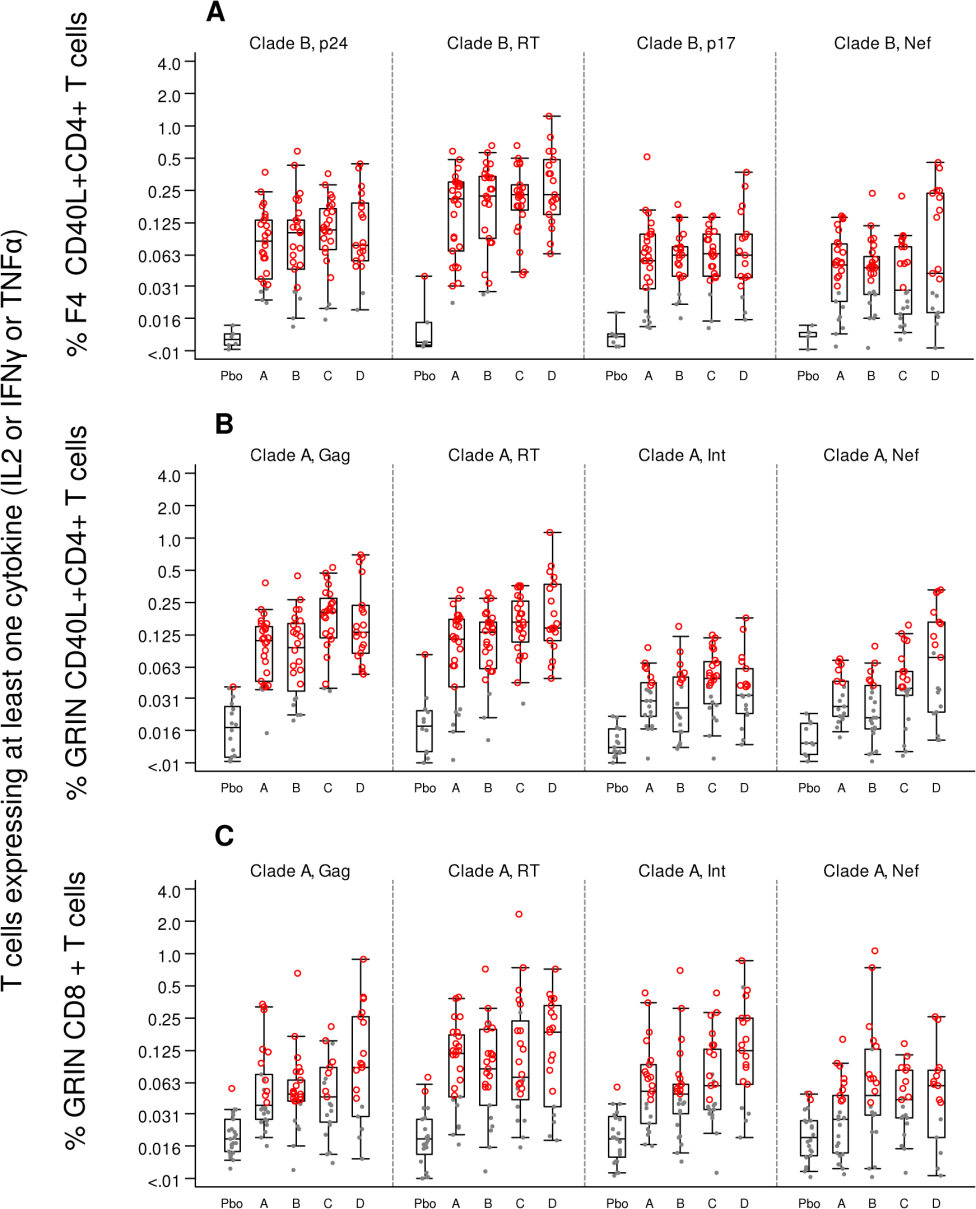
CD4 and CD8 responses to individual peptide pools 4 weeks after last vaccine (M5). The y-axis shows the magnitude of CD4+ and CD8+T-cells on a half-log scale across groups A-D for **A.** individual F4-specific CD40L+CD4+ T cell responses (clade B p24, RT, Nef and p17 peptide pools); **B.** individual GRIN-specific CD40L+CD4+ T cell responses (clade A Gag, RT, Int and Nef peptide pools) and **C.** individual GRIN-specific CD8+ T cell responses (clade A Gag, RT, Int and Nef peptide pools). The overlaid box-and-whisker plot summarizes the overall responses (i.e., the median, 1st and 3rd quartiles and 5^th^, 95^th^ Percentiles). Pbo; placebo groups were combined across groups A-D.

The magnitude of the ICS responses against F4 (sum of p24, RT, p17 and Nef peptide pools) and GRIN (sum of Gag, RT, Int and RT peptide pools) over all time points for CD4+ and CD8+ T cells is shown in Figs 5A, 5B, 6A and 6B and the percentage of responders is described in the S2 Table. In groups A and B at M1.5 and M2, F4/AS01 induced high levels of F4-specific CD4+ T-cells with cross-clade reactivity against Clade A GRIN peptides and with the percent specific responders to F4 and GRIN ranging from 96–100% and 71–82% respectively. In group C at M1, one vaccination with Ad35-GRIN induced fewer CD4+ T-cells and about half of the magnitude compared to immunization with two doses of F4/AS01 in groups A or B. For group D after the first co-administration at M1, CD4+ T cell responses were 91 and 77% respectively to F4 and GRIN peptides, and after the second co-administration, at M2, the responses went up to 100% for both. After the third vaccination (sequential or in co-administration) at M5, the CD4+ T-cell response rates were similar across groups A-D (ranged from 92–100% and 77–100% to F4 and GRIN pools respectively) with no evident difference between groups for the magnitude of F4- and GRIN-specific CD4+ T-cells. The CD4+ T-cell responses persisted up to M16, with an overall response rate of 89–100% and 65–91% for F4 and GRIN specific CD4+ T-cells respectively. As observed for the ELISPOT, F4- and GRIN-specific CD4+ T-cells were mainly directed to RT and Gag antigens (Fig 7). Despite similar trends in T-cell responses, the non-inferiority of the vaccine regimen containing the AS01_E_ adjuvant compared to AS01_B_ could not be formally demonstrated due to a subset of missing samples.

F4/AS01 alone induced marginal CD8+ T-cell responses, as observed in groups A and B at M1.5 and M2 (Fig 6A and 6B). Overall, the response rates and magnitude of the F4-specific CD8+ T-cell response were low or near baseline, whatever the groups and time points. In contrast, Ad35-GRIN induced high levels of CD8+ T-cells against GRIN antigens with little cross-reactivity to F4 pools with response rate of 89% and 39% respectively, as seen at M1. At M5, GRIN-specific CD8+ T-cell magnitude and response rates were highest in group D, which received three Ad35-GRIN administrations as compared to one dose in the other groups (84% response rate compared to ~42% in groups A-C) and dropped at M16 from 82% to ~35%. The CD8+ T-cells were mainly directed against Clade A RT, Int and Gag (Fig 7).

**Fig 8:**
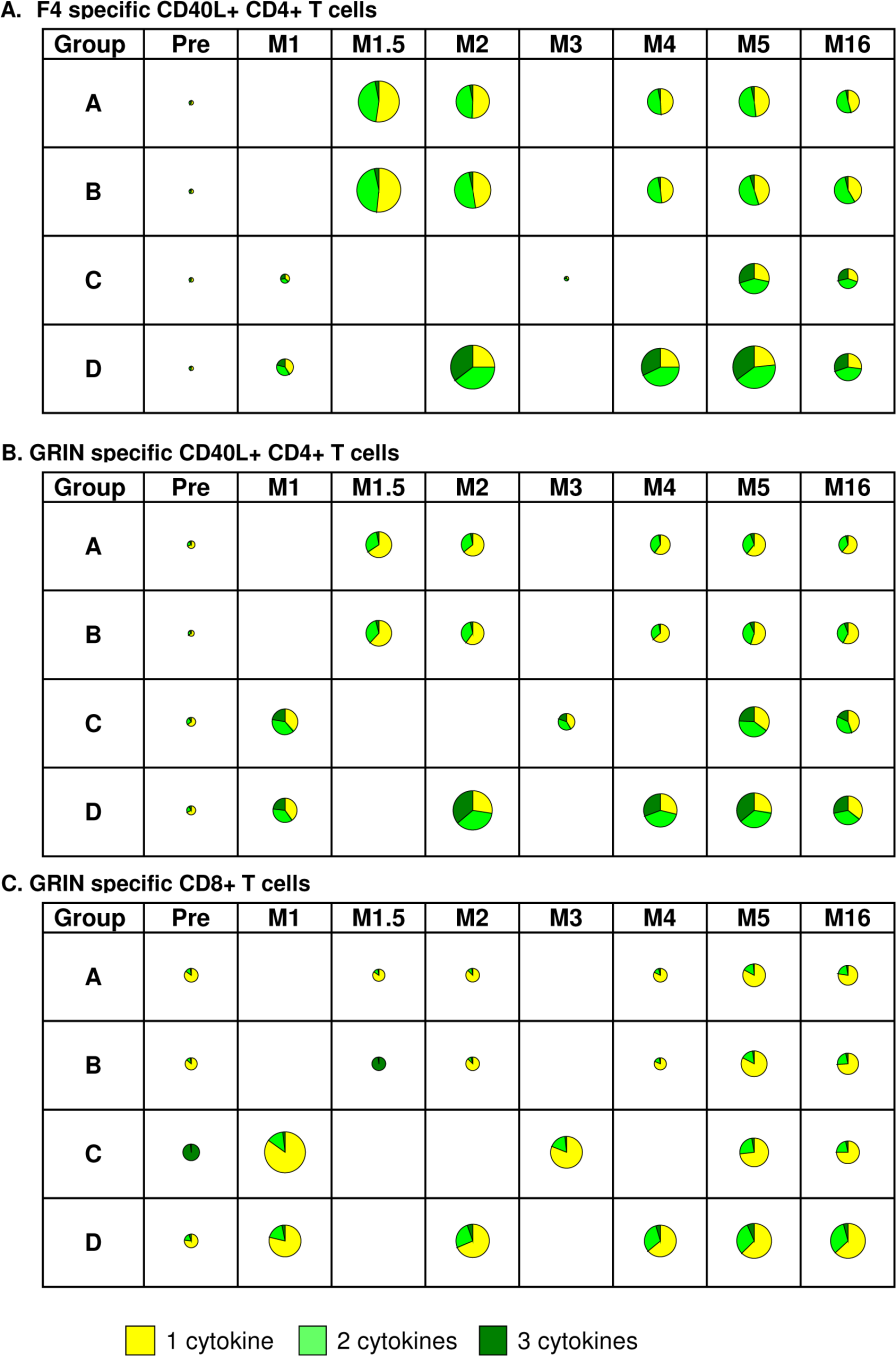
Multifunctional CD4+ and CD8+ T-cell responses. The diameter of each pie is scaled according to the magnitude (geometric mean). The pie charts represent **A.** CD40L+ CD4+ T cells expressing one, two or three cytokines to F4 (p24-RT-Nef- p17) peptide pools across groups A-D; **B.** CD40L+ CD4+ T cells expressing one, two or three cytokines to GRIN (Gag-RT-Int-Nef) peptide pools across groups A-D CD8+ T cells expressing one, two or three cytokines to GRIN (Gag-RT-Int-Nef) peptides pools across groups A-D and **C.** CD8+ T cells expressing one, two or three cytokines to GRIN (Gag-RT-Int-Nef) peptides pools across groups A-D. M: Months, Pre: pre-vaccination.

In groups A and B, vaccination began with F4/AS01, which induced mainly CD4+ T-cells expressing 1 or 2 cytokines (Fig 8A). These cells expressed IL-2 and IL-2 / TNF-α respectively as previously described [5] (data not shown). Boosting with Ad35-GRIN did not modify the cytokine response profile. In contrast, in group C after the initial vaccination with Ad35-GRIN, the majority of CD4+ T-cells were multifunctional at M1 (Fig 8B). These cells expressed IFN-γ and IL-2, alone or in combination, and about 1/3 of the cells expressed three cytokines (data not shown). Although the magnitude of the response was amplified after two booster doses of F4/AS01 the multifunctional profile was not modified at M5 (Fig 8A and 8B). In the co-administration group (Group D), high levels of multifunctional CD4+ T-cells were readily induced after two doses (Fig 8A and 8B). In all groups over all time points, GRIN-specific CD8+ T-cells expressed mainly one cytokine and to a lesser extent 2 cytokines (Fig 8C). These cells expressed IFN-γ alone or in combination with IL-2 or TNF-α (data not shown). As the magnitude of F4-specific CD8+ T cells was near to baseline, the functional profile is not shown. Whatever the groups, the respective T cell functional profiles observed at M5 were maintained up to M16.

#### Viral Inhibition

Viral Inhibition Activity (VIA) was assessed in a subset of randomly selected volunteers (10 vaccinees and 2 placebo) from Groups B-D at baseline and 4 weeks after vaccination (Table 2 and S2 Fig). None of the placebos had VIA above the cut-off at any timepoint. Overall, VIA was associated with Ad35-GRIN vaccinations. In group B, after two administrations of F4/AS01 VIA was detected in 2/8 volunteers (25%) and after the Ad35-GRIN boost, VIA activity was detected in 6/8 (75%) of volunteers respectively. In Group C, when Ad35-GRIN was administered as a prime followed by two F4/AS01 at 3 and 4 months, VIA activity was detected in 6/7 (86%) and 4/7 (57%) of individuals respectively. In Group D, when F4/AS01 and Ad35-GRIN were co-administered, VIA was detected in 5/8 (63%) after the first co-administration and in 7/9 (78%) after the 2^nd^ and 3^rd^ co-administrations.

**Table 2:** VIA response rate at 4 weeks after each Ad35-GRIN administration.

		Group B	Group C	Group D		
Virus	HIV subtype	M5	M1	M1	M2	M5
**247FV2**	C	*2/8 (25)	3/7 (43)	0/8 (0)	2/9 (22)	2/9 (22)
**97ZA012**	C	0/8 (0)	1/7 (14)	0/8 (0)	1/9 (11)	0/9 (0)
**CBL-4**	D	3/8 (38)	5/7 (71)	4/8 (50)	3/9 (33)	5/9 (56)
**CH077**	B	1/8 (13)	1/7 (14)	1/8 (13)	2/9 (22)	1/9 (11)
**CH106**	B	2/8 (25)	3/7 (43)	1/8 (13)	2/9 (22)	1/9 (11)
**ELI**	A/D	0/8 (0)	1/7 (14)	0/8 (0)	0/9 (0)	0/9 (0)
**IIIB**	B	3/8 (38)	5/7 (71)	5/8 (63)	5/9 (56)	4/9 (44)
**U455**	A	6/8 (75)	5/7 (71)	5/8 (63)	6/9 (67)	5/9 (56)
**ANY**		6/8 (75)	6/7 (86)	5/8 (63)	7/9 (78)	7/9 (78)

*Number of Volunteers positive over total tested (%)

The VIA response rates across Groups B-D were not significantly different from each other, but with the small group size, there was insufficient power for the comparison. Subtype B (IIIB) and subtype A (U455) were the most frequently inhibited viruses; for these 2 viruses, the onset of virus inhibition was clearly associated with the administration of the Ad35-GRIN (Table 2 and S2 Fig). VIA was also observed against subtype D (CBL-4) and less frequently to other viruses. For Groups B and C, highest VIA was seen at 1 month after Ad35-GRIN (M5 and M1 respectively) and for Group D VIA was detected after the first F4/AS01-Ad35-GRIN co-administration and remained at a similar level after each subsequent co-administrations (Table 2). VIA activity waned in group C at 5 months after the Ad35-GRIN and at M5, the overall median log inhibition (for all viruses) was significantly and marginally higher in Group B compared to Group C (p = 0.003) and Group D (p = 0.06). There was a similar breadth of VIA activity assessed by the number of viruses inhibited per volunteer with an average of 1.9, 3.1 and 1.8 viruses respectively across groups B-D (data not shown).

#### F4- Specific Antibody Responses

**Fig 9:**
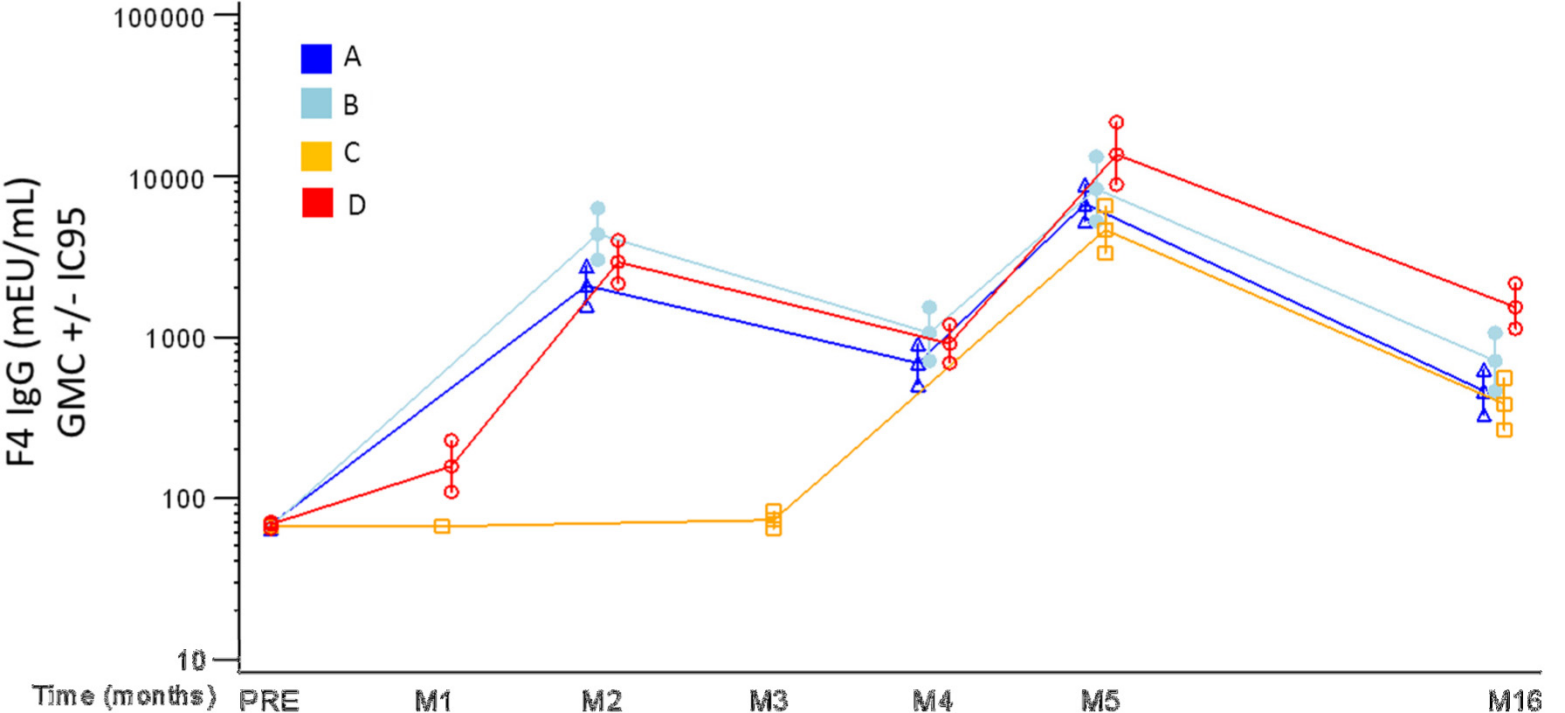
Kinetics of Humoral immune responses against the F4 fusion protein. Anti-F4 IgG antibody concentrations measured by ELISA expressed as geometric mean concentration (GMC) in mEU/ml across groups A-D. Group A = 2xF4/AS01_E_ / Ad35-GRIN, B = 2xF4/AS01_B_ / Ad35-GRIN, C = Ad35-GRIN / 2xF4/AS01_B_ and D = 3xCo-Ad. M = months.

F4 specific IgG binding antibodies were detected in 100% of individuals after the second vaccination in groups A, B and D (Fig 9). In Group C after a single Ad35-GRIN vaccination, the (cross-reactive antibody) response rate was 14% and, after 2 F4/AS01 administrations, the response rate reached 100% and the antibody titers were the same as those in groups A, B and D. At M16 the response rate was still high: 94%, 93%, 89% and 100% respectively for groups A, B, C and D (Fig 9). The binding IgG antibodies were detected against the four individual components of F4 antigen (p24, p17, RT and Nef) and conclusions were similar to F4-specific IgG antibodies (data not shown).

#### Ad35 Neutralizing Antibody Responses

The percentage and titers of antibodies that neutralized the Ad35 vector were similar in all groups: the response rates to one Ad35-GRIN administration were 13, 14, 15, and 15% in groups A-D respectively at 4 weeks, with a geometric mean titer (GMT) of the positive responders of 18, 38, 28 and 35 respectively (Table 3). Four weeks after the last vaccination (M5), response rates and titers were highest in the combined vaccine regimen, which had 3 rather than 1 Ad35-GRIN vaccinations. For Group D, the Ad35 neutralization response rate at 4 weeks after the 2^nd^ and 3^rd^ co-administration (M2 and M5 respectively) was 6/26 (23%) and 12/25 (48%) with GMT of 39 and 58 respectively among the positive responders. At M16 in Group D, 10/26 (38%) had a GMT of 35 among the positive responders. One volunteer in Group A/B who received placebo had a low Ad35 neutralization titer at M5 which was EC90; 19.7, just above the assay cut-off of 16.

**Table 3:** Ad35 Neutralization response rates and titers (EC90).

	Vaccine Group and month (M)							
	A	B	C	D				
	M5	M5	M1	M1	M2	M4	M5	M16
*Vaccinee	4/31 (13)	4/28 (14)	4/27 (15)	4/26 (15)	6/26 (23)	5/25 (20)	12/25 (48)	10/26 (38)
*Placebo	1/7 (14)	0/7 (0)	0/7 (0)	0/7 (0)	0/8 (0)	0/8 (0)	0/8 (0)	0/8 (0)
**Median [IQR] titer	17 [16–19]	20 [19–147]	26 [17–48]	26 [19–75]	24 [18–51]	19 [19–26]	59 [25–111]	26 [21–49]
**GMT (range)	18 (16–22)	38 (18–272)	28 (16–62)	35 (19–117)	39 (17–396)	31 (19–168)	58 (18–355)	35 (16–162)

*Number of Volunteers positive over total tested (%)

**Among positive vaccinee responders

## Discussion

Overall the combination of adjuvanted F4/AS01 and Ad35-GRIN was well-tolerated with an acceptable safety and reactogenicity profile. Both local and systemic reactogenicity were common; they differed little amongst regimens, were self-limited, and were comparable to reactogenicity reported with these products individually [7, 9]. There were no vaccine-related serious adverse events and no differences in moderate or worse adverse events among treatment and placebo groups. There was no evidence of vaccine-induced seropositivity as measured by 4th generation HIV Ag/Ab ELISA at trial completion or rapid HIV test kits in common use throughout Africa. At entry into a long-term follow up study, two rapid HIV tests were used (Alere Determine HIV 1/2 and Trinity Uni-Gold). Two vaccine recipients, one in Group A and one in Group C, had reactive results by Determine HIV test but no evidence of HIV infection by RNA PCR.

Adjuvanted F4/AS01 and Ad35-GRIN regimens induced high ELISPOT response rates, balanced CD4+ and CD8+ T-cell responses which persisted over time, and high titers of F4 binding antibodies. The vaccine regimens assessed in this trial were designed to induce multifunctional CD4+ and CD8+ T-cell responses to deal with viral escape and inhibit viral activity across clades. The regimens tested in this trial appeared to induce responses that were additive when compared with each individual component tested alone [7–9].

There is a reasonable consensus that both humoral and T-cell responses will be required for an effective HIV vaccine [21–25]. The Step and Phambili HIV vaccine efficacy trials were designed to elicit T-cell responses that might impact viral load; HVTN505 and RV144 were designed to elicit both T-cell responses and antibody responses [26–30]. None of these regimens had a significant impact on viral load or CD4+ count once participants became infected, and only RV144, a community based trial conducted in Thailand, showed protection against HIV acquisition with 31.2% efficacy [26–30]. For both RV144 and Step there was some indication of T-cell pressure exerted on some regions of the virus [31–34]. This small glimmer of hope supports the notion that vaccine-induced T cells could help control of HIV infection, as has been amply demonstrated in HIV infected individuals and non-human primates infected with SIV [2, 6]. The role of CD4+ T-cells in maintaining HIV-specific cellular and humoral responses has long been postulated with more recent data supporting this notion [35–37].

ELISPOT response rates in Group C (Ad35-GRIN + 2 F4/AS01) and Group D (3 co-administrations) were generally higher than those reported at peak immune response time points with other vaccine regimens tested in phase 1/2 trials and efficacy trials [12, 29, 38–40]. The magnitude of the response was modest but comparable to that seen with other vaccines using similar sample types, assays and analysis. Gag, Pol and Nef were each recognized to high frequencies and the responses persisted for at least a year. The high recognition of Gag, Pol and Nef may be due, in part, to the lack of HIV-Env in F4/AS01 and Ad35-GRIN vaccines. In the HVTN505 and related studies Env responses predominated [28, 38–40].

Multifunctional T-cell responses are present in HIV controllers, and such responses may be important for immune control of HIV. Balanced CD4+ and CD8+ T-cell responses were elicited by these vaccines, with profiles differing across regimens. F4/AS01 induced high levels of F4-specific CD4+ T-cells with cross-reactivity against Clade A and Ad35-GRIN induced CD4+ T-cells against Clade A with weak cross-reactivity against Clade B. Ad35-GRIN administered once in each of Groups A-C induced high levels of GRIN-specific CD8+ T-cells; a higher magnitude of CD8+ responses was maintained up to one year after three co-administrations in group D. The immune marker, CD40L is known as a costimulatory ligand required for T cell help and a marker for activated antigen-specific T cells [41–44]. Previous findings in healthy HIV-1-seronegative volunteers and HIV-1-seropositive volunteers showed that the CD4+T-cells induced by F4/AS01B vaccine co-expressed CD40L and IL-2 alone or in combination with TNF-α and/or IFN-γ [7, 45]. In this study, F4/AS01 induced mainly CD4+ T-cells co-expressing CD40L and one or two cytokines: IL2 and IL2/TNF-α, boosting with Ad35-GRIN did not modify the profile. In group C, the majority of CD4+ T-cells induced by one dose of Ad35-GRIN were multifunctional. In the co-administration group (Group D), high levels of multifunctional CD4+ T-cell responses were induced after 2 doses, and after a third dose were similar. Group D received 3-fold higher amount of Ad35-GRIN and 1.5 fold higher amount of F4/AS01 than the other groups which may have influenced persistence and polyfunctionality of CD4+ and CD8+ immune responses in this group. In all groups, the CD4+ T-cell response was maintained for at least one year after the last vaccination.

In this study, Ad35-GRIN was shown to be a better prime than boost i.e. higher CD8+ T cells frequency and higher polyfunctionality of CD4+ T cells. Adenoviral vectors appear to be good for priming immune responses as observed in HVTN 078 trial where NYVAC and Ad5 were evaluated. NYVAC was shown to be a better boost than prime for T-cell response as also seen in non-human primate studies [13, 14].

The response rates as detected by flow cytometry were generally greater than response rates detected by ELISPOT, the main reason for this is because the ELISPOT is based on IFN-γ production only whereas ICS detects responses to IFN-γ, Il-2 and TNF-α. Responses to F4/AS01 induce CD4+ T cells expressing mainly IL-2 and Ad35 induces polyfunctional CD4+ T cells and CD8+ T cells that express mainly IFN-γ. Thus differences between ELISPOT and ICS are more marked at 1M and 1.5M post F4/AS01B prime particularly for CD4-specific responses. When Ad35-GRIN is used as a prime or in the co-administration group D, the profile is more polyfunctional and therefore the proportion of T-cells (including CD4 and CD8) expressing IFN-γ increases and the difference in percent responses as detected by ICS and ELISPOT is less distinct.

Anti-HIV inhibitory capacity is considered to be an important attribute of CD8+ T-cells and such activities may help control HIV-viral load during acute and chronic infection particularly in long-term non-progressors [3, 4, 46–48]. In the present trial, viral inhibition activity (VIA) was associated with Ad35-GRIN vaccinations and was not induced by or enhanced by F4/AS01 alone. The VIA magnitude, response rate and breadth was similar to that seen in other HIV vaccine trials using a similar assay [19, 39, 49] but of lower magnitude than that seen in long-term non-progressors [3, 4, 46–48]. CD107α or granzyme B, two other important markers to characterize the **c**ytotoxicity of CD8+ T cells, were not prioritized in this study due to the limited blood volume.

Antibodies against F4-protein were detected in 100% of individuals in all groups. The high response rate and titers were driven by the F4/AS01 administration; Ad35-GRIN has previously been shown to induce only modest anti-Gag responses [7, 9]. The antibody response rates are similar to previous data with gp120, Nef and Tat administered with AS01 [50].

In a collaborative study, we carefully studied the seroprevalence of Ad5, Ad26 and Ad35 in the populations targeted for the current study [51]. The Ad35 platform was designed as an alternative to Ad5 vectors because of the lower seroprevalence and lower titers for Ad35 seen across the world. Ad35 is a human adenovirus serotype with low world-wide seroprevalence compared to Ad5 [51–53]. Across the 4 trial centers, 50/353 (14.2%) volunteers were screened out prior to enrollment in the trial because of pre-existing Ad35 neutralizing titers, all of which were low, as shown previously for East Africa [51]. Even after three co-administrations of F4/AS01+Ad35GRIN, only 13/27 (48%) of volunteers had Ad35 neutralizing antibodies, and these were of low titer [9]. Low seroconversion rates and low titers have been noted previously after Ad35 and Chimpanzee Adenovirus administration in humans [9, 54, 55]. The reasons for the differences in seroconversion rates and titers between different Ads are not clear, the Ad35 neutralizing assay may not be sensitive enough to detect very low titers or the assay does not pick up all neutralizing antibody epitopes. Higher vaccine doses of Ad35 or Chimp Ad administered to humans increases the vaccine take and titer in some volunteers. However, some volunteers still did not have a response to the vector but did have a response to the insert which may suggest HLA or other immune response associated with Ad vector responses [9, 54].

Ad35 vectors also have a different serotype, cellular receptor and innate and immune signaling mechanisms compared to Ad5 [56]. In contrast, Ad5 neutralizing titers are highly prevalent worldwide and particularly in Africa and high levels of Ad5 neutralizing antibody responses are elicited post vaccination [12, 27, 29, 38–40]. In spite of pre-existing Ad5 neutralization titers, strong HIV-specific T cell and antibody responses are detected in the majority of volunteers enrolled in Ad5 trials. Likewise, for malaria Ad35 vaccine trials, robust T cell and antibody responses are detected in the majority of participants [55, 57]. Results from ongoing HIV vaccine trials also indicate that the presence of pre-existing Ad35 and Ad26 neutralizing antibodies does not impact HIV-specific T cell and antibody responses. Pre-existing Ad5 neutralizing antibodies and other immune responses have been associated with increased susceptibility to HIV-infection after vaccination with Ad5-gag, pol, nef [27, 29, 58–60].

It would have been valuable to compare the impressive systemic T cell responses with those of mucosal cells such as α4γ7+ expressing CD4+ T cells. However, this study did not obtain biopsy samples hence did not evaluate T cell subsets that are targeted by the HIV virus. Future studies with these vaccines should evaluate target T cell subsets in humans as described recently [10]. Another limitation of this study is that the total volume of blood was limited so the primary immunogenicity assays were prioritized hence no Adenovirus peptide pool was included in the functional screens.

Placebo recipients were included to provide an appropriate control group for evaluation of vaccine safety and tolerability, particularly local and systemic reactogenicity [61]. We felt this was particularly important for these prime boost regimens including an adjuvant, as adjuvants may have significant reactogenicity profiles. This trial was designed to evaluate immunogenicity responses in people without Ad35 pre-existing immunity, therefore it is difficult to comment on the issue of potential enhancement due to pre-existing Ad35 immunity. In subsequent trials (unpublished data) we have evaluated the Ad35-GRIN and other Ad35 vaccines in people with and without Ad35 pre-existing immunity.

Overall the combination of adjuvanted F4 and Ad35 GRIN induced balanced CD4+ and CD8+ T-cell responses with multifunctional profiles which persisted over time and high titers of F4 binding antibodies. The vaccine regimens assessed in this trial were designed to induce multifunctional CD4+ and CD8+ T-cell responses to deal with viral escape and inhibit viral activity across clades. Next generation immunogens may further improve the quality and breadth of T-cell responses across clades and should be incorporated into regimens such as those assessed in this trial: using low-seroprevalent adenoviral vectors and adjuvanted proteins in co-administration, ideally combined with Env immunogens with the capacity to induce neutralizing and/or non-neutralizing antibodies.

## Supporting Information

S1 CONSORT Checklist CONSORT Checklist. IAVI B002 study CONSORT checklist of information.
(DOC)

S1 Fig ELISPOT responses to HIV peptide pools. IFN-γ ELISpot responses to individual peptide pools 4 weeks after last vaccine (M5). The y-axis shows the SFC/10^6^ PBMC on a half-log scale. Panel A shows individual F4-specific responses across groups A-D: clade B p24, RT, Nef and p17 peptide pools and Panel B shows individual GRIN-specific responses across groups A-D: clade A Gag, RT, Int and Nef peptide pools. Gray dots: response below the cut-off to any of the 8 peptide pools; red circles: response above the cut-off to any of the 8 peptide pools. For the vaccine groups, the overlaid box plot summarizes the overall responses (i.e., the median, 1st and 3rd quartiles and 5^th^, 95^th^ Percentile). Baseline (BL) and placebo (Pbo) groups were combined for M5.
(DOCX)

S2 Fig Viral Inhibition Assay Results. VIA activity across groups B-D (vaccinees only). The mean log viral inhibition +/- standard deviation for IIIB and U455 viruses is shown at baseline (M0) for each of groups B-D and Group B; 4 weeks after two F4/AS01_B_ administrations (M2) and after Ad35-GRIN (M5), Group C; 4 weeks after Ad35-GRIN (M1) and 4 weeks after two F4/AS01_B_ administrations (M5) and Group D; 4 weeks after each co-administration of F4/AS01_B_ and Ad35-GRIN (M1, M2 and M5).
(DOCX)

S1 Protocol Trial Protocol. IAVI B002 Protocol V4.0. A Phase I double-blinded, placebo controlled, randomized trial in HIV-uninfected, healthy adult volunteers to evaluate the safety and immunogenicity of F4Co adjuvanted AS01B or AS01E administered with Ad35-GRIN.
(PDF)

S1 Table ELISpot responders. (DOCX)

S2 Table CD40L+ CD4+ CD8+ T cell responders. (DOCX)

S1 Text Supplementary Safety Results. Additional safety data.
(DOCX)
