# Supplementary material for: A Phase I Double Blind, Placebo-Controlled, Randomized Study of the Safety and Immunogenicity of an Adjuvanted HIV-1 Gag-Pol-Nef Fusion Protein and Adenovirus 35 Gag-RT-Int-Nef Vaccine in Healthy HIV-Uninfected African Adults
Source: PLoS One. 2015 May 11;10(5):e0125954. doi: 10.1371/journal.pone.0125954 (PMC4427332; doi:10.1371/journal.pone.0125954)
Supplement: S1 Fig — IFN-γ ELISpot responses to individual peptide pools 4 weeks after last vaccine (M5). The y-axis shows the SFC/106 PBMC on a half-log scale. Panel A shows individual F4-specific responses across groups A-D: clade B p24, RT, Nef and p17 peptide pools and Panel B shows individual GRIN-specific responses across groups A-D: clade A Gag, RT, Int and Nef peptide pools. Gray dots: response below the cut-off to any of the 8 peptide pools; red circles: response above the cut-off to any of the 8 peptide pools. For the vaccine groups, the overlaid box plot summarizes the overall responses (i.e., the median, 1st and 3rd quartiles and 5th, 95th Percentile). Baseline (BL) and placebo (Pbo) groups were combined for M5. (DOCX) [file pone.0125954.s002.docx]

**B. GRIN peptides**

1. **F4 peptides**

**Figure S1.** **IFN-γ ELISpot responses to individual peptide pools 4 weeks after last vaccine (M5).** The y-axis shows the SFC/10^6^ PBMC on a half-log scale. Panel A shows individual F4-specific responses across groups A-D: clade B p24, RT, Nef and p17 peptide pools and Panel B shows individual GRIN-specific responses across groups A-D: clade A Gag, RT, Int and Nef peptide pools. Gray dots: response below the cut-off to any of the 8 peptide pools; red circles: response above the cut-off to any of the 8 peptide pools. For the vaccine groups, the overlaid box plot summarizes the overall responses (i.e., the median, 1st and 3rd quartiles and 5^th^, 95^th^ Percentile). Baseline (BL) and placebo (Pbo) groups were combined for M5.
