# Supplementary material for: A Phase I Double Blind, Placebo-Controlled, Randomized Study of the Safety and Immunogenicity of an Adjuvanted HIV-1 Gag-Pol-Nef Fusion Protein and Adenovirus 35 Gag-RT-Int-Nef Vaccine in Healthy HIV-Uninfected African Adults
Source: PLoS One. 2015 May 11;10(5):e0125954. doi: 10.1371/journal.pone.0125954 (PMC4427332; doi:10.1371/journal.pone.0125954)
Supplement: S2 Fig — VIA activity across groups B-D (vaccinees only). The mean log viral inhibition +/- standard deviation for IIIB and U455 viruses is shown at baseline (M0) for each of groups B-D and Group B; 4 weeks after two F4/AS01B administrations (M2) and after Ad35-GRIN (M5), Group C; 4 weeks after Ad35-GRIN (M1) and 4 weeks after two F4/AS01B administrations (M5) and Group D; 4 weeks after each co-administration of F4/AS01B and Ad35-GRIN (M1, M2 and M5). (DOCX) [file pone.0125954.s003.docx]

**Figure S2.** **VIA activity across groups B-D (vaccinees only).** The mean log viral inhibition +/- standard deviation for IIIB and U455 viruses is shown at baseline (M0) for each of groups B-D and Group B; 4 weeks after two F4/AS01_B_ administrations (M2) and after Ad35-GRIN (M5), Group C; 4 weeks after Ad35-GRIN (M1) and 4 weeks after two F4/AS01_B_ administrations (M5) and Group D; 4 weeks after each co-administration of F4/AS01_B_ and Ad35-GRIN (M1, M2 and M5).
