# Supplementary material for: A Phase I Double Blind, Placebo-Controlled, Randomized Study of the Safety and Immunogenicity of an Adjuvanted HIV-1 Gag-Pol-Nef Fusion Protein and Adenovirus 35 Gag-RT-Int-Nef Vaccine in Healthy HIV-Uninfected African Adults
Source: PLoS One. 2015 May 11;10(5):e0125954. doi: 10.1371/journal.pone.0125954 (PMC4427332; doi:10.1371/journal.pone.0125954)
Supplement: S1 Protocol — IAVI B002 Protocol V4.0. A Phase I double-blinded, placebo controlled, randomized trial in HIV-uninfected, healthy adult volunteers to evaluate the safety and immunogenicity of F4Co adjuvanted AS01B or AS01E administered with Ad35-GRIN. (PDF) [file pone.0125954.s004.pdf]

TITLE
F4co / $AS01_B$ or $AS01_E$ and
Ad35-GRIN Multigenic HIV Vaccines Prime-Boost Trial

**Protocol Title:**	A Phase I double-blinded, placebo-controlled, randomized trial in HIV-uninfected, healthy adult volunteers to evaluate the safety and immunogenicity of F4co adjuvanted with $AS01_B$ or $AS01_E$ administered with Ad35-GRIN
**Protocol Number:**	IAVI B002
**Phase:**	Phase I
**IND Number:**	14401
**Sponsor:**	International AIDS Vaccine Initiative (IAVI)<br>110 William Street, 27th Floor<br>New York, New York 10038-3901<br>USA
**Sponsor Status:**	Non-Profit Organization
**Date of Protocol**	3 Aug 2010
**Version:**	4.0

The **CONFIDENTIAL INFORMATION** in this document is provided to you as an Investigator, potential Investigator, or Consultant, for review by you, your staff, and applicable institutional review boards (IRBs) and/or independent ethics committees (IECs). It is understood that the information will not be disclosed to others, except to the extent necessary to obtain ethical and regulatory approval from the respective committee's agencies and informed consent from those persons to whom the Investigational Product may be administered.

SYNOPSIS

TITLE: A Phase I double-blinded, placebo-controlled, randomized trial in HIV-uninfected, healthy adult volunteers to evaluate the safety and immunogenicity of F4co adjuvanted with $AS01_B$ or $AS01_E$ administered with Ad35-GRIN

PROTOCOL NUMBER: IAVI B002

PHASE: Phase I

SPONSOR: International AIDS Vaccine Initiative (IAVI)
110 William Street, 27th Floor
New York, New York 10038-3901
USA

SPONSOR STATUS: Non-Profit Organization

OBJECTIVES: ***Primary:***

Safety

- To evaluate the safety and tolerability of F4co formulated in $AS01_B$ or $AS01_E$ and Ad35-GRIN in the following regimen:
  - F4co formulated in $AS01_B$ or $AS01_E$ followed by Ad35-GRIN
  - Ad35-GRIN followed by F4co formulated in $AS01_B$
  - F4co formulated in $AS01_B$ co-administered (simultaneous administration with separate injections) with Ad35-GRIN

Immunogenicity

- To evaluate the immunogenicity of F4co formulated in $AS01_B$ or $AS01_E$
  - To demonstrate the non-inferiority of the immune response (in terms of the magnitude of the CD4+ CD40L+ T cells expressing at least IL-2) to F4co formulated in $AS01_E$ compared to F4co formulated in $AS01_B$ at 2 weeks post second dose (Groups A and B)

Secondary:

Immunogenicity

- To evaluate the immunogenicity of F4co formulated in $AS01_B$ or $AS01_E$ and Ad35-GRIN
  - To evaluate the immunogenicity of two doses of F4co formulated

in $AS01_B$ and $AS01_E$ followed by a single Ad35-GRIN boost (Groups A and B) at 4 weeks and one year post-boost.

- To evaluate the immunogenicity of a single Ad35-GRIN prime followed by two doses of F4co formulated in $AS01_B$ (Group C) at 4 weeks and one year post-second F4co.
- To evaluate the immunogenicity of 3 doses of co-administered F4co formulated in $AS01_B$ and Ad-35 GRIN (Group D) at 4 weeks and one year post-third dose.
- To evaluate the immunogenicity of two doses of F4co formulated in $AS01_B$ (Group B) or in $AS01_E$ (Group A) at 4 and 12 weeks post-second F4co, a single dose of Ad35-GRIN (Group C) at 4 and 12 weeks post–Ad35-GRIN, and two doses of co-administered F4co formulated in $AS01_B$ and Ad-35 GRIN (Group D) at 4 weeks post–first co-administration, 4 and 12 weeks post–second co-administration.

ENDPOINTS: ***Primary***:

Safety and tolerability:

- Proportion of volunteers with solicited adverse events (any OR any grade 3 or above) during a 14 day (Day 0 to Day 14) follow-up period after each vaccination

- Proportion of volunteers with unsolicited adverse events (any OR any grade 3 or above OR any related event OR any grade 3 or above and related) during a 4 week (Day 0 to Day 28) follow-up period after each vaccination and over the whole vaccination period

- Proportion of volunteers with abnormal biochemical and haematological values (any grade 3 or above) after each vaccination and throughout the study period

- Proportion of volunteers with serious adverse events (any event OR any related event) throughout the study period

Immunogenicity:

- To assess non-inferiority: magnitude of the CD4+ CD40L+ T cells expressing at least IL-2 to F4co formulated in $AS01_E$, and F4co formulated in $AS01_B$

Secondary:

Immunogenicity:

- Proportion of volunteers with HIV-1 specific T-cell responses measured by IFN-γ and IL-2 ELISPOT assay at indicated time points after stimulation with HIV-1 Clade A and B peptide pools matched to the vaccines

- Magnitude of HIV-1 specific T-cell responses measured by IFN-γ and IL-2 ELISPOT assay at indicated time points after stimulation with

HIV-1 Clade A and B peptide pools matched to the vaccines

- Proportion of volunteers with HIV-1 specific CD4+ or CD8+ T-cell responses assessed by Intracellular Cytokine Staining measuring IL-2 and / or IFN-γ and/or TNF-α at indicated time points after stimulation with HIV-1 Clade A and B peptide pools matched to the vaccines

- Magnitude of HIV-1 specific CD4+ or CD8+ T-cell responses assessed by Intracellular Cytokine Staining measuring IL-2 and / or IFN-γ and/or TNF-α at indicated time points after stimulation with HIV-1 Clade A and B peptide pools matched to the vaccines

- Seropositivity rates and magnitude of antibody titres to HIV-1 Clade B p17, p24, Nef, RT and F4co as measured by ELISA at indicated time points

- Seropositivity rates and magnitude of anti-Ad35 neutralizing antibody titres at indicated time points

Exploratory:

- Vaccine-specific T-cell response at any of the pre-determined time points to assess both frequency and magnitude of responses measured by:
    - T-cell proliferation frequency and magnitude
    - T-cell markers for memory, exhaustion and activation, secretion of cytokines other than IFN-γ, IL-2 and TNF-α
    - Viral inhibition assay and other possible functional assays
    - Vaccine-induced positive reactions measured by SELECTEST (antibody ELISA assay for HIV infection in the presence of vaccine-induced responses) as well as licensed HIV tests
    - Number of epitopes recognized by peripheral T cells in selected volunteers

- Anti-Ad35 cell-mediated immune responses at any of the indicated time points by ELISPOT or Intracellular Cytokine Staining (ICS)

- Proportion of volunteers and magnitude of response with HIV-1 specific responses (as measured by ELISPOT, ICS or other assays indicated above) at selected time points after stimulation with HIV-1 Clade C, D or other peptides sets

- Seropositivity rates and magnitude of antibody titres to HIV-1 non-Clade B antigens as measured by ELISA at indicated time points

- Functional antibodies, e.g. avidity or other functional antibody assays at any of the pre-determined time points

STUDY DESIGN TABLE

Group	N (vaccine/ placebo)	Months			
		0	1	3	4
**A**	28/7	F4co 10 μg/ $AS01_E$	F4co 10 μg/ $AS01_E$		Ad35-GRIN $2x10^{10}$ vp
**B**	28/7	F4co 10 μg/ $AS01_B$	F4co 10 μg/ $AS01_B$		Ad35-GRIN $2x10^{10}$ vp
**C**	28/7	Ad35-GRIN $2x10^{10}$ vp		F4co 10 μg/ $AS01_B$	F4co 10 μg/ $AS01_B$
**D**	28/7	F4co 10 μg/ $AS01_B$ Ad35-GRIN $2x10^{10}$ vp	F4co 10 μg/ $AS01_B$ Ad35-GRIN $2x10^{10}$ vp		F4co 10 μg/ $AS01_B$ Ad35-GRIN $2x10^{10}$ vp

vp: viral particles

METHODS: See Schedule of Procedures (Appendices A, B and C)

STUDY POPULATION: Healthy male or female adults 18 to 40 years of age, who are willing to undergo HIV testing, use an effective method of contraception, and who, in the opinion of the principal investigator or designee, understand the study and who provide written informed consent.

Principal exclusion criteria include confirmed HIV-1 or HIV-2 infection; detection of Ad35-specific serum neutralizing antibody; reported high-risk behaviour for HIV infection; pregnancy and lactation; chronic disease; clinically significant abnormal laboratory values, recent vaccination or receipt of a blood product or vaccine; and previous severe local or systemic reactions to vaccination or history of severe allergic reactions.

NUMBER OF VOLUNTEERS: Approximately 140 volunteers (112 vaccine / 28 placebo recipients) will be included in the study.

An over-enrolment of up to 7% (approximately 10 additional volunteers) will be permitted in the study to facilitate rapid enrolment.

DESCRIPTION OF INVESTIGATIONAL PRODUCT:

The study vaccines are as follows:

- F4co (Fusion protein p24-RT-Nef-p17 from HIV-1 subtype B Gag, Pol, Nef proteins) formulated in the adjuvants $AS01_B$ or $AS01_E$. Both adjuvant systems contain the immunostimulants Monophosphoryl Lipid A (MPL) and QS21 (50 µg of each for $AS01_B$ and 25 µg of each for $AS01_E$) formulated with liposomes

- Ad35-GRIN (recombinant, replication-incompetent adenovirus serotype35 expressing HIV-1 subtype A *gag*, *reverse transcriptase, integrase*, and *nef (GRIN)* genes)

- The associated placebo is*:* Saline (NaCl 0.9%)

Vaccine/ Placebo	Dosage Level	Volume in vial (mL)	Volume injected intramuscularly (mL)
F4co/$AS01_E$	10 µg	At least 0.5 mL	0.5 mL
F4co/$AS01_B$	10 µg	At least 0.5 mL	0.5 mL
Ad35-GRIN	$2x10^{10}$ vp	0.725 mL	1.0mL (use entire contents of one vial and sufficient amount from second vial to make 1.0 ml)
Saline	NA	0.7 mL	0.5 mL for F4co or 1.0 ml for Ad35-GRIN (using 2 vials)

BLINDING:

Volunteers will be randomly assigned to one of four groups described in the study design table above. Study staff (with the exception of the pharmacist) and volunteers will be blinded with respect to volunteer assignment between adjuvant groups A vs. B as well as to the allocation of active study vaccine or placebo within each group. There will be no blinding between group schedules A/B, C and D. Volunteers in Groups C or D will know their specific group assignment and will be blinded only with respect to the administration of vaccine or placebo.

DURATION OF STUDY PARTICIPATION:

Volunteers will be screened up to 42 days before vaccination (90 days for Ad35 neutralizing antibody screening) and will be followed for 12 months after the last vaccination.

It is anticipated that it will take approximately 5 months to enrol the study.

Upon completion of this study, volunteers will be asked to participate in the IAVI Long-Term Follow-Up study to assess safety (and immunogenicity if present at end of this study).

EVALUATION FOR INTERCURRENT HIV INFECTION:

Volunteers will be clinically evaluated and tested for HIV-1 and HIV-2 antibodies at time points specified in the Schedule of Procedures (Appendices A, B and C). Test results will be interpreted according to a pre-determined diagnostic algorithm. To distinguish a true HIV infection acquired through exposure in the community from the vaccine-induced antibody response, a nucleic-acid-based HIV test will be done. HIV testing at additional time points may be performed at the discretion of the volunteer and principal investigator or designee as medical or social circumstances arise.

To maintain blinding study staff with direct contact with the volunteers will be notified only if HIV infection is confirmed. Volunteers who acquire HIV infection during the study, despite counselling, will complete the Early Termination visit as detailed in the Schedule of Procedures and then be referred to IAVI Protocol H for follow-up of clinical, laboratory and immunology assessments.

STATISTICAL CONSIDERATIONS:

Collected data will be identified only by a volunteer identification number. Safety data will be reviewed by an independent Safety Review Board (SRB) for the first 28 volunteers in groups A/B, the first 14 volunteers in group C and the first 14 volunteers in group D at 2 weeks post month 1 and 2 weeks post month 4. Ad-hoc review may be specifically requested by the sponsor, GSK, the Principal Investigators or by the SRB.

At 1 month after the end of the vaccination regimen and at the end of the study, an analysis will be performed according to a pre-specified statistical analysis plan. The proportion of volunteers in each group who become HIV-infected will be analyzed (if any infections occur). Safety and tolerability will be addressed by examining the overall rates of adverse events and serious adverse events that might be associated with vaccination and the number of volunteers who experience these events. All clinical and routine laboratory data will be included in the safety analysis. Immunogenicity analyses will be performed in all volunteers who have received vaccine. Immune responses may be compared to results in the earlier IAVI-sponsored studies and earlier GSK studies.

TABLE OF CONTENTS

1.0. CONTACT INFORMATION 13

1.1 SPONSOR CONTACT INFORMATION 13
1.2 GSK COLLABORATING PARTNER CONTACT INFORMATION 14

2.0 SIGNATURE PAGE 16

3.0 INTRODUCTION AND BACKGROUND INFORMATION 17

3.1 STUDY RATIONALE 18
3.1.1 Experience with F4co protein adjuvanted with AS01 19
3.1.2 Experience with Ad35-GRIN 20

4.0 STUDY OBJECTIVES 21

4.1 PRIMARY OBJECTIVES 21
4.2 SECONDARY OBJECTIVES 22

5.0 STUDY ENDPOINTS AND STUDY DESIGN 22

5.1 STUDY ENDPOINTS 22
5.1.1 Primary Endpoints 22
5.1.2 Secondary Endpoints 23
5.1.3 Exploratory Endpoints 23
5.2 STUDY DESIGN 24
5.2.1 Duration of the Study 25
5.2.2 Study Population 25
5.2.3 Inclusion Criteria 25
5.2.4 Exclusion Criteria 26
5.2.5 Recruitment of Volunteers 28

6.0 STUDY VISITS 28

6.1 SCREENING PERIOD 28
6.2 VACCINATION VISIT 29
6.3 POST-VACCINATION VISITS 30
6.4 ADDITIONAL FOLLOW-UP VISITS 30
6.5 UNSCHEDULED VISITS/CONTACT 31
6.6 FINAL VISIT/EARLY TERMINATION VISIT 31

7.0 STUDY PROCEDURES 31

7.1 INFORMED CONSENT PROCESS 31
7.2 MEDICAL HISTORY AND PHYSICAL EXAMINATION 32
7.3 HIV RISK ASSESSMENT, HIV TESTING AND HIV-TEST COUNSELLING 32
7.4 HIV RISK REDUCTION COUNSELLING 33
7.5 FAMILY PLANNING COUNSELLING 33
7.6 SPECIMENS 33
7.7 REIMBURSEMENT 34
7.8 RANDOMIZATION AND BLINDING 34
7.9 UNBLINDING PROCEDURE FOR INDIVIDUAL VOLUNTEERS 34
7.10 REFERRAL TO LONG TERM FOLLOW-UP STUDY 34

8.0 INVESTIGATIONAL PRODUCT 35

8.1 DESCRIPTION 35
8.1.1 F4co/AS01 35
8.1.2 Ad35-GRIN 36
8.1.3 Placebo 36

8.2 SHIPMENT AND STORAGE 36
8.3 DISPENSING AND HANDLING 37
8.4 ADMINISTRATION 37
8.5 ACCOUNTABILITY AND DISPOSAL 37

9.0 ASSESSMENTS 37

9.1 SAFETY ASSESSMENTS 37
9.1.1 Local solicited adverse events 38
9.1.2 Systemic solicited adverse events....38
9.1.3 Vital Signs....38
9.1.4 Other adverse events....38
9.1.5 Concomitant Medications....39
9.1.6 Routine laboratory parameters....39
9.1.7 Specific screening tests:....40
9.2 IMMUNOGENICITY ASSESSMENTS....40
9.2.1 Antibody Responses 40
9.2.2 Cellular Responses 40
9.2.3 PBMC, Serum and Plasma Storage....41
9.3 OTHER ASSESSMENTS 41
9.3.1 HLA (Human Leukocyte Antigen) Typing....41
9.3.2 HIV test....41
9.3.3 Pregnancy Test 42
9.3.4 Ophthalmic Examination 42

10.0 ADVERSE EVENTS 42

10.1 DEFINITION....42
10.2 ASSESSMENT OF SEVERITY OF ADVERSE EVENTS 42
10.3 RELATIONSHIP TO INVESTIGATIONAL PRODUCT....43
10.4 SERIOUS ADVERSE EVENTS....43
10.5 CLINICAL MANAGEMENT OF ADVERSE EVENTS....44
10.6 PREGNANCY 45
10.7 INTERCURRENT HIV INFECTION....45
10.8 SERIOUS EVENT PRIOR TO INVESTIGATIONAL PRODUCT ADMINISTRATION 45

11.0 MANAGEMENT OF HIV ISSUES DURING AND FOLLOWING STUDY....45

11.1 HIV TESTING....45
11.2 SOCIAL DISCRIMINATION AS A RESULT OF AN ANTIBODY RESPONSE TO VACCINE 46
11.3 HIV INFECTION 46
11.3.1 Counselling....46
11.3.2 Referral for Support and/or Care 46

12.0 DISCONTINUATION OF VACCINATIONS AND/OR WITHDRAWAL FROM STUDY 47

12.1 DISCONTINUATION OF VACCINATIONS 47
12.2 FOLLOW UP AFTER DISCONTINUATION OF FURTHER VACCINATIONS 48
12.3 WITHDRAWAL FROM THE STUDY (EARLY TERMINATION)....48

13.0 DATA HANDLING 49

13.1 DATA COLLECTION AND RECORD KEEPING AT THE CLINICAL RESEARCH CENTRE....49
13.2 DATA COLLECTION AND TRANSFER AT THE HUMAN IMMUNOLOGY LABORATORY 49
13.3 DATA ENTRY AT THE CLINICAL RESEARCH CENTRE 49
13.4 DATA ANALYSIS....49

14.0 STATISTICAL CONSIDERATIONS 50

14.1 SAMPLE SIZE 50
14.2 STATISTICAL POWER AND ANALYSIS 50

14.2.1 Safety and Tolerability ... 50
14.2.2 Vaccine Immunogenicity ... 51

15.0 QUALITY CONTROL AND QUALITY ASSURANCE ... 53

16.0 DATA AND BIOLOGICAL MATERIAL ... 54

17.0 ADMINISTRATIVE STRUCTURE ... 54

17.1 PROTOCOL SAFETY REVIEW TEAM (PSRT) ... 54
17.2 SAFETY REVIEW BOARD (SRB) ... 54
17.2.1 Content of Interim Review ... 55
17.3 CRITERIA FOR PAUSING THE STUDY ... 55
17.4 STUDY SUPERVISION ... 55
17.5 STUDY MONITORING ... 56
17.6 INVESTIGATOR'S RECORDS ... 56

18.0 INDEMNITY ... 56

19.0 PUBLICATION ... 56

20.0 ETHICAL CONSIDERATIONS ... 57

APPENDIX A: SCHEDULE OF PROCEDURES—GROUPS A and B ... 58
APPENDIX B: SCHEDULE OF PROCEDURES—GROUP C ... 62
APPENDIX C: SCHEDULE OF PROCEDURES—GROUP D ... 66
APPENDIX D - ADVERSE EVENT SEVERITY ASSESSMENT TABLE ... 70

ABBREVIATIONS

Abbreviation	Term
AE	Adverse Event
AIDS	Acquired Immunodeficiency Syndrome
ALT	Alanine-Aminotransferase
AST	Aspartate-Aminotransferase
CFC	Cytokine Flow Cytometry
CLS	Contract Laboratory Services
CMI	Cell Mediated Immunity
CMO	Chief Medical Officer
CMV	Cytomegalovirus
CRC	Clinical Research Centre
CRF	Case Report Form
CTA	Clinical Trial Agreement
CTL	Cytotoxic T Lymphocyte
DCC	Data Coordinating Centre
DNA	Deoxyribonucleic Acid
ELISA	Enzyme Linked Immunosorbent Assay
ELISPOT	Enzyme Linked Immunospot Assay
GCP	Good Clinical Practice
GSK	GlaxoSmithKline Biologicals
HIV	Human Immunodeficiency Virus
HLA	Human Leukocyte Antigen
HSV	Herpes Simplex Virus
IAVI	International AIDS Vaccine Initiative
ICH	International Conference on Harmonization
ICS	Intracellular Cytokine Staining
IDES	Internet Data Entry System
IFN-γ	Interferon-gamma
IL-2	Interleukin-2
Kg	Kilogram
LIMS	Laboratory Information Management System
mg	Milligram
PCR	Polymerase Chain Reaction
pfu	Plaque Forming Units
PBMC	Peripheral Blood Mononuclear Cells
RPR	Rapid Plasma Reagin
SAE	Serious Adverse Event
SIV	Simian Immunodeficiency Virus
SHIV	Simian-Human Immunodeficiency Virus
SOP	Standard Operating Procedure
SOM	Study Operations Manual
SRSV	Small Round Structured Virus
SRB	Safety Review Board
STD	Sexually Transmitted Disease
TPHA	Treponema Pallidum Hemagglutination

Abbreviation	Term
TNF-α	Tumour-necrosis factor alpha
VDP	Vaccine Development Partnership
VIA	Viral Inhibition Assay
vp	Viral particle

1.0. CONTACT INFORMATION

1.1 Sponsor Contact Information

Trial Sponsor

International AIDS Vaccine Initiative (IAVI)<br>110 William Street, 27th Floor<br>New York, NY 10038-3901, USA	Telephone: + 1 212 847 1068<br>Fax: + 1 212 847 1113<br>Mobile: + 1 646 206 6611

Chief Medical Officer

Patricia Fast, MD, PhD<br>Chief Medical Officer, Medical Affairs<br>International AIDS Vaccine Initiative<br>110 William Street, 27th Floor<br>New York, NY 10038-3901, USA	Telephone: +1 212 847 1068<br>Fax: +1 212 847 1113<br>Mobile: +1 646 206 6611<br>E-mail: pfast@iavi.org

Medical Monitor

Frances Priddy, MD MPH<br>Senior Director, Medical Affairs<br>110 William Street, 27th Floor<br>New York, NY 10038-3901, USA	Telephone: + 1 608 203 5141<br>Fax: + 1 608 203 5501<br>Mobile: + 1 646 287 8943<br>E-mail: fpriddy@iavi.org

Clinical Program Manager

Marietta Krebs, MHSA, MBA<br>International AIDS Vaccine Initiative<br>50 Beale Street, Suite 1300<br>San Francisco, CA 94105	Telephone: +1 415 597 9147<br>Fax:<br>Mobile: +1 646 945 2926<br>Email: mkrebs@iavi.org

Data Coordinating Center

The EMMES Corporation<br>401 N. Washington Street<br>Suite 700<br>Rockville, MD 20850, USA	Telephone: + 1 301 251 1161<br>Fax: + 1 301 251 1355<br>Email: iavitrials@emmes.com

Trial Monitoring

Helen Thomson<br>Clinical Program Director, Africa<br>International AIDS Vaccine Initiative (IAVI)<br>East Africa Office<br>ABC Place (Building 2, 3rd Floor)<br>Waiyaki Way<br>P.O. Box 340-00202 KNH<br>Nairobi, Kenya	Telephone: +254 20 445 3596/7<br>Mobile: +254 720243795<br>Fax: +254 20 445 3598<br>E-mail: hthomson@iavi.org

Human Immunology Lab (HIL)

Jill Gilmour, Ph.D
Senior Director, Clinical Research
International AIDS Vaccine Initiative (IAVI)
5th Floor St. Stephen's Centre
Chelsea and Westminster Hospital
369 Fulham Road
London SW10 9NH United Kingdom

Telephone: + 44 208 746 5098
Fax: + 44 208 746 5096
Mobile: + 44 7810 202 699
E-mail: jgilmour@iavi.org

Josephine H Cox, Ph.D
Director Clinical Immunology
International AIDS Vaccine Initiative
1600 Gude Drive, Room 114
Rockville, MD 20850, USA

Telephone: +1 301 251 8360
Fax: +1 301 424 3120
Mobile: +1 646 639 5904
Email: jcox@iavi.org

Gwynn Stevens, Ph.D
Director Clinical Laboratory Program
International AIDS Vaccine Initiative
6 Albury Park, Dunkeld West
Johannesburg, South Africa

Telephone: +27 11 325 4737
Fax: +27 11 759 6210
Mobile: +27 83 415 0818
Email: gstevens@iavi.org

Tony Tarragona, Ph.D.
Lab Manager/Administrator
IAVI Human Immunology Lab
Imperial College London, Faculty of Medicine
St. Stephen's Centre, 5th Floor
Chelsea Campus
369 Fulham Road
London SW10 9NH, United Kingdom

Telephone: + 44 (0) 20 8846 6300
Fax: + 44 (0) 7940 510090
Mobile: +44 (0) 20 8846 6301
E-mail: t.tarragona@imperial.ac.uk

1.2 GSK Collaborating Partner Contact Information

GSK Medical Experts

Ludo Lavreys, MD, PhD, MSc
Director, Clinical Development
GSK Biologicals
Rue de l'Institut, 89
1330 Rixensart, Belgium

Telephone: +32 2 656 5928
Email: ludo.x.lavreys@gskbio.com

Julia Zima, MD, PhD
GSK Safety Physician
Biologicals Clinical and Safety
Pharmacovigilance
GSK Biologicals
Rue de l'Institut, 89
1330 Rixensart, Belgium

Telephone: +32 2 656 55 69
Email: julia.zima@gskbio.com
Mobile: +32 479 029 887

GSK Laboratory Contacts

Patricia Bourguignon
Clinical Read-out Project Manager
HIV Program
Research & Development
GSK Biologicals
Rue de l'Institut, 89
1330 Rixensart, Belgium

Telephone: + 32 2 656 89 33
Email: patricia.bourguignon@gskbio.com

2.0 SIGNATURE PAGE

The signatures below constitute the approval of this protocol and the appendices and provide the necessary assurances that this study will be conducted in compliance with the protocol, GCP and the applicable regulatory requirement(s).

Sponsor:

Signed: ______________________________ Date: ______________

Patricia Fast, MD, Ph.D.
Chief Medical Officer, Medical Affairs, IAVI

Principal Investigator:

Signed: ______________________________ Date: ______________

Name (please print): ______________________________

Name of institution (please print): ______________________________

3.0 INTRODUCTION AND BACKGROUND INFORMATION

According to the Joint United Nations Programme on HIV/AIDS and the World Health Organization, as of the end of 2008 approximately 33.4 million people were estimated to be living with HIV/AIDS, with 95% residing in the developing world. It is estimated that in 2008 alone, 2.7 million people were newly infected with HIV, there were approximately 7,400 new infections per day and 2 million people died of AIDS[1].

Sub-Saharan Africa remains the region most affected by the AIDS pandemic accounting for 67% of new infections in adults, a majority of which are women , and 91% of new infections in children and and 72% of all AIDS deaths recorded in 2008. It is estimated that 1.9 million people were newly infected with HIV in 2008, bringing to 22.4 million the total number of people living with HIV in the region.

There is an urgent need to strengthen existing prevention methods such as behavioural interventions, condom use, sexually transmitted disease treatment, harm reduction, male circumcision and explore/ensure access to new prevention strategies to control the epidemic and prevent new infections, including pre-exposure prophylaxis, HIV treatment, and preventive vaccines and topical microbicides. The development of a vaccine against HIV type-1 remains the best hope for controlling the HIV/AIDS pandemic[2].

The effort to develop an effective preventive vaccine against HIV-1 infection is challenged by the wide genetic diversity of HIV-1 among different isolates[3 4 5]. Genomic recombination between different HIV-1 populations frequently occurs. With the continuous evolution and mixing of genetic subtypes and the reality of recombination, new vaccine strategies capable of coping with the possibility of antigenic change will be needed. Group M viruses are the most common globally and are further subdivided into different genetic subtypes or clades. Analysis of genomic sequences from different regions in the world have identified ten clades to date, designated A--H, J and K and dozens of recombinant forms, but together, the A, B and C subtypes are responsible for about 75%-85% of new HIV infections in the world[6]. In Kenya, subtype A is the predominant subtype and a high proportion of intersubtype recombinants are seen[7 8] . In Uganda, subtypes A and D predominate and there are a smaller proportion of recombinants of subtypes A and D seen [9 10 11 12] . In Zambia, subtype C is the predominant strain seen (95%), although subtypes A, D, G and J have also been identified [13 14]. .

To be effective, an HIV vaccine will have to induce appropriate immune responses that are potent and long-lasting. The immune correlates for protection that may be required are not known, but experimental and natural history studies suggest that both high levels of HIV-specific neutralizing antibody and long-lasting effector and central memory HIV-specific CD8+ and CD4+ T-cell responses are needed in both systemic and mucosal compartments [15 16 17 18 19 20 21 22].

Neutralizing antibodies against circulating isolates are induced principally by the envelope glycoprotein of HIV (Env) and could confer sterilizing immunity against HIV as suggested by non-human primate SHIV challenge studies [23 24 25]. However, attempts to design appropriate immunogens have failed. This has been a major drawback for env-based HIV vaccines, since current immunogens afford only very narrow protection against HIV strains that are closely related to the vaccine antigen[26 27]. The search for an immunogen able to induce broad cross-protective neutralizing antibodies remains difficult and critical [28 29].

Evidence that CD8+ cytotoxic T lymphocytes (CTL) can control AIDS virus replication in the absence of antibodies has been demonstrated in HIV-infected subjects with a reduction in viremia in acute infection temporally associated with HIV-1-specific CTL[30 31 32]. The role of CTL was further suggested in the SIV macaque model [33 34 35 36].

Issues of quantity, quality and location impact the ability of CD8+ T cells to mediate protection from infection[37]. To protect from viruses which remain within the cells of the host and cause persistent infection, such as HIV, the immune system has evolved CD4+ and CD8+ T cells. Both T cell subtypes are recruited in tissues by dendritic cells sensitized through the Toll-like receptors to the presence of pathogens. CD4+ T cells provide help to naive CD8+ T cells so they can proliferate and acquire the ability to recognize foreign antigens presented on the surface of the infected cells and kill them. Naive CD8+ and CD4+ T cells become memory T cells once they have encountered the antigen. There are two main subtypes, effector and central memory CD8+ T cells that can be differentiated by their surface expression of receptors, cytokine production and ability to proliferate[38].

Preservation and/or restoration of intestinal CD4+ memory T cells seems to be associated with protection from challenge and control of viremia in the non-human primate SIV challenge model[39]. These results suggest that protection against pathogenic lentiviral infection or disease progression may correlate with preservation of mucosal CD4+ T cells. In animals, accumulating evidence suggests that HIV-specific CD4+ T cells are equally crucial for the induction of a protective immune response against HIV[40]. Furthermore, the analysis of immune responses in HIV-infected individuals suggests a crucial role for CD4+ T cells[41]. CD4+ T cells provide essential help to CD8+ effector T cells in long-term non-progressors [42 43 44]. In contrast, the absence of HIV-specific CD4+ T cells in chronically infected individuals seems to be related to an impairment of CD8+ T cell maturation[45].

Altogether, these data suggest that an effective HIV vaccine should induce strong HIV-specific CD4+ and CD8+ T cell responses.

The prime-boost strategy involves priming the immune system to a target antigen delivered by one vaccine and then selectively boosting this immunity by repeat administration of the antigen by a second and distinct vaccine[46]. The synergistic enhancement of immunity to the target antigen is reflected in an increased number of antigen-specific T cells, selective enrichment of high avidity T cells and increased efficacy against pathogenic challenge [47]. Heterologous HIV immunogens derived from different clades for sequential priming and boosting predominantly stimulated T-cell immunity against conserved epitopes, whereas a single vaccine derived from one clade or the mixture of multiple vaccines from different clades primarily raised T-cells against less conserved or non-conserved epitopes[48 49].

It is proposed to develop a sequential prime-boost or concomitant administration regimen to elicit both HIV-specific CD4+ T cells with adjuvanted protein vaccines and HIV-specific CD8+ T cells with a recombinant viral vector (adenovirus), the two vaccines acting possibly in synergy.

3.1 Study Rationale

This study is a Phase I randomized, placebo-controlled, double-blind clinical trial in HIV-uninfected, healthy adult volunteers to evaluate the safety and immunogenicity of F4co 10μg formulated in AS01 adjuvant ($AS01_B$ vs. $AS01_E$; $AS01_E$ contains half the quantity of immunostimulants of $AS01_B$) and Ad35-GRIN at $2x10^{10}$vp.

The two vaccine candidates will be used in two different prime-boost regimens and a co-administration regimen as follows:

- F4co formulated in $AS01_B$ or $AS01_E$ followed by Ad35-GRIN
- Ad35-GRIN followed by F4co formulated in $AS01_B$
- F4co formulated in $AS01_B$ co-administered with Ad35-GRIN

The trial design is hypothesis-driven:

- F4co formulated in $AS01_B$ and $AS01_E$ and Ad35-GRIN have acceptable safety profiles and induce robust cell-mediated immune responses in non-human primates and humans
- F4co formulated in $AS01_B$ and $AS01_E$ induces HIV-specific CD4+ T-cell responses while Ad35-GRIN induces HIV-specific CD8+ T-cell responses
- A sequential prime-boost regimen may induce both CD4+ and CD8+ T-cell responses either by additive or synergistic mechanisms. This may result in broad and polyfunctional immune responses to several proteins and epitopes, overcoming the HIV-1 subtype diversity, and confering a greater magnitude and longer duration of response.
- The co-administration regimen may synergize simultaneously rather than sequentially the CD4+/CD8+ T-cell interaction and generate earlier and stronger immune responses.

3.1.1 Experience with F4co protein adjuvanted with AS01

F4co is a recombinant fusion protein (p24, Reverse Transcriptase, Nef, p17 (*p24-RT-Nef-p17*)) from conserved HIV-1 subtype B antigens (*gag, pol, nef*) formulated with the adjuvant system AS01 (immunostimulants MPL and QS21 formulated in liposomes). The F4co fusion protein was designed to include a broad range of cross-reacting T-cell epitopes from *gag, pol* and *nef* proteins from HIV-1 Clade B.

AS01 (liposome-based formulation containing the immunostimulants 3D-MPL [3-deacylated monophosphoryl lipid A] and QS21 [a triterpene glycoside purified from the bark of *Quillaja saponaria*]) (50 µg of each for $AS01_B$ and 25 µg of each for $AS01_E$) was chosen among other potent adjuvants, from several clinical studies for its propensity to induce a stronger Th1 cellular-mediated immune response and its acceptable safety profile[50 51 52 53].

The first time in human study, PRO-HIV-005, investigated the safety and immunogenicity of GSK's novel recombinant protein HIV antigen F4co, adjuvanted or not, in an antigen dose-escalation design (10, 30 or 90 µg) in healthy, HIV-uninfected volunteers. The study was conducted in 180 volunteers at the Center for Vaccinology, Ghent University and Hospital (Belgium); 3 groups of 50 volunteers received two doses, at one month intervals, of F4co at 10, 30 or 90 µg adjuvanted with $AS01_B$, and 3 groups of 10 volunteers received non-adjuvanted F4co antigen (reconstituted in

water for injection) at the same dose levels and following the same schedule.

During the 7-day post-vaccination period, higher reactogenicity was observed in the adjuvanted study vaccine groups as compared to the non-adjuvanted study vaccine groups but reactogenicity was not related to antigen dose level. The F4co vaccine, either non-adjuvanted or adjuvanted with $AS01_B$, had an acceptable reactogenicity profile. No vaccine-related SAEs were reported.

Very potent, persistent and polyfunctional CD4+ T cell responses were observed in this study. Interestingly, the F4co 10 µg /$AS01_B$ dose induced the highest frequency of specific CD4+ T cells, with all subjects responding to at least three of the antigens and 80% responding to all four antigens. As a result, 10µg F4co/500µl $AS01_B$ was chosen as the optimal combination for further development. [54]

During the conduct of a preclinical toxicology study in minipigs evaluating a combination of the F4co/ $AS01_B$ vaccine candidate with a DNA vaccine, lens opacities in the eyes were noted. After a full review of the preclinical toxicology data, the findings remained inconclusive. Therefore, a second preclinical toxicology study was conducted using New Zealand White rabbits, which have a very low background incidence of lens opacity. No signs of treatment-related lens opacity were seen in the F4co/ $AS01_B$, $AS01_B$ or F4co groups. No treatment-related gross or microscopic findings were observed at ophthalmologic examinations.

See F4co Investigator Brochure for full description of the preclinical and clinical immunogenicity and safety profile of this candidate vaccine.

3.1.2 Experience with Ad35-GRIN

Ad35-GRIN is a replication-incompetent recombinant Adenovirus serotype 35 expressing HIV-1 Clade A genes (*gag, reverse transcriptase, integrase* and *nef* (GRIN). The GRIN genes were identified within the HIV-1 sequence, designed as a fusion product, and codon-optimized for human cell expression and translation. GRIN was selected based on the evidence that in a worldwide study assessing HIV-1-infected humans, the highest levels of T-cell responses (75-100%) were observed against gag, pol, and nef, regardless of either the donor’s origin or the subtype of the infecting virus [55]. Where appropriate, mutations have been introduced to abrogate the normal functions of the HIV proteins.

The recombinant adenovirus (rAd) vaccine design is based on the concept of immunisation by gene transfer. Following entry into target cells, it is expected that the HIV-1 gene products will be expressed without the production of infectious adenovirus particles or integration into the host genome and stimulate predominantly CD8+ cytotoxic T cells to the HIV-1 proteins. Preclinical studies[56 57 58 59 60] and clinical studies [61 62 63 64 65] show that immune responses against HIV can be elicited by direct gene transfer of immunogen-expressing HIV genes via recombinant

adenovirus vectors. The major advantage of rAd immunization appears to be its efficacy in transducing host cells and priming the induction of $CD8^+$ cytotoxic T lymphocytes (CTL) responses, which are considered important in limiting viral replication[66 67 68 69]. There is an additional safety feature in that following entry into the target cells, the HIV-1 gene products will be expressed without the production of infectious adenovirus (Ad) or integration into the host genome. These gene products can be produced in cells that are not actively dividing.

A second vector has been produced which contains the HIV-1 subtype A gp140 gene (Ad35-ENV), although it will not be used in this trial. Non-human primate immunogenicity data suggest that stronger HIV-specific CD8+ T cell responses are generated by Ad35-GRIN/ENV at a dosage of 2×10^{10} vp and 2×10^{11} than at lower dosage levels.

A Phase I placebo-controlled, double-blinded (in terms of vaccine or placebo), randomized dose-escalation trial to evaluate the safety and immunogenicity of Ad35-GRIN/ENV HIV Vaccine in 42 healthy adult volunteers is ongoing in the US (IAVI B001 – BB-IND# 13876). Three vaccine dosages are tested: 2×10^9, 2×10^{10}, and 2×10^{11} viral particles (vp). The vaccine is administered by intramuscular route at months 0 and 6. As of 17 December 2009, safety data is available for the first 42 injections of vaccine or placebo in all dose groups, with 13 and 8 receiving the 6-month booster dose in the 2×10^9 and 2×10^{10} groups respectively. Overall, the Ad35-GRIN/ENV vaccine was generally safe and well tolerated. The high dose of Ad35-GRIN/ENV at $2x10^{11}$ vp appeared more reactogenic both systemically and locally compared to the low ($2x10^9$ vp) and mid ($2x10^{10}$ vp) dose groups, with 5/14 volunteers in the high dose group reporting severe, but transient, systemic reactions after the first vaccination. No serious adverse event has been reported in either group. No Ad35 shedding was reported in the few samples tested to date. These preliminary data suggest that the $2x10^{11}$ vp dose may be likely to cause transient but moderate-severe systemic reactions.Therefore the $2x10^{10}$ vp dose was selected for this study.

See Ad35-GRIN/ENV Investigator Brochure for full description of the preclinical and clinical safety profile of this candidate vaccine.

4.0 STUDY OBJECTIVES

4.1 Primary Objectives

Safety

- To evaluate the safety and tolerability of F4co formulated in $AS01_B$ or $AS01_E$ and Ad35-GRIN in the following regimen:
  - F4co formulated in $AS01_B$ or $AS01_E$ followed by Ad35-GRIN
  - Ad35-GRIN followed by F4co formulated in $AS01_B$

- o F4co formulated in $AS01_B$ co-administered (simultaneous administration with separate injections) with Ad35-GRIN

Immunogenicity

- To evaluate the immunogenicity of F4co formulated in $AS01_B$ or $AS01_E$
  - o To demonstrate the non-inferiority of the immune response (in terms of the magnitude of the CD4+ CD40L+ T cells expressing at least IL-2) to F4co formulated in $AS01_E$ compared to F4co formulated in $AS01_B$ at 2 weeks post second dose (Groups A and B)

4.2 Secondary Objectives

Immunogenicity

- To evaluate the immunogenicity of F4co formulated in $AS01_B$ or $AS01_E$ and Ad35-GRIN
  - o To evaluate the immunogenicity of two doses of F4co formulated in $AS01_B$ and $AS01_E$ followed by a single Ad35-GRIN boost (Groups A and B) at 4 weeks and one year post-boost.
  - o To evaluate the immunogenicity of a single Ad35-GRIN prime followed by two doses of F4co formulated in $AS01_B$ (Group C) at 4 weeks and one year post-second F4co.
  - o To evaluate the immunogenicity of 3 doses of co-administered F4co formulated in $AS01_B$ and Ad-35 GRIN (Group D) at 4 weeks and one year post-third dose.
  - o To evaluate the immunogenicity of two doses of F4co formulated in $AS01_B$ (Group B) or in $AS01_E$ (Group A) at 4 and 12 weeks post-second F4co, a single dose of Ad35-GRIN (Group C) at 4 and 12 weeks post–Ad35-GRIN, and two doses of co-administered F4co formulated in $AS01_B$ and Ad-35 GRIN (Group D) at 4 weeks post–first co-administration, 4 and 12 weeks post–second co-administration.

5.0 STUDY ENDPOINTS AND STUDY DESIGN

5.1 Study Endpoints

5.1.1 Primary Endpoints

Safety and tolerability:

- Proportion of volunteers with solicited adverse events (any OR any grade 3 or above) during a 14 day (Day 0 to Day 14) follow-up period after each vaccination

- Proportion of volunteers with unsolicited adverse events (any OR any grade 3 or above OR any related event OR any grade 3 or above and related) during a 4 week (Day 0 to Day 28) follow-up period after each vaccination and over the whole vaccination period

- Proportion of volunteers with abnormal biochemical and haematological values (any grade 3 or above) after each vaccination and throughout the study period

- Proportion of volunteers with serious adverse events (any event OR any related event) throughout the study period

Immunogenicity:

- To assess non-inferiority: magnitude of the CD4+ CD40L+ T cells expressing at least IL-2 to F4co formulated in $AS01_E$, and F4co formulated in $AS01_B$

5.1.2 Secondary Endpoints

Immunogenicity:

- Proportion of volunteers with HIV-1 specific T-cell responses measured by IFN- γ and IL-2 ELISPOT assay at indicated time points after stimulation with HIV-1 Clade A and B peptide pools matched to the vaccines

- Magnitude of HIV-1 specific T-cell responses measured by IFN- γ and IL-2 ELISPOT assay at indicated time points after stimulation with HIV-1 Clade A and B peptide pools matched to the vaccines

- Proportion of volunteers with HIV-1 specific CD4+ or CD8+ T-cell responses assessed by Intracellular Cytokine Staining measuring IL-2 and / or IFN- γ and/or TNF-α at indicated time points after stimulation with HIV-1 Clade A and B peptide pools matched to the vaccines

- Magnitude of HIV-1 specific CD4+ or CD8+ T-cell responses assessed by Intracellular Cytokine Staining measuring IL-2 and / or IFN- γ and/or TNF-α at indicated time points after stimulation with HIV-1 Clade A and B peptide pools matched to the vaccines

- Seropositivity rates and magnitude of antibody titres to HIV-1 Clade B p17, p24, Nef, RT and F4co as measured by ELISA at indicated time points

- Seropositivity rates and magnitude of anti-Ad35 neutralizing antibody titres at indicated time points

5.1.3 Exploratory Endpoints

- Vaccine-specific T-cell response at any of the pre-determined time points to assess both frequency and magnitude of responses measured by:
  - T-cell proliferation frequency and magnitude
  - T-cell markers for memory, exhaustion and activation, secretion of cytokines other than IFN- γ, IL-2 and TNF-α
  - Viral inhibition assay and other possible functional assays

- o Vaccine-induced positive reactions measured by SELECTEST (antibody ELISA assay for HIV infection in the presence of vaccine-induced responses) as well as licensed HIV tests
- o Number of epitopes recognized by peripheral T cells in selected volunteers

- Anti-Ad35 cell-mediated immune responses at any of the indicated time points by ELISPOT or Intracellular Cytokine Staining (ICS)

- Proportion of volunteers and magnitude of response with HIV-1 specific responses (as measured by ELISPOT, ICS or other assays indicated above) at selected time points after stimulation with HIV-1 Clade C, D or other peptides sets

- Seropositivity rates and magnitude of antibody titres to HIV-1 non-Clade B antigens as measured by ELISA at indicated time points

- Functional antibodies, e.g. avidity or other functional antibody assays at any of the pre-determined time points

5.2 Study Design

The study is Phase 1, randomized, placebo-controlled clinical trial designed to evaluate the safety and immunogenicity of F4co formulated in $AS01_B$ or $AS01_E$ and Ad35-GRIN. Vaccine or placebo will be administered at 3 separate time points depending on the volunteer's group assignment.

Table 1
Study Design

Group	N (vaccine/ placebo)	Months			
		0	1	3	4
A	28/7	F4co 10 μg/ $AS01_E$	F4co 10 μg/ $AS01_E$		Ad35-GRIN $2x10^{10}$ vp
B	28/7	F4co 10 μg/ $AS01_B$	F4co 10 μg/ $AS01_B$		Ad35-GRIN $2x10^{10}$ vp
C	28/7	Ad35-GRIN $2x10^{10}$ vp		F4co 10 μg/ $AS01_B$	F4co 10 μg/ $AS01_B$
D	28/7	F4co 10 μg/ $AS01_B$ Ad35-GRIN $2x10^{10}$ vp	F4co 10 μg/ $AS01_B$ Ad35-GRIN $2x10^{10}$ vp		F4co 10 μg/ $AS01_B$ Ad35-GRIN $2x10^{10}$ vp

5.2.1 Duration of the Study

Volunteers will be screened up to 42 days before vaccination (up to 90 days for Ad35 neutralizing antibody) and will be followed for 12 months after the last vaccination (16 months total participation).

It is estimated that it will take approximately 5 months to complete enrolment.

5.2.2 Study Population

The study population consists of healthy male or female adults aged 18 to 40 years who are willing to undergo HIV testing, use an effective method of contraception, and who in the opinion of the investigator or designee, understand the study and provide written informed consent.

Approximately 140 volunteers (112 vaccine /28 placebo recipients) will be included in the study. An over-enrolment of up to 7% (approximately 10 additional volunteers) will be permitted in the study to facilitate rapid enrolment.

5.2.3 Inclusion Criteria

1. Healthy males and females, as assessed by a medical history, physical exam, and laboratory tests

2. At least 18 years of age on the day of screening and has not reached his/her 41st birthday on the day of first vaccination

3. Willing to comply with the requirements of the protocol and available for follow-up for the planned duration of the study (screening plus 16 months)

4. In the opinion of the Principal Investigator or designee and based on Assessment of Informed Consent Understanding results, has understood the information provided and potential risks linked to vaccination and participation in the trial. Written informed consent needs to be provided by the volunteer before any study-related procedures are performed

5. Willing to undergo HIV testing, risk reduction counselling, receive HIV test results and committed to maintaining low risk behaviour for the trial duration

6. If a female of childbearing potential (not menopausal or anatomically sterile), willing to use an effective non-barrier method of contraception (hormonal contraceptive; intra-uterine device), from screening until four months after last vaccination. All female volunteers must be willing to undergo urine pregnancy tests at time points as indicated in the Schedule of Procedures (Appendices A, B and C) and must test negative prior to each vaccination.

7. If sexually active male (who is not anatomically sterile), willing to use an effective method of contraception (such as consistent condom use) from the day of first vaccination until 4 months after the last vaccination

8. Willing to forgo donations of blood or any other tissues during the study and, for those who test HIV positive after vaccination, until the anti-HIV antibody titres become undetectable

5.2.4 Exclusion Criteria

1. Confirmed HIV-1 or HIV-2 infection

2. Any clinically relevant abnormality on history or examination including history of immunodeficiency, malignancy or autoimmune disease; use of systemic corticosteroids (<2 weeks use of topical or inhaled steroids is permitted); immunosuppressive, anticancer, anti-tuberculosis or other medications considered significant by the investigator within the previous 6 months

3. Any clinically significant acute or chronic medical condition that is considered progressive, or in the opinion of the investigator, makes the volunteer unsuitable for participation in the study

4. Detection of Ad35-specific serum neutralizing antibody

5. Reported high-risk behaviour for HIV infection within 6 months prior to first vaccination, as defined by:
   - Unprotected sexual intercourse with a known HIV-infected person, a partner known to be at high risk of HIV infection or a casual partner (i.e. no continuing established relationship)
   - Engaged in sex work for money or drugs
   - Frequent excessive alcohol use, binge drinking (e.g. men consume 5 or more and women 4 or more drinks in about 2 hours) or use of illicit drugs
   - Recently acquired a sexually transmitted disease (STD)
   - A high-risk partner currently or within the previous 6 months
   - Three or more sexual partners

   Note: Volunteers with the following behaviour within 6 months prior to first vaccination are eligible:
   - Two or fewer mutually monogamous relationships with HIV negative partners who do not use illicit drugs
   - Two or fewer partners who were HIV negative, did not use illicit drugs and with whom the volunteer used condoms for sexual intercourse

6. If female, pregnant or planning a pregnancy within 4 months after last vaccination; or lactating

7. Unstable asthma (e.g. sudden acute attacks occurring without an obvious trigger) or asthma requiring:
    - Daily steroid or long acting β agonist prevention
    - Hospitalization in the last two years

8. Bleeding disorder that was diagnosed by a physician e.g., factor deficiency, coagulopathy or platelet disorder that requires special precautions. (A volunteer who states that he or she has easy bruising or bleeding, but does not have a formal diagnosis and has intramuscular (IM) injections and blood draws without any adverse experience is eligible)

9. History of splenectomy

10. Any of the following abnormal laboratory parameters listed below (laboratory parameters must be consistent with I/E criteria prior to each vaccination):
    i. Hematology
        a. Hemoglobin <10.0 g/dL
        b. Absolute Neutrophil Count (ANC): ≤ 1000/mm^3
        c. Absolute Lymphocyte Count (ALC): ≤ 500/mm^3
        d. Platelets: ≤ 90,000/ mm^3
    ii. Chemistry
        a. Creatinine: > 1. 1 x ULN
        b. AST: >1.25 x ULN
        c. ALT: >1.25 x ULN
    iii. Urinalysis: abnormal dipstick confirmed by microscopy
        a. Protein 2+ or more
        b. Blood 2+ or more (not due to menses)
        c. Leukocytes 2+ or more

11. Receipt of live-attenuated vaccine within the previous 60 days (live attenuated flu vaccine within 14 days) or planned receipt within 60 days after vaccination with Investigational Product; or receipt of other vaccine (e.g. pneumococcal), allergy treatment with antigen injections or tuberculin skin test within the previous 14 days or planned receipt within 14 days after vaccination with Investigational Product

12. Receipt of blood transfusion or blood-derived products within the previous 6 months

13. Participation in another clinical trial of an Investigational Product currently, within the previous 3 months or expected participation during this study

14. Prior receipt of another investigational HIV vaccine candidate (note: receipt of an HIV vaccine placebo will not exclude a volunteer from participation if documentation is available and the IAVI Medical Monitor gives approval)

15. History of severe local or systemic reactogenicity to vaccines (e.g.

anaphylaxis, respiratory difficulty, angioedema)

16. History of severe allergic reactions to any substance requiring hospitalization or emergency medical care (e.g. Steven-Johnson syndrome, bronchospasm or hypotension)

17. Known sensitivity to sulphite, aspirin or aminoglycoside antibiotics (e.g. amikacin, arbekacin, gentamicin, kanamycin, neomycin, netilmicin, paromomycin, streptomycin, tobramycin, etc)

18. Confirmed diagnosis of active hepatitis B (HBsAg), hepatitis C (HCV antibodies), active syphilis or active tuberculosis

19. History of severe neurological disorder, seizure or psychiatric disorder (e.g schizophrenia, severe psychosis, bipolar disorder requiring therapy, suicidal attempt or ideation)

20. For those volunteers at clinical centres participating in ophthalmic examinations, any clinically significant abnormality found on baseline ophthalmic examination.

5.2.5 Recruitment of Volunteers

Healthy adult male and female volunteers may be recruited through information presented in community organizations, hospitals, colleges, other institutions and/or advertisements to the general public. This information will contain contact details. Low risk volunteers may also be recruited from epidemiology studies conducted at the research centres. If other recruitment strategies are used, the sponsor needs to be informed.

6.0 STUDY VISITS

6.1 Screening Period

During Screening, study staff will perform the following procedures:

- Provide and/or review the Informed Consent Document and answer any questions about the study prior to obtaining written informed consent
- Perform Assessment of Informed Consent Understanding with the volunteer
- Obtain written informed consent prior to conducting any study procedures
- Assign volunteer identification number

Volunteers will be screened first for Ad35-specific serum neutralizing antibodies within 90 days prior to the date of first vaccination. The first 10 volunteers screened at each research centre will also have PBMCs collected for QA purposes during the 90 day period. All other screening procedures must occur within 42 days prior to the date of first vaccination. Sample collection and procedures may be conducted at more than one screening visit, if required. If screening procedures are not performed within these time periods, however, they must be repeated. In that case, the complete medical history may be replaced by

an interim medical history and the Volunteer Information Sheet of the Informed Consent Document should be reviewed.

If the volunteer agrees to participate, study staff will:

- Administer screening questionnaire
- Assess HIV risk as part of screening questionnaire
- Conduct HIV risk reduction counselling
- Conduct pre-HIV test counselling
- Conduct Family Planning counselling and refer for contraceptive counselling if necessary
- Collect a comprehensive medical history
- Collect concomitant medication information
- Record height and weight
- Perform a general physical examination, which includes an examination of skin, respiratory, cardiovascular and abdominal systems
- Assess cervical and axillary lymph nodes
- Record vital signs (pulse, respiratory rate, blood pressure and temperature)
- Perform bilateral ophthalmic examination (only for specified centres; to be performed prior to first vaccination)
- Collect specimens for all tests as indicated in the Schedule of Procedures (Appendices A, B and C)

When available, the screening laboratory tests will be reviewed by the trial physician. Screening laboratory test(s) may be repeated once at the discretion of the principal investigator or designee to investigate any isolated abnormalities.

If a volunteer has signed the consent form but does not meet the eligibility criteria, the records must be kept at the CRC.

6.2 Vaccination Visit

Prior to the vaccination, study staff will:

- Answer any questions about the study
- Review the Informed Consent Document with volunteers
- Review screening safety laboratory data
- Conduct HIV risk assessment
- Conduct HIV risk reduction counselling
- Conduct pre- and post-HIV test counselling
- Conduct family planning counselling
- Review interim medical history
- Collect concomitant medication information
- Perform a symptom-directed physical examination and any further examination indicated by history or observation
- Record vital signs (pulse, respiratory rate, blood pressure and temperature)
- Perform baseline local and systemic reactogenicity assessment of the site of vaccination
- Collect specimens for all tests as indicated in the Schedule of Procedures (Appendices A, B and C). Obtain pregnancy test results prior to vaccination.

The volunteer will be assigned an allocation number according to the instructions specified in the Study Operations Manual.

The Investigational Product will be administered as specified in Section 8.4, Administration of Investigational Product.

For the first 20 volunteers enrolled and vaccinated, study staff will closely observe volunteers for at least 4 hours after first vaccination for any acute reactogenicity. For the remaining volunteers, study staff will closely observe volunteers for at least 30 minutes after vaccination. At the end of the observation period study staff will:

- Record vital signs (pulse, respiratory rate, blood pressure and temperature)
- Assess any local and systemic reactogenicity
- Assess any other adverse events.

For subsequent vaccination visits, study staff will perform the same procedures as above with the following exceptions:

- Review the routine safety laboratory parameters (9.1.6) from the previous visit prior to each vaccination. If a volunteer has an abnormal laboratory value that is known at the time of vaccination, follow the specified guidelines (Section 12.0)
- Conduct pre HIV-test counselling if an HIV Test is required (Appendices A, B and C) or provide post-test counselling if the results of a prior HIV test are being provided to the volunteer.
- All volunteers will be observed for at least 30 minutes after vaccination

6.3 Post-Vaccination Visits

The volunteer will be asked to return to the clinic on Day 3 (+/- 1 day) Day 7 (+/- 2 days), and Day 14 (+/- 3 days) after each vaccination for an assessment. The study staff will review the memory card with the volunteer and record the information in the clinic chart.

The following procedures will be conducted at this visit:

- Collect concomitant medication information
- If symptoms are present, perform a symptom-directed physical examination.
- Assess adverse events.
- Assess local and systemic reactogenicity
- Collect specimens for all tests as indicated in the Schedule of Procedures (Appendices A, B and C).

6.4 Additional Follow-up Visits

Assessments and procedures will be performed according to the Schedule of Procedures (Appendices A, B and C).

6.5 Unscheduled Visits/Contact

Unscheduled Visits/contacts are visits/contacts which are not described in the Schedule of Procedures (Appendices A, B and C). They may be performed at any time during the study. Unscheduled visits may occur:

- For administrative reasons, e.g., the volunteer may have questions for study staff or may need to re-schedule a follow-up visit.
- To obtain laboratory test results from a previous visit.
- For other reasons as requested by the volunteer or investigator.

All unscheduled visits will be documented in the volunteers' study records and on applicable source documents.

6.6 Final Visit/Early Termination Visit

Assessments and procedures will be performed according to the Schedule of Procedures (Appendices A, B and C). Volunteers will be offered participation in the IAVI Long-Term Follow-Up protocol.

7.0 STUDY PROCEDURES

7.1 Informed Consent Process

A sample Informed Consent Document consisting of a Volunteer Information Sheet and a Consent Form is provided by the sponsor to the CRC. This document needs to be made site-specific and translated (if necessary), submitted and approved by the IEC/ERB before it can be used at the CRC.

<u>Volunteer Information Sheet</u>
A qualified member of the study staff will obtain informed consent by reviewing the Volunteer Information Sheet.

The following study specific elements are included:

1. That it is unknown whether or not the vaccine(s) will protect against HIV infection or disease

2. That it may be possible that the vaccinated volunteer will develop antibodies against HIV following vaccination, which may produce a positive result in a routine HIV Antibody Test, and that provisions have been made to distinguish between response to vaccine and HIV infection during and after the study. The positive result may return to negative after some time or it may remain positive for a long period of time, possibly several years. In case the volunteer has a positive result in a routine HIV Antibody Test, he/she will be followed after the study is over.

3. That volunteers of childbearing potential should use a reliable form of contraception from screening throughout the vaccination period and until 4 months after the last vaccination
4. That placebo will be administered in this study and volunteer may receive placebo throughout the vaccination period

5. Potential risks and side effects of vaccination

Consent Form

Volunteers who give consent to participate in the study will do so on the basis of appropriate information and with adequate time to consider this information and ask questions. To confirm that the volunteer has understood the information contained in the Volunteer Information Sheet, an Assessment of Informed Consent Understanding (AOU) will be administered.

The volunteer's consent to participate must be obtained by him/her signing or marking, and dating 2 copies of the informed consent form. The person obtaining the consent will also sign. If the volunteer is functionally illiterate, the complete Informed Consent Document (including the Volunteer information Sheet) must be read to them in the language that they best understand in the presence of an independent literate observer not affiliated with the study, who will also sign and date the consent form as an impartial witness.

One copy of the signed/marked and dated Informed Consent Document must remain at the CRC. The second copy of the signed and dated Informed Consent Document will be offered to the volunteer to take home. Those volunteers who do not wish to take a copy will be required to document that they declined to do so.

Family members, sexual partner(s) or spouse(s) will be offered education and counselling regarding a volunteer's participation in the study ONLY if the participating volunteer has given their written consent.

7.2 Medical History and Physical Examination

At screening, a comprehensive medical history will be collected including details of any previous vaccinations and reaction to vaccinations, history of sexually transmitted diseases and contraceptive practices. At future visits, an interim medical history since the previous visit will be collected.

A general physical examination includes an examination of skin, respiratory, cardiovascular and abdominal systems and an assessment of cervical and axillary lymph nodes.

A symptom-directed physical examination includes any further examination indicated by history or observation.

Measurement of height and weight and the recording of vital signs (pulse, respiratory rate, blood pressure and temperature) will be performed at time points according to the Schedule of Procedures (Appendices A, B and C).

7.3 HIV Risk Assessment, HIV Testing and HIV-test Counselling

At screening, study staff will assess volunteers for past and current risk of HIV infection to determine eligibility for the study. In addition, HIV risk assessment will be performed at time points according to the Schedule of Procedure

(Appendices A, B and C) to measure any changes in HIV risk behaviour that may occur during the course of the study.

Additionally, study staff will perform pre-HIV test counselling (prior to collecting blood for an HIV test) and post-HIV test counselling (when HIV test results are available) at time points according to the Schedule of Procedures (Appendices A, B and C). For more information on HIV testing and HIV-test counselling, see Section 11.

7.4 HIV Risk Reduction Counselling

Study staff will provide individual HIV risk reduction counselling, including free condoms, at every visit. The procedures for risk reduction counselling will be detailed in site-specific SOPs.

7.5 Family Planning Counselling

Study staff will counsel volunteers about the importance of preventing pregnancies and use of condoms as well as other effective family planning methods. Condoms will be provided. Volunteers will be referred for family planning services either on-site or at a family planning clinic if a contraceptive prescription is required.

The family planning counselling will be performed at time points according to the Schedule of Procedures (Appendices A, B and C).

7.6 Specimens

For the first 10 volunteers screened at each centre, up to 86 mL of blood will be collected at the Screening Visit. 36mL is required for screening and eligibility tests, while the remaining 50mL is needed to evaluate PBMC collection and processing protocols for this study. For the remaining volunteers, up to 36 mL of blood will be collected at the Screening Visit. Up to 110 mL of blood will be collected at each later visit, usually from the antecubital fossa, according to the Schedule of Procedures (Appendices A, B and C).

All safety tests and HIV diagnostic tests will be performed at the CRC laboratories with the exception of PCR for confirmatory HIV testing which will be done by Contract Laboratory Services (CLS), South Africa. The Ad35 neutralization antibody assay will be conducted at the Kenya AIDS Vaccine Initiative (KAVI) Laboratory, Nairobi, or the IAVI Human Immunology Laboratory. All other immunology testing will be conducted at the CRCs, IAVI Human Immunology Laboratory and GSK laboratory, according to the Schedule of Procedures (Appendices A, B and C) and as indicated in section 9.2. Details of the assays and procedures will be described in the Laboratory Analytical Plan.

All specimens will be handled according to the procedures specified in the Study Operations Manual (SOM) and Laboratory Analytical Plan (LAP).

In the event of an abnormal laboratory value, volunteers may be asked to have an additional sample collected at the discretion of the principal investigator or designee.

7.7 Reimbursement

Volunteers will be reimbursed for their time, effort and for costs to cover their travel expenses to the CRC and inconvenience caused due to study participation. Reimbursement will be made after the completion of each study visit. Site-specific reimbursement amounts will be documented in the site-specific Volunteer Information Sheet approved by the Ethics Committee.

7.8 Randomization and Blinding

Volunteers will be identified by a unique volunteer identification number.

The randomization schedule will be prepared by the statisticians at the Data Coordinating Centre (DCC) prior to the start of the study. Volunteers will be automatically assigned a specific allocation number. An unblinding list will be provided to the CRC by the DCC. Volunteers will be randomly assigned to one of four groups (A-D).

This study is a double-blinded clinical trial. For all groups, study staff (with the exception of the pharmacist) and volunteers will be blinded to the allocation of active vaccine or placebo within each group. In Groups A and B, study staff (with the exception of the pharmacist) and volunteers will be blinded with respect to volunteer assignment between adjuvant groups A vs. B. There will be no blinding between group schedules A/B, C and D. Volunteers in Groups C or D will know their specific group assignment and will only be blinded with respect to receipt of vaccine or placebo.

Volunteers will be informed about their group assignment once the database is locked. If the study volunteer is unblinded and becomes aware of treatment group assignment during the study, further administration of the Investigational Product (vaccine or placebo) will be discontinued. The study volunteer will be followed up until the end of the study according to Schedule of Procedures (Appendices A, B and C).

7.9 Unblinding Procedure for Individual Volunteers

Unblinding of an individual volunteer is indicated only in the event of a medical emergency in which the clinical management/medical treatment of the volunteer would be altered by knowledge of the group assignment.

The unblinded information will be restricted to only a small group of individuals involved in clinical management/medical treatment of the volunteer (e.g., treating physician) and the blind will be maintained for those individuals responsible for the study assessments

The reasons for unblinding should be documented and the Data Coordinating Centre should be notified. The procedures and contact numbers for unblinding are outlined in the SOM.

7.10 Referral to Long Term Follow-Up Study

To assess the long-term safety of the Investigational Product, all study volunteers will be offered participation in a long-term follow-up study with health

assessments, including HIV testing. It is possible that blood will also be collected to assess the persistence of the immune response to the vaccine. Separate informed consent will be obtained for the Long-Term Follow-Up study.

7.11 Social Impact Assessment

A social impact questionnaire will be administered at time points indicated in the Schedule of Procedures (Appendices A, B and C) to assess the impact of trial participation on the volunteer's daily life..

8.0 INVESTIGATIONAL PRODUCT

8.1 Description

8.1.1 F4co/AS01

F4co/AS01 HIV candidate vaccine is composed of 10 µg of F4co, a lyophilized recombinant protein, and a GSK Biologicals' proprietary liquid adjuvant system belonging to the AS01 family. The AS01 adjuvant system is used to reconstitute the lyophilized antigen immediately prior to injection.

F4co is a fusion protein that comprises 4 HIV-1-derived antigens (p24-RT-Nef-p17):

- p24, a viral capsid protein coded by the *gag* gene
- RT (reverse transcriptase), a viral enzyme responsible for transcribing the viral RNA into double-stranded DNA. This enzyme was mutated in one amino acid (Tryptophan 229 substituted by Lysine) to remove the RT polymerase activity. This protein is coded by the *pol* gene
- Nef, a regulatory protein coded by an open-reading-frame (ORF) that flanks the *env* gene. Nef as part of the F4 construct shows absence of biological activity.
- p17, a viral matrix protein coded by the *gag* gene.

The AS01 adjuvant system family is composed of immunostimulants, STIMULON® QS-21 (a triterpene glycoside purified from the bark of *Quillaja saponaria*) and MPL® (3-D Monophosphoryl lipid A), with liposomes as vehicles for these immunostimulants.

There are two formulations in the AS01 family, $AS01_B$ adjuvant system and $AS01_E$ adjuvant system. $AS01_E$ contains half the quantity of immunostimulants of $AS01_B$. The HIV candidate vaccine is named either F4co/$AS01_B$ or F4co/$AS01_E$, depending on the AS01 formulation being used.

F4co protein is supplied as a freeze-dried pellet in a 3 mL clear glass vial, closed with a grey rubber stopper and sealed with a central tear-off aluminum cap. Each vial contains one human dose of antigen corresponding to 10 µg of F4co. The AS01 adjuvant is an opalescent colorless liquid. Each 3 mL glass vial contains a single dose of adjuvant, e.g., at least 0.5 mL of AS01.

8.1.2 Ad35-GRIN

Ad35-GRIN is a recombinant replication-defective adenovirus serotype 35 and contains HIV-1 subtype A *gag*, *reverse transcriptase, integrase*, and *nef* genes (abbreviated as GRIN). The genes were designed as a fusion product, and codon optimized for human cell expression and translation. Mutations were introduced into the sequence to abrogate functional activity.

The vaccine is supplied as a frozen sterile solution in a 4-mL vial with a butyl stopper and aluminum seal. Each vial contains 0.725 mL of vaccine. The volume of administration is 1.0 mL, which will deliver a final dosage of $2x10^{10}$ vp per dose. In order to administer 1.0mL, two vials will be used for each administration. The entire volume from the first vial will be drawn up and sufficient volume from the second vial to make 1.0 ml will be drawn into a single syringe and administered. The dose of the vaccine is expressed as a total virus particle (vp) count measured by HPLC. The vaccine is prepared in formulation buffer composed of Tris 10 mM pH 8.5, Sucrose 342.3 g/L, 1mM $MgCl_2$, Tween80 54 mg/L and 150mM NaCl in water for injection.

8.1.3 Placebo

The associated placebo is Saline (NaCl 0.9%), produced and released by GSK Biologicals.

The summary of the Investigational Product is shown in Table 2.

Table 2
Formulation of Investigational Product

Vaccine/Placebo	Dosage Level	Total Volume in Vial (mL)	Total Volume to be injected (mL)	Route of Administration
F4co/$AS01_E$	10 μg	At least 0.5 mL	0.5 mL	IM
F4co/$AS01_B$	10 μg	At least 0.5 mL	0.5 mL	IM
Ad35-GRIN	$2x10^{10}$ vp	0.725 mL	1.0 mL (using 2 vials)	IM
Saline	NA	0.7 mL	0.5 mL	IM
Saline for blinding with Ad35-GRIN	NA	0.7mL	1.0 mL (using 2 vials)	IM

IM = Intramuscular

8.2 Shipment and Storage

Authorization to ship the Investigational Product to the CRC will be provided in writing by the sponsor, upon confirmation that all required critical documents for shipment authorization are completed. The Investigational Product will be shipped to the CRCs according to the required storage conditions.

F4co vaccine, $AS01_E$ & $AS01_B$ adjuvants, Placebo vials are stored at +2° to +8°C and Ad35-GRIN is stored at -70°C or below as recommended by the manufacturer. Each of the above drug products is identified by unique lot

numbers. Vials are labelled under Good Manufacturing Practice (GMP) in order to support a double-blinded trial and will be in compliance with regulatory requirements. Separate boxes containing each of the drug products, namelyF4co vaccine, $AS01_E$ adjuvant, $AS01_B$ adjuvant, Placebo vials and Ad35-GRIN vaccine will be packaged in separate cartons and shipped appropriately to the CRCs for use.

8.3 Dispensing and Handling

The Investigational Product will be dispensed as specified in the SOM. Designated study staff will ensure that the assignment matches the allocation number assigned to the volunteer.

8.4 Administration

Investigational Product will be administered at time points according to the Schedule of Procedures (Appendices A, B and C).

The preferred site of first intramuscular administration is the deltoid muscle of the non-dominant arm (for example, injection in the left arm if volunteer uses mainly right arm). In the co-administration group, the two vaccines will be given 2-4 cm apart intramuscularly preferably in the deltoid muscle of the non-dominant arm. It is suggested that the same arm is used for each vaccination.

Further information on the administration of the Investigational Product is supplied in the SOM.

8.5 Accountability and Disposal

In case of any temperature deviation for F4co, $AS01_B$, $AS01_E$ or NaCl, official written approval for the use of vaccine must be obtained from GSK via the Sponsor. In case of any temperature deviation for Ad-35-GRIN, official written approval for the use of vaccine must be obtained from the Sponsor.

All used vials will be returned to the Investigational Product dispenser or pharmacy at the end of each vaccination visit. The date, vial allocation number and location of storage of the returned vials will be recorded.

During the study, the Investigational Product accountability form, the dispensing log and the log of returned vials will be kept and monitored.

At the end of the study, the used and unused vials will be handled according to instructions of Sponsor. Further information on Accountability and Disposal is supplied in the SOM.

9.0 ASSESSMENTS

9.1 Safety Assessments

Data on local and systemic solicited adverse events will be collected by structured interview, using specific questions. Data on other adverse events will

be collected with an open-ended question. All data will be recorded on the appropriate source documents.

The local and systemic solicited adverse events will be collected and the vital signs will be measured by study staff prior to vaccination and at least 30 minutes post-vaccination as specified in the Schedule of Procedures (Appendices A, B and C)

9.1.1 Local solicited adverse events

The presence of local solicited adverse events will be assessed at the time points specified in the Schedule of Procedures (Appendices A, B and C).

Local solicited adverse events (pain, tenderness, erythema/skin discoloration, induration, oedema) will be assessed and graded using the Appendix D, Adverse Event Severity Assessment Table as a guideline.

9.1.2 Systemic solicited adverse events

The presence of systemic solicited adverse events will be assessed at the time points specified in the Schedule of Procedures (Appendices A, B and C).

Fever, chills, headache, malaise, sweating, fatigue, myalgia, arthralgia, nausea, vomiting, diarrhoea and abdominal pain, will be assessed and graded using the Appendix D, Adverse Event Severity Assessment Table as a guideline.

9.1.3 Vital Signs

At the vaccination visits, vital signs (pulse, respiratory rate, blood pressure and temperature) will be measured by study staff prior to vaccination and at least 30 minutes post-vaccination.

For the other study visits vital signs will be assessed at the time points specified in the Schedule of Procedures (Appendices A, B and C).

9.1.4 Other adverse events

Occurrence of other adverse events, including autoimmune disease and connective tissue disorders (including Serious Adverse Events) will be collected following an open question to volunteers on the time points according to the Schedule of Procedures (Appendices A, B and C). The adverse events will be graded using the Appendix D, Adverse Event Severity Assessment Table as a guideline.

For more information regarding adverse events refer to Section 10.0, Adverse Events.

9.1.5 Concomitant Medications

During the study, information regarding concomitant medications and reasons for their use will be solicited from the study volunteers at each visit and recorded.

Concomitant receipt of Investigational Products, including other HIV vaccines is prohibited during the study.

If clinically indicated, non-live vaccines (non-HIV) and immunoglobulin may be given up to 14 days before study vaccination(s) or after post-vaccination blood draw (i.e., 14 days or more after any study vaccinations).

Live-attenuated vaccines (non-HIV) may be given 60 days before study vaccination(s) or 60 days after the post-vaccination blood draw (live attenuated flu vaccine may be given within 14 days before study vaccination(s) or after the 14-day post-vaccination blood draw). However, the study vaccination(s) should not be given if there are any continuing symptoms from recently administered non-HIV vaccines. In this situation, the CRC investigator should consult with IAVI Medical Monitor before administering the next study vaccination.

If the use of a short tapering (<2 weeks) course of oral corticosteroids is required, the study vaccinations may be continued after a 4 week washout period provided that the medical condition requiring this therapy has resolved completely and in the opinion of both the CRC investigator and IAVI Medical Monitor the continuation of the study vaccinations will not jeopardize the safety of the volunteer. Volunteers requiring chronic (> 2 weeks) or long term therapy will not receive any further vaccinations but will continue with follow-up visits until the end of the study.

9.1.6 Routine laboratory parameters

Table 3 shows the laboratory parameters that will be measured routinely. These will include haematology, clinical chemistry, immunological assays and urinalysis. The samples for these tests will be collected at the time points indicated in the Schedule of Procedures (Appendices A, B and C).

Table 3
Laboratory Parameters

Laboratory Parameter	Test
Haematology	Full blood count (haemoglobin, haematocrit, erythrocytes, leucocytes, platelets).<br>Differential count, absolute neutrophil count, absolute lymphocyte count
Clinical Chemistry	Liver function tests: aspartate transferase (AST), alanine transferase (ALT), total and direct bilirubin.<br>Kidney function: creatinine

Immunology	CD4+ and CD8+ T cells (absolute count)
Urinalysis	Dipstick test for protein, blood glucose, ketones, esterase (leukocytes), nitrite. If abnormalities (blood, protein, leucocytes) are found on dipstick test then further test will be performed (e.g., microscopy, culture)

9.1.7 Specific screening tests:

Volunteers will be screened to exclude the following diseases:

- Hepatitis B: positive for hepatitis B surface antigen (HBsAg)
- Hepatitis C: positive for hepatitis C antibodies (HCV antibodies)
- Syphilis: confirmed diagnosis of active syphilis (RPR and TPHA or equivalent)
- Tuberculosis: In volunteers presenting with productive cough of more than 15 days duration, a chest x-ray and sputum examination for Acid Fast Bacilli will be performed to rule out active tuberculosis

Volunteers will also be screened and excluded for the following:

- Seropositivity for Ad35-specific neutralizing antibody

9.2 Immunogenicity Assessments

9.2.1 Antibody Responses

Antibodies against vaccine encoded HIV proteins will be measured according to time points indicated on the Schedule of Procedures (Appendices A, B and C). Further antibody testing such as avidity or other functional antibody assays will be conducted if warranted.

Samples for neutralizing antibodies against the Ad35 vector will be taken at time points as indicated in the Schedule of Procedures (Appendices A, B and C).

9.2.2 Cellular Responses

Assays will be performed according to the IAVI Human Immunology laboratory and GSK SOPs as specified in the LAP Standard reagents will be used.

Immunogenicity assays, including ELISPOT and intracellular cytokine staining (ICS) assays for monitoring the number of circulating T cells that can be stimulated to produce cytokines, will be performed at time points indicated in the Schedule of Procedure (Appendices A, B and C), using peptide pools and/or proteins representing all or a portion of the vaccine encoded (GRIN and F4Co) antigen(s). Where feasible, ELISPOT assays will be performed on site using fresh or cryopreserved PBMC.

Pending the results of the secondary immunogenicity assays (ELISPOT and ICS), further exploratory studies may be carried out to look at the

breadth of the response via epitope mapping using vaccine matched peptides and cross-reactive epitopes using peptides from different HIV-subtypes. Vaccine induced T cell responses may be further characterized for HLA restriction and additional markers on the responding cells, such as markers for memory, activation, function or markers for homing to mucosal tissues. The ability of PBMC to restrict the growth of HIV in vitro may be examined using a Virus Inhibition Assay. The analytical plan for the immunology studies will be developed between IAVI, GSK and the CRC teams and an algorithm will be applied to determine which samples and time points are prioritized for exploratory assays.

9.2.3 PBMC, Serum and Plasma Storage

Samples of cryopreserved PBMC, plasma and serum will be taken at time points as indicated in the Schedule of Procedures (Appendices A, B and C) for purposes of standardisation, quality control and for future assays related to HIV vaccine research and development. These samples will be archived and only the volunteer identification number will identify the samples.

For the immunogenicity assessments, the laboratory personnel will be trained as necessary by the sponsor and provided with a written procedure manual. The sponsor will also provide specific instructions on reagents.

The samples described in 9.2.2 and 9.2.3 will be shipped routinely from each CRC to the IAVI Human Immunology Laboratory and the GSK laboratory as indicated in the Schedule of Procedures (Appendices A, B and C). The immunological testing will be performed at the CRCs, IAVI Human Immunology Laboratory and the GSK laboratory in accordance with organization standard SOPs and standard reagents.

9.3 Other Assessments

9.3.1 HLA (Human Leukocyte Antigen) Typing

Samples for HLA typing will be collected at the single time point indicated in the Schedule of Procedures (Appendices A, B and C).

HLA typing will be performed on samples that are selected for epitope mapping, VIA assays or other assay where the HLA type will inform the testing or interpretation of results.

9.3.2 HIV test

Samples will be tested at the time points indicated in the Schedule of Procedures (Appendices A, B and C). Further information is specified in Section 11.1 HIV Testing.

9.3.3 Pregnancy Test

A urine pregnancy test for all female volunteers will be performed by measurement of Human Chorionic Gonadotrophin (βhCG) at the time points indicated in the Schedule of Procedures (Appendices A, B and C).

The results of the pregnancy test must be negative prior to vaccination.

9.3.4 Ophthalmic Examination

The eye examination will be performed prior to the first vaccination and within one month of the final study visit in 100% of the volunteers enrolled in 2 centres (approximately 50% of enrolled volunteers). The purpose of this is to assess the effects of the investigational vaccine on structures and tissues of the eye, including lens opacities. The eye examination will be performed according to procedures detailed in the SOM.

10.0 ADVERSE EVENTS

10.1 Definition

An adverse event (AE) is any untoward medical occurrence in a volunteer administered Investigational Product and which does not necessarily have a causal relationship with the Investigational Product. An AE can therefore be any unfavourable or unintended sign (including an abnormal laboratory finding), symptom, or disease, temporally associated with the use of the Investigational Product whether or not related to the Investigational Product.

10.2 Assessment of Severity of Adverse Events

Assessment of severity of all AEs is ultimately the responsibility of the principal investigator.

The following general criteria should be used in assessing adverse events as Grade 1, 2, 3, or 4 if not identified elsewhere in the Adverse Event Severity Assessment Table (Appendix D).

- Grade 1: Symptoms causing no or minimal interference with usual social & functional activities;
- Grade 2: Symptoms causing greater than minimal interference with usual social & functional activities;
- Grade 3: Symptoms causing inability to perform usual social & functional activities;
- Grade 4: Symptoms causing inability to perform basic self-care functions OR Medical or operative intervention indicated to prevent permanent impairment, persistent disability, or death

Guidelines for assessing the severity of specific adverse events and laboratory abnormalities are listed in Appendix D, Adverse Event Severity Assessment Table.

10.3 Relationship to Investigational Product

The relationship of an (S)AE is assessed and determined by the Principal Investigator or designee. All medically indicated and available diagnostic methods (e.g., lab, blood smear, culture, X-ray, etc.) should be used to assess the nature and cause of the AE/SAE. Best clinical and scientific judgment should be used to assess relationship of AE/SAEs to the Investigational Product and/or other cause.

The following should be considered for the assessment of relationship of adverse events to the investigational Product:

- Presence/absence of a clear temporal (time) sequence between administration of the Investigational Product and the onset of AE/SAE
- Presence/absence of another cause that could more likely explain the AE/SAE (concurrent disease, concomitant medication, environmental or toxic factors, etc.)
- Whether or not the AE/SAE follows a known response pattern associated with the investigational Product

The relationship assessment should be reported as one of the following:

Not Related: clearly explained by another cause (concurrent disease, concomitant medication, environmental or toxic factors, etc.).

Unlikely: more likely explained by another cause (concurrent disease, concomitant medication, environmental or toxic factors, etc.).

Possibly: equally likely explained by another cause but the possibility of the Investigational Product relationship cannot be ruled out (e.g., reasonably well temporally related and/or follows a known Investigational Product response pattern but equally well explained by another cause).

Probably: more likely explained by the Investigational Product (e.g., reasonably well temporally related and/or follows a known Investigational Product response pattern and less likely explained by another cause).

Definitely: clearly related and most likely explained by the Investigational Product

For the purpose of expedited safety reporting, all possibly, probably or definitely related SAEs are considered Investigational Product related SAEs.

10.4 Serious Adverse Events

An adverse event is reported as a "Serious Adverse Event" if it meets any the following criteria (as per ICH GCP Guidelines):
- Results in death
- Is life threatening

- Results in persistent or significant disability/incapacity.
- Requires in-patient hospitalization or prolongs existing hospitalization.
- Is a congenital anomaly/birth defect or spontaneous abortion.
- Any other important medical condition that requires medical or surgical intervention to prevent permanent impairment of a body function or structure.

Serious Adverse Events (SAEs) should be reported to IAVI within 24 hours of the CRC becoming aware of the event. All SAEs should be reported using the designated SAE Report Form and sent to the sponsor according to SAE Reporting Guidelines (see SOM).

To discuss Investigational Product related SAEs or any urgent medical questions related to the SAE, the CRC investigator should contact one of the IAVI medical monitors directly (see the Contact List).

The IAVI SAE Report Form should be completed with all the available information at the time of reporting. The minimum data required in reporting an SAE are the volunteer identification number, date of birth, gender, event description (in as much detail as is known at the time), onset date of event (if available), reason event is classified as Serious, reporting source (name of principal investigator or designee), relationship assessment to the Investigational Product by the investigator.

The Principal Investigator or designee is required to write a detailed written report with follow up until resolution or until it is judged by the principal investigator or designee to have stabilized.

The Principal Investigator or designee must notify the local IRB/IEC of all SAEs as appropriate. In case of Investigational Product related SAEs, the sponsor will ensure the local regulatory authorities are notified as well as the Safety Review Board (SRB), and other research centres where the same Investigational Product is being tested.

More details on SAE definitions and reporting requirements are provided in the SAE Reporting Guidelines (see SOM).

10.5 Clinical Management of Adverse Events

Adverse events (AEs) will be managed by the clinical study team who will assess and treat the volunteer as appropriate, including referral. If any treatment/medical care is required as a result of the harm caused by the Investigational Product or study procedures, this care will be provided free of charge.

If a volunteer has an adverse event and/or abnormal laboratory value that is known at the time of study vaccination(s), the specifications of Section 12.0 will be followed.

Volunteers will be followed until the adverse event resolves or stabilizes or up to the end of the study, whichever comes last. If at the end of the study, an adverse event (including clinically significant lab abnormality) that is considered possibly,

probably or definitely related to the Investigational Product is unresolved, follow-up will continue until resolution if possible and the volunteer will be referred.

10.6 Pregnancy

Although not considered an adverse event, if a female volunteer becomes pregnant during the study, it is the responsibility of the Principal Investigator or designee to report the pregnancy promptly to IAVI using the designated forms. However, serious complications of pregnancy that meet SAE criteria specified in the Section 10.4 of this Protocol (e.g., eclampsia, spontaneous abortion, etc.) should be reported as SAEs. If a female volunteer becomes pregnant during the study, vaccinations will be discontinued and the volunteer followed for safety until the end of pregnancy or study completion, whichever occurs last. Approximately 2-4 weeks after delivery, the baby will be examined by a physician to assess its health status and the results will be reported to IAVI.

10.7 Intercurrent HIV Infection

HIV infection cannot be caused by the Investigational Product. If a volunteer is found to be HIV-infected through exposure in the community, study vaccinations must be discontinued and the volunteer followed according to procedures described in Section 12.2.

Intercurrent HIV infection in study volunteers, although not considered an SAE must be reported promptly to IAVI using the designated forms. IAVI will report intercurrent HIV infections using the same procedure as SAE reporting. Serious medical conditions associated with the HIV infection that meet SAE criteria specified in the Section 10.4 of this Protocol (e.g. sepsis, pneumonia, etc.) should be reported as SAEs using the SAE Report Form.

10.8 Serious Event Prior to Investigational Product Administration

If a serious event occurs in the period between the volunteer signing the Informed Consent Form and receiving their first study injection, the event will be reported using the SAE form and following the same procedures for SAE reporting as indicated in Section 10.4. The type of event will be indicated by using the relevant checkbox on the SAE form.

11.0 MANAGEMENT OF HIV ISSUES DURING AND FOLLOWING STUDY

11.1 HIV Testing

Volunteers will be tested for HIV-1 and HIV-2 antibodies as indicated in the Schedule of Procedures (Appendices A, B and C) or as needed, if medical or social circumstances arise.

If the routine post-vaccination HIV Antibody test is positive, a predetermined algorithm will be followed to distinguish between an immune response to the vaccine from an HIV infection through exposure in the community.

To prevent unblinding of the volunteer and study staff, results will be reported to the CRC as “HIV-infected” or “Not infected with HIV-1 or HIV-2”.

If a volunteer is found to be HIV-infected, a newly drawn blood specimen will be collected for confirmation.

Volunteers who have a positive HIV-antibody test(s) as a result of vaccine-induced HIV antibodies rather than a true HIV infection (false positive HIV test) will have their test result reported as “Not infected with HIV-1 or HIV-2” (to prevent unblinding of volunteer and staff) and will be followed up until the test becomes negative. At the end of the study these volunteers will be informed of the false-positive test result and offered continued follow-up.

Should a volunteer require an HIV test outside the study for personal reasons, it is recommended that the volunteer contact the study staff first. The HIV test can be drawn at the CRC and then processed at an independent laboratory as above. Written evidence of HIV status (HIV-infected or HIV-uninfected) will be provided upon request.

All volunteers will receive HIV risk reduction counselling and pre-HIV-test and post-HIV-test counselling as specified in Section 11.3.2.

11.2 Social Discrimination as a Result of an Antibody Response to Vaccine

The aim is to minimize the possibility of social discrimination in volunteers (if any) who develop vaccine-induced HIV antibodies and test positive on a diagnostic HIV antibody test. Appropriate diagnostic HIV testing and certification will be provided both during and after the study as needed. In addition, the CRCs will have the option of offering the volunteer a card stating that he/she participates in a research study.

11.3 HIV infection

Volunteers who are found to be HIV infected at screening and volunteers who acquire HIV infection during the study will be provided the following:

11.3.1 Counselling

The volunteer will be counselled by the study counsellors. The counselling process will assist the volunteer with the following issues:

- Psychological and social implications of HIV infection
- Who to inform and what to say
- Implications for sexual partners
- Implications for child-bearing
- Avoidance of transmission to others in future

11.3.2 Referral for Support and/or Care

Volunteers will be referred to a patient support centre or institution of his/her choice for a full discussion of the clinical aspects of HIV infection. Referral will be made to a designated physician or centre for discussion of options of treatment of HIV-infection.

For those individuals who become HIV infected after enrolment in the study, antiretroviral therapy will be provided when clinically indicated according to accepted treatment guidelines. According to IAVI's Treatment and Care Guidelines, antiretroviral therapy will be provided at no charge for up to 5 years after treatment is initiated, if not available free of charge elsewhere.

Volunteers who acquire HIV infection during the study, despite counselling, will complete the Early Termination visit as detailed in the Schedule of Procedures (Appendices A, B and C) and then be referred to IAVI Protocol H, a separate research protocol for follow-up of HIV infected volunteers with clinical, laboratory and immunology assessments.

HIV-infected pregnant women will be referred for prenatal care and to a program for the Prevention of Mother to Child Transmission (PMTCT). A volunteer who becomes pregnant will be followed according to the timeline as specified in Section 10.6

12.0 DISCONTINUATION OF VACCINATIONS AND/OR WITHDRAWAL FROM STUDY

12.1 Discontinuation of Vaccinations

Any volunteer discontinuing from further vaccinations or being considered for discontinuation of vaccinations will be discussed with the sponsor. Volunteers will be discontinued from further vaccination for any of the following reasons:

1. Pregnancy
2. Intercurrent HIV Infection
3. Use of systemic corticosteroids, immunosuppressive, antiviral, anticancer, antituberculosis or other medications considered significant by the investigator
4. A disease or condition or an adverse event that may develop, regardless of relationship to the Investigational Product, if the principal investigator or designee is of the opinion that further study vaccinations will jeopardize the safety of the volunteer
5. Any of the following current abnormal laboratory parameters which are known at the time of vaccination:

   Haematology
   - Haemoglobin <9.0 g/dL **or** <1.40 mmol/L
   - Absolute Neutrophil Count (ANC): < 1000/mm3 **or** < 1.0 x 10^9/L
   - Absolute Lymphocyte Count (ALC): ≤ 500/mm3 **or** ≤ 0.5 x 10^9/L
   - Platelets: ≤ 90,000 ≥ 550,000/mm3 **or** ≤ 90 x 10^9 ≥ 550 x 10^9/L

   Chemistry
   - Creatinine: >1.4 x ULN
   - AST: >3.0 x ULN
   - ALT: >3.0 x ULN

Urinalysis: Dipstick 3+ confirmed by microscopy for
- Protein
- Blood (not due to menses)
- Leukocytes

6. Receipt of live attenuated vaccine within the previous 60 days (live attenuated flu vaccine within 14 days) or planned receipt within 60 days after vaccination with Investigational Product or receipt of other vaccine within the previous 14 days or planned receipt within 14 days after vaccination with Investigational Product
7. A Grade 4 local reactogenicity event (as per Appendix D) involving the injected arm
8. Anaphylaxis; bronchospasm; laryngeal oedema; convulsions or encephalopathy following study vaccinations.
9. Life threatening adverse event following study vaccinations unless not related to the Investigational Product and fully resolved.
10. Any immediate hypersensitivity reaction judged to be related to the Investigational Product, if the principal investigator or designee is of the opinion that further study vaccinations will jeopardize the safety of the volunteer
11. Volunteer requests to discontinue further vaccination.
12. Participating in another clinical study of an Investigational Product

12.2 Follow Up After Discontinuation of Further Vaccinations

Volunteers, in whom study vaccinations are discontinued due to adverse events, will be followed until the adverse event resolves or stabilizes or up to the end of the study, whichever comes last. These volunteers will not be replaced.

Follow-up of pregnant volunteers will be done as specified in Section 10.6.

12.3 Withdrawal from the Study (Early Termination)

Volunteers may be withdrawn from the study permanently for the following reasons:
1. Volunteers may withdraw from the study at any time if they wish, for any reason.
2. The principal investigator or designee has reason to believe that the volunteer is not complying with the protocol.
3. If the sponsor decides to terminate or suspend the study.

If the volunteer withdraws from the study, all early termination visit procedures will be performed according to the Schedule of Procedures (Appendices A, B and C) where possible. Every effort will be made to determine and document the reason for withdrawal from the study.

13.0 DATA HANDLING

13.1 Data Collection and Record Keeping at the Clinical Research Centre

Data Collection: All study data will be collected by the clinical study staff using designated source documents and entered onto the appropriate case report forms (CRFs). CRFs will be provided by IAVI and should be handled in accordance with the instructions from IAVI. All study data must be verifiable to the source documentation. A file will be held for each volunteer at the clinic(s) containing all the source documents. Source documentation will be available for review to ensure that the collected data are consistent with the CRFs.

All CRFs and laboratory reports will be reviewed by the clinical team, who will ensure that they are accurate and complete.

Source documents and other supporting documents will be kept in a secure location.

Standard GCP practices will be followed to ensure accurate, reliable and consistent data collection.

Source documents include but are not limited to:

- Signed Informed Consent Documents
- Dates of visits including dates of vaccinations
- Documentation of any existing conditions or past conditions relevant to eligibility
- Reported laboratory results
- All adverse events
- Concomitant medications
- Local and systemic reactogenicity events

13.2 Data Collection and Transfer at the Human Immunology Laboratory

Immunogenicity data generated at the CRCs, IAVI Human Immunology and GSK Laboratories will be entered or transferred into the Laboratory Information Management System (LIMS) and then transferred directly to the DCC by the designated party according to the Laboratory Analytical Plan (LAP). Immunogenicity data generated by GSK Laboratories will be transferred directly to the DCC in a suitable format

13.3 Data Entry at the Clinical Research Centre

The data collected at the CRC will be entered onto the CRFs by the study staff. To provide for real time assessment of safety, data should be entered as soon as reasonably feasible (e.g., within one week) after a visit. Immunogenicity results will be transferred within 2 weeks after the assay is performed.

13.4 Data Analysis

The data analysis plan will be developed and agreed upon by the sponsor, GSK and the Principal Investigators. The statistician at the Data Coordinating Centre in collaboration with the Principal Investigator and the Sponsor will create tables according to this data analysis plan.

The DCC will conduct the data analysis and will provide interim and final study reports to the sponsor who will share the report with GSK, the principal investigators, the SRB and the regulatory authorities as appropriate.

14.0 STATISTICAL CONSIDERATIONS

14.1 Sample Size

A total of 140 volunteers (28 vaccine/7 placebo per group) will be entered into each of the 4 groups scheduled to receive F4co (formulated in $AS01_B$ or $AS01_{E)}$ vaccine or placebo and Ad35-GRIN vaccine or placebo.

14.2 Statistical Power and Analysis

The small sample size is appropriate for an exploratory study of a novel product while safety, tolerability and immunogenicity of the vaccine combinations are investigated.

For safety and immunogenicity analyses, the placebo recipients from Groups A, and B will be combined.. However, for analyses where the schedule is not expected to have an impact, all placebo groups will be pooled together.

14.2.1 Safety and Tolerability

The rate of local and systemic reactogenicity will be used to assess the safety of the Investigational Products and proposed schedules.

The rate of Serious Adverse Events related to the Investigational Product will be used as one measure of the safety of the Investigational Products. Adverse Events that may be temporarily incapacitating (for example, loss or cancellation of work or social activities), which could make an Investigational Product impractical for large scale use if they occur in more than a small proportion of cases, will also be assessed.

All adverse events will be reported, grouped as to whether or not they qualify as SAEs, their severity assessment, and their relationship to the Investigational Product (as judged by the investigator).

The proportion of volunteers in each group who develop HIV infection will be analyzed.

Power Considerations

For Grade 4 (very severe) adverse events related to Investigational Product: if none of the volunteers receiving the combination of Investigational Products experiences such reactions, the 2-sided 95% upper confidence bound for the rate of these adverse events in the population is 0.03 (n=112) and 0.12 (n=28), by the Clopper-Pearson method.

There is limited power for comparison of event rates between treatment arms. For example, with 80% power, the minimum detectable differences in event rates between the placebo (p) arm and the vaccine (v) arms are 50% (when n_p=7, n_v=28) and 28% (when n_p=14, n_v=56), assuming a one-percent incidence rate in the placebo arm (one-sided Fisher's exact test with significance level of 0.05).

Planned Statistical Analyses:

Considering the low power available for group comparison and the associated risk of false safety signal in the context of multiple exploratory analyses, the analysis of safety events will be based on within-group descriptive summaries for the incidence rate of each event. The associated 95% confidence intervals for the incidence rate within each group will also be provided.

Interim analyses of grouped data will be carried out without unblinding the study to investigators or volunteers. At 1 month after the end of the vaccination regimen and at the end of the study, an analysis will be performed according to a pre-specified statistical analysis plan.

14.2.2 Vaccine Immunogenicity

Cellular immune responses will be analyzed using binomial methods to examine for the presence or absence of HIV specific T-cell responses quantified by ELISPOTand ICS. In addition, ICS magnitude will be analyzed based on log-normal distribution assumptions.

The Geometric Mean Titer (GMT) of anti-HIV antibodies will be assessed and the presence or absence of antibodies analyzed using binomial methods.

Response Criteria for Immunogenicity Endpoints:

IFN-γ ELISPOT: Responses are expressed as spot forming cells/million (SFC/10^6) PBMC. The GRIN peptides matching the Ad35 vaccine have been qualified using 55 seronegative PBMC specimens. Positive responses are defined as: 1) A Mock-subtracted response greater than 38 SFU/10^6 PBMC across all peptides 2) Mean replicate count >4x mean Mock count 3) Coefficient of Variation among replicate counts ≤70% 4) At least 3 replicates 5) Acceptance criteria for each sample comprises of the following; the average background < 50 and a response to PHA must be present. Peptides matched to the F4co vaccine will be qualified to determine a similar cut-off for the IFN-γ ELISPOT.

IL-2 ELISPOT: A cut-off for the IL-2 ELISPOT will be assessed after qualifying the assay with GRIN and F4co peptides and/or F4co proteins. Cut-offs based on pre-vaccination responses may also be used.

ICS: ICS data are expressed as antigen-specific CD4+ CD40L+T-cells per million CD4+ T-cells or per hundred CD4+ T-cells (percentage). The

frequency of antigen specific CD4+ CD40L+ T cells are calculated as the difference between the frequency of CD4+ CD40L+ T cells expressing immune markers (among IFN-γ, IL-2 and TNF-α), upon in vitro stimulation with the peptide pools minus the frequency of CD4+ CD40L+ T cells expressing these immune markers upon in vitro stimulation with medium only. Differences less or equal to zero are imputed to 1 antigen specific immune marker expressing CD4+ CD40L+ T cell per 10^6 CD4+ T cells. The Geometric Mean calculations are performed by taking the anti-log of the mean of the log transformations. Frequency of CD4+ CD40L+ T-cells expressing markers to the fusion protein F4co (all antigens) is estimated by adding individual frequencies of CD4+ CD40L+ T-cells to each of the 4 antigens (p17, p24, Nef, RT). A responder is a subject with antigen-specific CD4+ response greater than or equal to the cut-off value. The cut-off will be chosen on the basis of pre-vaccination response for all subjects, by examining percentiles (95th percentile) in response to the different antigens.

Likewise ICS data are expressed as antigen-specific CD8+ T-cells per million CD8+ T-cells or per hundred CD8+ T-cells (percentage). Calculation of the frequencies of antigen specific CD8+ T cells and definition of a responder are both based on the same rules as for the antigen specific CD4+ T cells responses.

Anti-HIV antibody ELISA: IgG antibody responses to the fusion protein F4co and to the p24, RT, p17 and Nef antigens are assessed using standard ELISA techniques. A seropositive subject is a subject whose titre is greater than or equal to the cut-off value. The cut-off values for p17, p24, RT, Nef and F4co antibodies is 187 mEU/ml, 119 mEU/ml, 125 mEU/ml, 232 mEU/ml, and 42 mEU/ml, respectively. The seropositivity rate is defined as the percentage of seropositive subjects. Geometric Mean Concentrations (GMCs) are calculated by taking the anti-log of the mean of the log value transformations. Antibody values below the cut-off of the assay are given an arbitrary value of half the cut-off for the purpose of GMC calculation.

Ad35 neutralization titre: Ad35 Neutralization titre is measured in volunteers' serum using a qualified cell based assay. In this assay target A549 cells (human lung carcinoma) are infected with a pre-determined amount of Luciferase-encoding Ad35 virus in the presence of serially diluted volunteer's serum. Luciferase activity is measured after 48h of incubation and neutralization titre is inferred by plotting luciferase activity over serum dilution (or titre). From this plot we measure the inhibitory concentration at which 90% of the Ad35 infection is inhibited (EC90). If EC90 is inferior to 1/16 serum dilution the volunteer is considered Ad35 neutralization negative. Volunteers for which EC90 is equal or greater than 1/16 are considered positive.

Power Considerations

Contrast between vaccine and placebo groups: The study has at least 80% power (one-sided Fisher's exact test, with α=0.05) to detect a

difference of 50%, 53% and 56% or more in the rate of positive responses between an active group (n_1=28) and the placebo group (n_2=7), assuming the rate of positive responses in the placebo group to be 1%, 5% and 10%, respectively.

Contrast between two active vaccine groups: The minimum detectable difference between the two vaccine groups is 28%, with α=0.05 and 80% power (two-sided Fisher's exact test), based on an assumption of 1% positive response rate in one of the two groups. If the response rate in one group was 10% and 20%, the minimum detectable difference would be 35% and 39%, respectively (i.e., the corresponding response rates in the other group would have to be 45% and 59%).

The criterion to be used for the demonstration of non-inferiority of the immune response induced by F4co/$AS01_E$ compared to F4co/$AS01_B$ is the following:

> The upper limit of the 95 % CI for the ratio of the magnitude of the CD4+ CD40L+ T cells expressing at least IL-2, between both groups, at 2 weeks post second vaccination, is below 1.5.

Power considerations for the comparison of $AS01_E$ to $AS01_B$ are based on the values observed for the number of CD4+ T cells expressing at least IL-2 in GSK study PRO-HIV-005. A sample size of 27 subjects per group will provide a power of 80% to either exclude a difference higher than 1.5 fold between both adjuvants, assuming the same intensity for the CD4 T cell expressing at least IL-2 (with a one-sided alpha level of 0.025 and assuming a standard deviation of 0.223 for the $\log_{10}$ intensity).

Planned Statistical Analyses

At 1 month after the end of the vaccination regimen and at the end of the study, an analysis will be prepared according to a pre-specified statistical analysis plan.

Based on the previous experience with IAVI Phase I/II Investigational Product studies, it is expected that the amount of missing, unused or spurious data will be insignificant. Unused and spurious data will be listed separately and excluded from the statistical analysis. Missing data will be excluded from the statistical analysis.

15.0 QUALITY CONTROL AND QUALITY ASSURANCE

To ensure the quality and reliability of the data gathered and the ethical conduct of this study, a SOM has been developed.

Regular monitoring will be performed according to ICH-GCP as indicated in Section 17.3.

An independent audit of the study may be performed, if appropriate at the discretion of the Sponsor.

By signing the protocol, the Principal Investigators, agree to facilitate study related monitoring, audits, IRB/IEC review and regulatory inspection(s) and direct access to source documents. Such information will be treated as strictly confidential and under no circumstances will be made publicly available.

16.0 DATA AND BIOLOGICAL MATERIAL

All data and biological material collected through the study shall be managed in accordance with the Clinical Trial Agreement. Distribution and use of those data will be conducted by agreement of both parties, with the understanding that the data will be published (Section19 Publication).

The computerized raw data generated will be held by the DCC on behalf of the sponsor. The study site will also hold the final data files and tables generated for the purpose of analysis. Principal investigators or designees will have access to the clinical study database with appropriate blinding.

17.0 ADMINISTRATIVE STRUCTURE

The Principal Investigator will be responsible for all aspects of the study at the CRC.

17.1 Protocol Safety Review Team (PSRT)

A PSRT will be formed to monitor the clinical safety data. During the vaccination phase of the trial, the PSRT will review the clinical safety data on a weekly basis via electronic distribution of reports. An ad hoc PSRT review meeting will occur if any of the members of the PSRT requests a special review to discuss a specific safety issue or as specified in the Study Operations Manual.

The PSRT will consist of the IAVI Medical Monitor (PSRT chair), IAVI Medical Safety Monitor and Principal Investigators or a medically qualified delegate of the Principal Investigator. The IAVI CMO and GSK medical experts are ex officio members.

17.2 Safety Review Board (SRB)

The Safety Review Board (SRB) will oversee the safety monitoring of the study. The SRB will consist of independent clinicians/scientists/statisticians who are not involved in the study. Investigators responsible for the clinical care of volunteers or representative of the sponsor may not be a member of the SRB.

However, the SRB may invite the Principal Investigator or designee, sponsor and GSK representatives to an open session of the meeting to provide information on study conduct, available data or to respond to questions.

The SRB will review the blinded study data for the first 28 volunteers in groups A/B, for the first 14 volunteers in groups C and for the first 14 volunteers in group

D at 2 weeks post month 1, and 2 weeks post month 4 vaccinations. Interim reviews may be specifically requested (see Section 17.1.2).

17.2.1 Content of Interim Review

The SRB will be asked to review the following data:

- All clinical adverse events/reactogenicity judged by the Principal Investigator or designee to be possibly, probably or definitely related to the Investigational Product

- All laboratory adverse events confirmed on retest and judged by the Principal Investigator or designee to be possibly, probably, or definitely related to Investigational Product

- All Serious Adverse Events, independent of relationship to the Investigational Product.

17.3 Criteria for Pausing the Study

Enrollments and vaccinations will be stopped, and a safety review conducted by the SRB for any of the following criteria:

- one subject experiences a serious adverse event which is probably or definitely related to the investigational vaccine(s), or two or more subjects experience similar serious adverse events which are possibly related to the investigational vaccine(s);
- there is a subject death assessed as possibly, probably or definitely related to the investigational vaccine(s);
- one subject with injection site ulceration, sterile abscess or necrosis associated with vaccine administration occurs; or
- one subject with severe allergic reaction such as laryngospasm, bronchospasm or anaphylaxis associated with vaccine administration occurs.

The sponsor will request a review by the SRB, or the SRB chair if other SRB members cannot be convened, to be held within 2 business days of the sponsor learning of the event(s). Ad-hoc review may be specifically requested by the sponsor, GSK, the Principal Investigators or by the SRB. The study may be unblinded at the discretion of the SRB.

Following this review, the SRB will make a recommendation to the Sponsor, regarding the suspension or continuation of the vaccinations/ study. The Sponsor will inform GSK and the Principal Investigators without delay.

17.4 Study Supervision

The SRB and the IAVI CMO will be provided progress report(s) of this study. Close cooperation will be necessary to track study progress, respond to queries about proper study implementation and management, address issues in a timely

manner, and assure consistent documentation, and effective information sharing. Rates of accrual, retention, and other parameters relevant to the CRC's performance will be regularly and closely monitored by the study team as well as the SRB.

17.5 Study Monitoring

On-site monitoring will be conducted to ensure that the study is conducted in compliance with human subjects protection and other research regulations and guidelines, recorded and reported in accordance with the protocol, is consistent with locally-accepted HIV counselling practices, standard operating procedures, Good Clinical Practice (GCP) and applicable regulatory requirements.

The monitor will confirm the quality and accuracy of data at the CRC by validation of CRFs against the source documents such as clinical records. The Investigators and volunteers, by giving consent, agree that the monitor may inspect study facilities and source records (e.g. informed consent forms, clinic and laboratory records, other source documents) as well as observe the performance of study procedures. Such information will be treated as strictly confidential and will under no circumstances be made publicly available.

The monitoring will adhere to Good Clinical Practice guidelines. The Principal Investigator will permit inspection of the facilities and all study-related documentation by authorized representatives of IAVI, and Government and Regulatory Authorities relevant to this study.

17.6 Investigator's Records

Study records include administrative documentation—including reports and correspondence relating to the study—as well as documentation related to each volunteer screened and/or enrolled in the study—including informed consent forms, case report forms, and all other source documents. The investigator will maintain and store, in a secure manner, complete, accurate, and current study records for a minimum of 2 years after marketing application approval or the study is discontinued and applicable national and local health authorities are notified. IAVI will notify the Principal Investigator of these events.

18.0 INDEMNITY

The Sponsor and Institution are responsible to have appropriate liability insurance. For research-related injuries and/or medical problems determined to result from receiving the Investigational Product, treatment including necessary emergency treatment and proper follow-up care will be made available to the volunteer free of charge at the expense of the Sponsor.

19.0 PUBLICATION

A primary manuscript describing safety and immune responses in this trial will be prepared promptly after the data analysis is available, based on the data compiled by the IAVI statistical centre. Authors will be representatives of each CRC, the statistical centre, the laboratories, IAVI and GSK, subject to the generally accepted criteria of contributions to the design, work, analysis

and writing of the study. Precedence will be given to authors from the CRC enrolling the greatest number of volunteers. Manuscripts will be reviewed by representatives of each participating group as specified in the CTA or VDP Agreement.

20.0 ETHICAL CONSIDERATIONS

The Principal Investigator will ensure that the study is conducted in compliance with the protocol, Standard Operating Procedures in accordance with guidelines laid down by the International Conference on Harmonisation for Good Clinical Practice in clinical studies, the ethical principles that have their origins in the Declaration of Helsinki and applicable regulatory requirements.

In addition to IEC/IRB and regulatory approvals, all other required approvals will be obtained before recruitment of volunteers.

APPENDIX A: SCHEDULE OF PROCEDURES—GROUPS A and B

Visits for Group	Groups A and B																	
**Month**	Screen	M0 Enrol				M1				M2	M4				M5	M9	M12	M16/ ET[1]
**Week**		0		W1	W2	W4	W4+ D3	W5	W6	W8	W16	W16 +D3	W17	W18	W20	W36	W48	W64
**Study Day**		0	D3	D7	D14	D28	D31	D35	D42	D56	D112	D115	D119	D126	D140	D252	D336	D448
**Visit Windows (Days)**		-42[2]	± 1	± 2	± 3	± 3	± 1	± 2	± 3	± 3	± 3	± 1	± 2	± 3	± 3	± 7	± 7	± 7
**Investigational Product**																		
Investigational Regimen A/B or Placebo		X				X					X							
**Consent /Questionnaires/Counseling**																		
Informed Consent and Assessment of Understanding	X																	
Screening Questionnaire	X																	
HIV Risk Assessment[16]	X	X													X			X
Social Impact Questionnaire																		X
HIV Risk Reduction Counseling	X	X				X					X				X	X	X	X
Pre-/Post HIV-test Counseling	X	X				X					X				X	X	X	X
Family Planning Counseling	X	X				X					X				X	X	X	X
**Clinical Safety Assessments**																		
Comprehensive Medical History	X																	
Interim Medical History		X	X	X	X	X	X	X	X	X	X	X	X	X	X	X	X	X
Concomitant Medications	X	X	X	X	X	X	X	X	X	X	X	X	X	X	X	X	X	X
General Physical Exam	X																	X
Symptom-Directed Physical Exam		X	X	X	X	X	X	X	X	X	X	X	X	X	X	X	X	
Weight & Height	X																	X
Ophthalmic examination[3]	X																	X

Visits for Group	Groups A and B																	
**Month**	Screen	M0 Enrol				M1				M2	M4				M5	M9	M12	M16/ ET[1]
**Week**		0		W1	W2	W4	W4+ D3	W5	W6	W8	W16	W16 +D3	W17	W18	W20	W36	W48	W64
**Study Day**		0	D3	D7	D14	D28	D31	D35	D42	D56	D112	D115	D119	D126	D140	D252	D336	D448
**Visit Windows (Days)**		-42[2]	± 1	± 2	± 3	± 3	± 1	± 2	± 3	± 3	± 3	± 1	± 2	± 3	± 3	± 7	± 7	± 7
**Investigational Product**																		
Investigational Regimen A/B or Placebo		X				X					X							
**Clinical Safety Assessments cont'd**																		
Vital Signs	X	X[4]				X[4]					X[4]							
Local and Systemic Reactogenicity		X[4]	X	X	X	X[4]	X	X	X		X[4]	X	X	X				
Adverse Events		X	X	X	X	X	X	X	X	X	X	X	X	X	X	X		
Serious Adverse Events	X[5]	X	X	X	X	X	X	X	X	X	X	X	X	X	X	X	X	X
**Lab Samples**																		
HIV test [6]	X	X				X					X				X	X	X	X
Hep BsAg, anti-HepC, active syphilis and active tuberculosis[7](Chest x-ray and sputum)	X																	
Haematology (+ plasma storage[8])	X	X		X		X		X		X	X		X		X	X		X
Clinical Chemistry (+ serum storage[8])	X	X		X		X		X		X	X		X		X	X		X
Immunology (CD4, CD8)		X																X
Urinalysis	X	X			X	X			X		X			X				X
Pregnancy Test (all female volunteers)	X	X				X					X						X	X
**Immunology Lab**																		
Ad35 neutralization antibody assay (anti-vector)[2, 9]	X	X													X			X
Sample HLA Typing[10]		X																

Visits for Group	Groups A and B																	
**Month**	**Screen**	**M0 Enrol**				**M1**				**M2**	**M4**				**M5**	**M9**	**M12**	**M16/ ET[1]**
**Week**		**0**		**W1**	**W2**	**W4**	**W4+ D3**	**W5**	**W6**	**W8**	**W16**	**W16 +D3**	**W17**	**W18**	**W20**	**W36**	**W48**	**W64**
**Study Day**		**0**	**D3**	**D7**	**D14**	**D28**	**D31**	**D35**	**D42**	**D56**	**D112**	**D115**	**D119**	**D126**	**D140**	**D252**	**D336**	**D448**
**Visit Windows (Days)**		**-42[2]**	**± 1**	**± 2**	**± 3**	**± 3**	**± 1**	**± 2**	**± 3**	**± 3**	**± 3**	**± 1**	**± 2**	**± 3**	**± 3**	**± 7**	**± 7**	**± 7**
**Investigational Product**																		
Investigational Regimen A/B or Placebo		X				X					X							
**Immunology Lab cont'd**																		
PBMCs for Cellular immunogenicity assays, QA[15]+ PBMC storage[8]	X[15]																	
- ELISPOT[11]		X								X	X				X			X
- ICS[12]		X							X	X	X				X			X
Exploratory cellular immunology assays[13]		X								X	X				X			X
Antibody Immunogenicity Assays (anti-HIV)[14]		X								X	X				X			X
**Other**																		
Refer to IAVI Long Term Follow -Up Protocol																		X

1 = Early Termination

2 = Allowable window for screening for Ad35 antibodies and collection of PBMCs from first 10 volunteers screened is -90 days; all other screening procedures is -42 days.

3 = Exam will be performed on 50% of enrolled volunteers at specified centres.First exam must be performed prior to first vaccination. Second exam must be performed within 1 month after final study visit.

4 = At baseline and 30 min post vaccination

5 = Only serious events that are related to study procedures will be collected during screening period

6 = If HIV infected, perform Early Termination visit and refer volunteer to IAVI Protocol H

7 = Chest X-ray and sputum examination for Acid Fast Bacilli will be performed for volunteers presenting with productive cough of more than 15 days duration to rule out active tuberculosis

[8] = Further details are in the Laboratory Analytical Plan. Stored serum and plasma will be split between IAVI Human Immunology Laboratory (HIL) and GSK Laboratory. Stored PBMC will be split between IAVI HIL and GSK for exploratory research objectives on cells.

[9] = Testing will be conducted at IAVI HIL

[10] =Testing will be contracted out to a qualified HLA typing laboratory and performed in volunteers with robust immune responses in order to map T cell epitopes

[11] =Immunogenicity testing (ELISPOT) will be conducted at CRCs, where feasible, and at IAVI HIL

[12] = Immunogenicity testing (ICS) will be conducted at GSK laboratories

[13] = Immunogenicity testing (Exploratory cellular immunology) will be conducted at CRCs, where feasible, and at IAVI HIL or at GSK laboratories

[14] = Serology against clade B antigens will be done at GSK laboratories. Serology to multiple clades will be performed at sites on a subset of subjects/time-points

[15] =PBMCs will be collected from first 10 volunteers screened at each clinical research centre for purposes of standardization, quality control, and future assays related to HIV vaccine research and development

[16] =HIV risk assessment at screening will determine eligibility. A separate HIV risk assessment questionnaire will be administered after enrolment.

APPENDIX B: SCHEDULE OF PROCEDURES—GROUP C

Visits for Group	Group C																	
**Month**	**Screen**	**M0 Enrol**				**M1**	**M3**				**M4**				**M5**	**M9**	**M12**	**M16/ ET[1]**
**Week**		**0**		**W1**	**W2**	**W4**	**W12**	**W12+ D3**	**W13**	**W14**	**W16**	**W16 +D3**	**W17**	**W18**	**W20**	**W36**	**W48**	**W64**
**Study Day**		**0**	**D3**	**D7**	**D14**	**D28**	**D84**	**D87**	**D91**	**D98**	**D112**	**D115**	**D119**	**D126**	**D140**	**D252**	**D336**	**D448**
**Visit Windows (Days)**		**-42[2]**	**± 1**	**± 2**	**± 3**	**± 3**	**± 3**	**± 1**	**± 2**	**± 3**	**± 3**	**± 1**	**± 2**	**± 3**	**± 3**	**± 7**	**± 7**	**± 7**
**Investigational Product**																		
Investigational Regimen C or Placebo		X					X				X							
**Consent /Questionnaires/Counseling**																		
Informed Consent and Assessment of Understanding	X																	
Screening Questionnaire	X																	
HIV Risk Assessment[16]	X	X													X			X
Social Impact Questionnaire																		X
HIV Risk Reduction Counseling	X	X					X				X				X	X	X	X
Pre-/Post HIV-test Counseling	X	X					X				X				X	X	X	X
Family Planning Counseling	X	X					X				X				X	X	X	X
**Clinical Safety Assessments**																		
Comprehensive Medical History	X																	
Interim Medical History		X	X	X	X	X	X	X	X	X	X	X	X	X	X	X	X	X
Concomitant Medications	X	X	X	X	X	X	X	X	X	X	X	X	X	X	X	X	X	X
General Physical Exam	X																	X
Symptom-Directed Physical Exam		X	X	X	X	X	X	X	X	X	X	X	X	X	X	X	X	
Weight & Height	X																	X
Ophthalmic examination[3]	X																	X
Vital Signs	X	X[4]					X[4]				X[4]							
Local and Systemic Reactogenicity		X[4]	X	X	X		X[4]	X	X	X	X[4]	X	X	X				

Visits for Group	Group C																	
**Month**	Screen	M0 Enrol				M1	M3				M4				M5	M9	M12	M16/ ET[1]
**Week**		0		W1	W2	W4	W12	W12+ D3	W13	W14	W16	W16 +D3	W17	W18	W20	W36	W48	W64
**Study Day**		0	D3	D7	D14	D28	D84	D87	D91	D98	D112	D115	D119	D126	D140	D252	D336	D448
**Visit Windows (Days)**		-42[2]	± 1	± 2	± 3	± 3	± 3	± 1	± 2	± 3	± 3	± 1	± 2	± 3	± 3	± 7	± 7	± 7
**Investigational Product**																		
Investigational Regimen C or Placebo		X					X				X							
**Clinical Safety Assessments cont'd**																		
Adverse Events		X	X	X	X	X	X	X	X	X	X	X	X	X	X	X		
Serious Adverse Events	X[5]	X	X	X	X	X	X	X	X	X	X	X	X	X	X	X	X	X
**Lab Samples**																		
HIV test [6]	X	X					X				X				X	X	X	X
Hep BsAg, anti-HepC, active syphilis and active tuberculosis[7](Chest x-ray and sputum)	X																	
Haematology (+ plasma storage[8])	X	X		X		X	X		X		X		X		X	X		X
Clinical Chemistry (+ serum storage[8])	X	X		X		X	X		X		X		X		X	X		X
Immunology (CD4, CD8)		X																X
Urinalysis	X	X			X		X			X	X			X				X
Pregnancy Test (all female volunteers)	X	X					X				X						X	X
**Immunology Lab**																		
Ad35 neutralization antibody assay (anti-vector)[2, 9]	X	X				X	X								X			X
Sample HLA Typing[10]		X																

Visits for Group	Group C																	
**Month**	Screen	M0 Enrol				M1	M3				M4				M5	M9	M12	M16/ ET[1]
**Week**		0		W1	W2	W4	W12	W12+ D3	W13	W14	W16	W16 +D3	W17	W18	W20	W36	W48	W64
**Study Day**		0	D3	D7	D14	D28	D84	D87	D91	D98	D112	D115	D119	D126	D140	D252	D336	D448
**Visit Windows (Days)**		-42[2]	± 1	± 2	± 3	± 3	± 3	± 1	± 2	± 3	± 3	± 1	± 2	± 3	± 3	± 7	± 7	± 7
**Investigational Product**																		
Investigational Regimen C or Placebo		X					X				X							
**Immunology Lab cont'd**																		
PBMCs for Cellular immunogenicity assays, QA[15]+ PBMC storage[8]	X																	
- ELISPOT[11]		X				X	X								X			X
- ICS[12]		X				X	X								X			X
Exploratory cellular immunology assays[13]		X				X	X								X			X
Antibody Immunogenicity Assays (anti-HIV)[14]		X				X	X								X			X
**Other**																		
Refer to IAVI Long Term Follow - Up Protocol																		X

1 = Early Termination
2 = Allowable window for screening for Ad35 antibodies and collection of PBMCs from first 10 volunteers screened is -90 days; all other screening procedures is -42 days.
3 = Exam will be performed on 50% of enrolled volunteers at specified centres. First exam must be performed prior to first vaccination. Second exam must be performed within 1 month after final study visit.
4 = At baseline and 30 min post vaccination
5 = Only serious events that are related to study procedures will be collected during screening period
6 = If HIV infected, perform Early Termination visit and refer volunteer to IAVI Protocol H

[7] = Chest X-ray and sputum examination for Acid Fast Bacilli will be performed for volunteers presenting with productive cough of more than 15 days duration to rule out active tuberculosis

[8] = Further details are in the Laboratory Analytical Plan. Stored serum and plasma will be split between IAVI Human Immunology Laboratory (HIL) and GSK Laboratory. Stored PBMC will be split between IAVI HIL and GSK for exploratory research objectives on cells.

[9] = Testing will be conducted at IAVI HIL

[10] =Testing will be contracted out to a qualified HLA typing laboratory and performed in volunteers with robust immune responses in order to map T cell epitopes

[11] =Immunogenicity testing (ELISPOT) will be conducted at CRCs, where feasible, and at IAVI HIL

[12] = Immunogenicity testing (ICS) will be conducted at GSK laboratories

[13] = Immunogenicity testing (Exploratory cellular immunology) will be conducted at CRCs, where feasible, and at IAVI HIL or at GSK laboratories

[14] = Serology against clade B antigens will be done at GSK laboratories. Serology to multiple clades will be performed at sites on a subset of subjects/time-points

[15] =PBMCs will be collected from first 10 volunteers screened at each clinical research centre for purposes of standardization, quality control, and future assays related to HIV vaccine research and development

[16] =HIV risk assessment at screening will determine eligibility. A separate HIV risk assessment questionnaire will be administered after enrolment.

APPENDIX C: SCHEDULE OF PROCEDURES—GROUP D

Visits for Group	Group D																	
**Month**	Screen	M0 Enrol				M1				M2	M4				M5	M9	M12	M16/ ET[1]
**Week**		0		W1	W2	W4	W4+ D3	W5	W6	W8	W16	W16 +D3	W17	W18	W20	W36	W48	W64
**Study Day**		0	D3	D7	D14	D28	D31	D35	D42	D56	D112	D115	D119	D126	D140	D252	D336	D448
**Visit Windows (Days)**		-42[2]	± 1	± 2	± 3	± 3	± 1	± 2	± 3	± 3	± 3	± 1	± 2	± 3	± 3	± 7	± 7	± 7
**Investigational Product**																		
Investigational Regimen D or Placebo		X				X					X							
**Consent /Questionnaires/Counseling**																		
Informed Consent and Assessment of Understanding	X																	
Screening Questionnaire	X																	
HIV Risk Assessment[16]	X	X													X			X
Social Impact Questionnaire																		X
HIV Risk Reduction Counseling	X	X				X					X				X	X	X	X
Pre-/Post HIV-test Counseling	X	X				X					X				X	X	X	X
Family Planning Counseling	X	X				X					X				X	X	X	X
**Clinical Safety Assessments**																		
Comprehensive Medical History	X																	
Interim Medical History		X	X	X	X	X	X	X	X	X	X	X	X	X	X	X	X	X
Concomitant Medications	X	X	X	X	X	X	X	X	X	X	X	X	X	X	X	X	X	X
General Physical Exam	X																	X
Symptom-Directed Physical Exam		X	X	X	X	X	X	X	X	X	X	X	X	X	X	X	X	
Weight & Height	X																	X
Ophthalmic examination[3]	X																	X

Visits for Group	Group D																	
**Month**	Screen	M0 Enrol				M1				M2	M4				M5	M9	M12	M16/ ET[1]
**Week**		0		W1	W2	W4	W4+ D3	W5	W6	W8	W16	W16 +D3	W17	W18	W20	W36	W48	W64
**Study Day**		0	D3	D7	D14	D28	D31	D35	D42	D56	D112	D115	D119	D126	D140	D252	D336	D448
**Visit Windows (Days)**		-42[2]	± 1	± 2	± 3	± 3	± 1	± 2	± 3	± 3	± 3	± 1	± 2	± 3	± 3	± 7	± 7	± 7
**Investigational Product**																		
Investigational Regimen D or Placebo		X				X					X							
**Clinical Safety Assessments cont'd**																		
Vital Signs	X	X[4]				X[4]					X[4]							
Local and Systemic Reactogenicity		X[4]	X	X	X	X[4]	X	X	X		X[4]	X	X	X				
Adverse Events		X	X	X	X	X	X	X	X	X	X	X	X	X	X	X		
Serious Adverse Events	X[5]	X	X	X	X	X	X	X	X	X	X	X	X	X	X	X	X	X
**Lab Samples**																		
HIV test [6]	X	X				X					X				X	X	X	X
Hep BsAg, anti-HepC, active syphilis and active tuberculosis[7](Chest x-ray and sputum)	X																	
Haematology (+ plasma storage[8])	X	X		X		X		X		X	X		X		X	X		X
Clinical Chemistry (+ serum storage[8])	X	X		X		X		X		X	X		X		X	X		X
Immunology (CD4, CD8)		X																X
Urinalysis	X	X			X	X			X		X			X				X
Pregnancy Test (all female volunteers)	X	X				X					X						X	X
**Immunology Lab**																		
Ad35 neutralization antibody assay (anti-vector)[2, 9]	X	X				X				X	X				X			X
Sample HLA Typing[10]		X																

Visits for Group	Group D																	
**Month**	Screen	M0 Enrol				M1				M2	M4				M5	M9	M12	M16/ ET[1]
**Week**		0		W1	W2	W4	W4+ D3	W5	W6	W8	W16	W16 +D3	W17	W18	W20	W36	W48	W64
**Study Day**		0	D3	D7	D14	D28	D31	D35	D42	D56	D112	D115	D119	D126	D140	D252	D336	D448
**Visit Windows (Days)**		-42[2]	± 1	± 2	± 3	± 3	± 1	± 2	± 3	± 3	± 3	± 1	± 2	± 3	± 3	± 7	± 7	± 7
**Investigational Product**																		
Investigational Regimen D or Placebo		X				X					X							
**Immunology Lab cont'd**																		
PBMCs for Cellular immunogenicity assays, QA[15]+ PBMC storage[8]	X[15]																	
- ELISPOT[11]		X				X				X	X				X			X
- ICS[12]		X				X				X	X				X			X
Exploratory cellular immunology assays[13]		X				X				X	X				X			X
Antibody Immunogenicity Assays (anti-HIV)[14]		X				X				X	X				X			X
**Other**																		
Refer to IAVI Long Term Follow -Up Protocol																		X

1 = Early Termination
2 = Allowable window for screening for Ad35 antibodies and collection of PBMCs from first 10 volunteers screened is -90 days; all other screening procedures is -42 days.
3 = Exam will be performed on 50% of enrolled volunteers at specified centres. First exam must be performed prior to first vaccination. Second exam must be performed within 1 month after final study visit.
4 = At baseline and 30 min post vaccination
5 = Only serious events that are related to study procedures will be collected during screening period
6 = If HIV infected, perform Early Termination visit and refer volunteer to IAVI Protocol H

7 = Chest X-ray and sputum examination for Acid Fast Bacilli will be performed for volunteers presenting with productive cough of more than 15 days duration to rule out active tuberculosis

8 = Further details are in the Laboratory Analytical Plan. Stored serum and plasma will be split between IAVI Human Immunology Laboratory (HIL) and GSK Laboratory. Stored PBMC will be split between IAVI HIL and GSK for exploratory research objectives on cells.

9 = Testing will be conducted at IAVI HIL

10 =Testing will be contracted out to a qualified HLA typing laboratory and performed in volunteers with robust immune responses in order to map T cell epitopes

11 =Immunogenicity testing (ELISPOT) will be conducted at CRCs, where feasible, and at IAVI HIL

12 = Immunogenicity testing (ICS) will be conducted at GSK laboratories

13 = Immunogenicity testing (Exploratory cellular immunology) will be conducted at CRCs, where feasible, and at IAVI HIL or at GSK laboratories

14 = Serology against clade B antigens will be done at GSK laboratories. Serology to multiple clades will be performed at sites on a subset of subjects/time-points

15 =PBMCs will be collected from first 10 volunteers screened at each clinical research centre for purposes of standardization, quality control, and future assays related to HIV vaccine research and development

16 =HIV risk assessment at screening will determine eligibility. A separate HIV risk assessment questionnaire will be administered after enrolment.

APPENDIX D - ADVERSE EVENT SEVERITY ASSESSMENT TABLE
VERSION 1.0 DECEMBER 2004; CLARIFICATION AUGUST 2009
ADAPTED FROM: DIVISION OF AIDS TABLE FOR GRADING THE SEVERITY OF ADULT ADVERSE EVENTS

Quick Reference

The Division of AIDS Table for Grading the Severity of Adult and Pediatric Adverse Events ("DAIDS AE grading table") is a descriptive terminology which can be utilized for Adverse Event (AE) reporting. A grading (severity) scale is provided for each AE term.

General Instructions

Estimating Severity Grade

If the need arises to grade a clinical AE that is not identified in the DAIDS AE grading table, use the category "Estimating Severity Grade" located at the top of Page 3. For AEs that are not listed in the table but will be collected systematically for a study/trial, protocol teams are highly encouraged to define study-specific severity scales within the protocol or an appendix to the protocol. (Please see "Template Wording for the Expedited Adverse Event Reporting Section of DAIDS-sponsored Protocols".) This is particularly important for laboratory values because the "Estimating Severity Grade" category only applies to clinical symptoms.

Grading Adult and Pediatric AEs

The DAIDS AE grading table includes parameters for grading both Adult and Pediatric AEs. When a single set of parameters is not appropriate for grading specific types of AEs for both Adult and Pediatric populations, separate sets of parameters for Adult and/or Pediatric populations (with specified respective age ranges) are given in the table. If there is no distinction in the table between Adult and Pediatric values for a type of AE, then the single set of parameters listed is to be used for grading the severity of both Adult and Pediatric events of that type.

Determining Severity Grade

If the severity of an AE could fall under either one of two grades (e.g., the severity of an AE could be either Grade 2 or Grade 3), select the higher of the two grades for the AE.

Definitions

Basic Self-care Functions	Adult<br>Activities such as bathing, dressing, toileting, transfer/movement, continence, and feeding.<br>Young Children<br>Activities that are age and culturally appropriate (e.g., feeding self with culturally appropriate eating implement).
LLN	Lower limit of normal
Medical Intervention	Use of pharmacologic or biologic agent(s) for treatment of an AE.
NA	Not Applicable
Operative Intervention	Surgical OR other invasive mechanical procedures.
ULN	Upper limit of normal
Usual Social & Functional Activities	Adult<br>Adaptive tasks and desirable activities, such as going to work, shopping, cooking, use of transportation, pursuing a hobby, etc.<br>Young Children<br>Activities that are age and culturally appropriate (e.g., social interactions, play activities, learning tasks, etc.).

PARAMETER	GRADE 1 MILD	GRADE 2 MODERATE	GRADE 3 SEVERE	GRADE 4 VERY SEVERE
**ESTIMATING SEVERITY GRADE**				
Clinical adverse event NOT identified elsewhere in this DAIDS AE Grading Table	Symptoms causing no or minimal interference with usual social & functional activities	Symptoms causing greater than minimal interference with usual social & functional activities	Symptoms causing inability to perform usual social & functional activities	Symptoms causing inability to perform basic self-care functions OR Medical or operative intervention indicated to prevent permanent impairment, persistent disability, or death
**SYSTEMIC**				
Acute systemic allergic reaction	Localized urticaria (wheals) with no medical intervention indicated	Localized urticaria with medical intervention indicated OR Mild angioedema with no medical intervention indicated	Generalized urticaria OR Angioedema with medical intervention indicated OR Symptomatic mild bronchospasm	Acute anaphylaxis OR Life-threatening bronchospasm OR laryngeal edema
Chills	Symptoms causing no or minimal interference with usual social & functional activities	Symptoms causing greater than minimal interference with usual social & functional activities	Symptoms causing inability to perform usual social & functional activities	NA
Fatigue<br>Malaise	Symptoms causing no or minimal interference with usual social & functional activities	Symptoms causing greater than minimal interference with usual social & functional activities	Symptoms causing inability to perform usual social & functional activities	Incapacitating fatigue/ malaise symptoms causing inability to perform basic self-care functions
Fever (nonaxillary)	37.7 – 38.6°C	38.7 – 39.3°C	39.4 – 40.5°C	> 40.5°C
Pain (indicate body site)<br>DO NOT use for pain due to injection (See Injection Site Reactions: Injection site pain)<br>See also Headache, Arthralgia, and Myalgia	Pain causing no or minimal interference with usual social & functional activities	Pain causing greater than minimal interference with usual social & functional activities	Pain causing inability to perform usual social & functional activities	Disabling pain causing inability to perform basic self-care functions OR Hospitalization (other than emergency room visit) indicated

Basic Self-care Functions – Adult: Activities such as bathing, dressing, toileting, transfer/movement, continence, and feeding.

Basic Self-care Functions – Young Children: Activities that are age and culturally appropriate (e.g., feeding self with culturally appropriate eating implement).

Usual Social & Functional Activities – Adult: Adaptive tasks and desirable activities, such as going to work, shopping, cooking, use of transportation, pursuing a hobby, etc.

Usual Social & Functional Activities – Young Children: Activities that are age and culturally appropriate (e.g., social interactions, play activities, learning tasks, etc.).

PARAMETER	GRADE 1<br>MILD	GRADE 2<br>MODERATE	GRADE 3<br>SEVERE	GRADE 4<br>VERY SEVERE
Unintentional weight loss	NA	5 – 9% loss in body weight from baseline	10 – 19% loss in body weight from baseline	≥ 20% loss in body weight from baseline OR Aggressive intervention indicated [e.g., tube feeding or total parenteral nutrition (TPN)]
**INFECTION**				
Infection (any other than HIV infection)	Localized, no systemic antimicrobial treatment indicated AND Symptoms causing no or minimal interference with usual social & functional activities	Systemic antimicrobial treatment indicated OR Symptoms causing greater than minimal interference with usual social & functional activities	Systemic antimicrobial treatment indicated AND Symptoms causing inability to perform usual social & functional activities OR Operative intervention (other than simple incision and drainage) indicated	Life-threatening consequences (e.g., septic shock)
**INJECTION SITE REACTIONS**				
Injection site pain (pain without touching)<br>Or<br>Tenderness (pain when area is touched)	Pain/tenderness causing no or minimal limitation of use of limb	Pain/tenderness limiting use of limb OR Pain/tenderness causing greater than minimal interference with usual social & functional activities	Pain/tenderness causing inability to perform usual social & functional activities	Pain/tenderness causing inability to perform basic self-care function OR Hospitalization (other than emergency room visit) indicated for management of pain/tenderness
Injection site reaction (localized)				
**Adult > 15 years**	Erythema OR Induration of 5x5 cm – 9x9 cm (or 25 cm^2 – 81cm^2)	Erythema OR Induration OR Edema > 9 cm any diameter (or > 81 cm^2)	Ulceration OR Secondary infection OR Phlebitis OR Sterile abscess OR Drainage	Necrosis (involving dermis and deeper tissue)
**Pediatric ≤ 15 years**	Erythema OR Induration OR Edema present but ≤ 2.5 cm diameter	Erythema OR Induration OR Edema > 2.5 cm diameter but < 50% surface area of the extremity segment (e.g., upper arm/thigh)	Erythema OR Induration OR Edema involving ≥ 50% surface area of the extremity segment (e.g., upper arm/thigh) OR Ulceration OR Secondary infection OR Phlebitis OR Sterile abscess OR Drainage	Necrosis (involving dermis and deeper tissue)

Basic Self-care Functions – Adult: Activities such as bathing, dressing, toileting, transfer/movement, continence, and feeding.

Basic Self-care Functions – Young Children: Activities that are age and culturally appropriate (e.g., feeding self with culturally appropriate eating implement).

Usual Social & Functional Activities – Adult: Adaptive tasks and desirable activities, such as going to work, shopping, cooking, use of transportation, pursuing a hobby, etc.

Usual Social & Functional Activities – Young Children: Activities that are age and culturally appropriate (e.g., social interactions, play activities, learning tasks, etc.).

PARAMETER	GRADE 1<br>MILD	GRADE 2<br>MODERATE	GRADE 3<br>SEVERE	GRADE 4<br>VERY SEVERE
Pruritis associated with injection<br>See also Skin: Pruritis (itching - no skin lesions)	Itching localized to injection site AND Relieved spontaneously or with < 48 hours treatment	Itching beyond the injection site but not generalized OR Itching localized to injection site requiring ≥ 48 hours treatment	Generalized itching causing inability to perform usual social & functional activities	NA
**SKIN – DERMATOLOGICAL**				
Alopecia	Thinning detectable by study participant (or by caregiver for young children and disabled adults)	Thinning or patchy hair loss detectable by health care provider	Complete hair loss	NA
Cutaneous reaction – rash	Localized macular rash	Diffuse macular, maculopapular, or morbilliform rash OR Target lesions	Diffuse macular, maculopapular, or morbilliform rash with vesicles or limited number of bullae OR Superficial ulcerations of mucous membrane limited to one site	Extensive or generalized bullous lesions OR Stevens-Johnson syndrome OR Ulceration of mucous membrane involving two or more distinct mucosal sites OR Toxic epidermal necrolysis (TEN)
Hyperpigmentation	Slight or localized	Marked or generalized	NA	NA
Hypopigmentation	Slight or localized	Marked or generalized	NA	NA
Pruritis (itching – no skin lesions)<br>(See also Injection Site Reactions: Pruritis associated with injection)	Itching causing no or minimal interference with usual social & functional activities	Itching causing greater than minimal interference with usual social & functional activities	Itching causing inability to perform usual social & functional activities	NA
**CARDIOVASCULAR**				
Cardiac arrhythmia (general)<br>(By ECG or physical exam)	Asymptomatic AND No intervention indicated	Asymptomatic AND Non-urgent medical intervention indicated	Symptomatic, non-life-threatening AND Non-urgent medical intervention indicated	Life-threatening arrhythmia OR Urgent intervention indicated
Cardiac-ischemia/infarction	NA	NA	Symptomatic ischemia (stable angina) OR Testing consistent with ischemia	Unstable angina OR Acute myocardial infarction

Basic Self-care Functions – Adult: Activities such as bathing, dressing, toileting, transfer/movement, continence, and feeding.

Basic Self-care Functions – Young Children: Activities that are age and culturally appropriate (e.g., feeding self with culturally appropriate eating implement).

Usual Social & Functional Activities – Adult: Adaptive tasks and desirable activities, such as going to work, shopping, cooking, use of transportation, pursuing a hobby, etc.

Usual Social & Functional Activities – Young Children: Activities that are age and culturally appropriate (e.g., social interactions, play activities, learning tasks, etc.).

PARAMETER		GRADE 1 MILD	GRADE 2 MODERATE	GRADE 3 SEVERE	GRADE 4 VERY SEVERE
Hemorrhage (significant acute blood loss)		NA	Symptomatic AND No transfusion indicated	Symptomatic AND Transfusion of ≤ 2 units packed RBCs (for children ≤ 10 cc/kg) indicated	Life-threatening hypotension OR Transfusion of > 2 units packed RBCs (for children > 10 cc/kg) indicated
Hypertension					
	**Adult > 17 years** (with repeat testing at same visit)	140 – 159 mmHg systolic OR 90 – 99 mmHg diastolic	160 – 179 mmHg systolic OR 100 – 109 mmHg diastolic	≥ 180 mmHg systolic OR ≥ 110 mmHg diastolic	Life-threatening consequences (e.g., malignant hypertension) OR Hospitalization indicated (other than emergency room visit)
	**Correction**: in Grade 2 to 160 - 179 from > 160-179 (systolic) and to ≥ 100 -109 from > 100-109 (diastolic) and in Grade 3 to **≥** 180 from > 180 (systolic) and to **≥** 110 from > 110 (diastolic).				
	**Pediatric ≤ 17 years** (with repeat testing at same visit)	NA	91st – 94th percentile adjusted for age, height, and gender (systolic and/or diastolic)	≥ 95th percentile adjusted for age, height, and gender (systolic and/or diastolic)	Life-threatening consequences (e.g., malignant hypertension) OR Hospitalization indicated (other than emergency room visit)
Hypotension		NA	Symptomatic, corrected with oral fluid replacement	Symptomatic, IV fluids indicated	Shock requiring use of vasopressors or mechanical assistance to maintain blood pressure
Pericardial effusion		Asymptomatic, small effusion requiring no intervention	Asymptomatic, moderate or larger effusion requiring no intervention	Effusion with non-life threatening physiologic consequences OR Effusion with non-urgent intervention indicated	Life-threatening consequences (e.g., tamponade) OR Urgent intervention indicated
Prolonged PR interval					
	**Adult > 16 years**	PR interval 0.21 – 0.25 sec	PR interval > 0.25 sec	Type II 2nd degree AV block OR Ventricular pause > 3.0 sec	Complete AV block
	**Pediatric ≤ 16 years**	1st degree AV block (PR > normal for age and rate)	Type I 2nd degree AV block	Type II 2nd degree AV block	Complete AV block

Basic Self-care Functions – Adult: Activities such as bathing, dressing, toileting, transfer/movement, continence, and feeding.

Basic Self-care Functions – Young Children: Activities that are age and culturally appropriate (e.g., feeding self with culturally appropriate eating implement).

Usual Social & Functional Activities – Adult: Adaptive tasks and desirable activities, such as going to work, shopping, cooking, use of transportation, pursuing a hobby, etc.

Usual Social & Functional Activities – Young Children: Activities that are age and culturally appropriate (e.g., social interactions, play activities, learning tasks, etc.).

PARAMETER		GRADE 1 MILD	GRADE 2 MODERATE	GRADE 3 SEVERE	GRADE 4 VERY SEVERE
Prolonged QTc					
	**Adult > 16 years**	Asymptomatic, QTc interval 0.45 – 0.47 sec OR Increase interval < 0.03 sec above baseline	Asymptomatic, QTc interval 0.48 – 0.49 sec OR Increase in interval 0.03 – 0.05 sec above baseline	Asymptomatic, QTc interval ≥ 0.50 sec OR Increase in interval ≥ 0.06 sec above baseline	Life-threatening consequences, e.g. Torsade de pointes or other associated serious ventricular dysrhythmia
	**Pediatric ≤ 16 years**	Asymptomatic, QTc interval 0.450 – 0.464 sec	Asymptomatic, QTc interval 0.465 – 0.479 sec	Asymptomatic, QTc interval ≥ 0.480 sec	Life-threatening consequences, e.g. Torsade de pointes or other associated serious ventricular dysrhythmia
Thrombosis/embolism		NA	Deep vein thrombosis AND No intervention indicated (e.g., anticoagulation, lysis filter, invasive procedure)	Deep vein thrombosis AND Intervention indicated (e.g., anticoagulation, lysis filter, invasive procedure)	Embolic event (e.g., pulmonary embolism, life-threatening thrombus)
Vasovagal episode (associated with a procedure of any kind)		Present without loss of consciousness	Present with transient loss of consciousness	NA	NA
Ventricular dysfunction (congestive heart failure)		NA	Asymptomatic diagnostic finding AND intervention indicated	New onset with symptoms OR Worsening symptomatic congestive heart failure	Life-threatening congestive heart failure
**GASTROINTESTINAL**					
Anorexia		Loss of appetite without decreased oral intake	Loss of appetite associated with decreased oral intake without significant weight loss	Loss of appetite associated with significant weight loss	Life-threatening consequences OR Aggressive intervention indicated [e.g., tube feeding or total parenteral nutrition (TPN)]
**Comment:** Please note that, while the grading scale provided for Unintentional Weight Loss may be used as a guideline when grading anorexia, this is not a requirement and should not be used as a substitute for clinical judgment.					
Ascites		Asymptomatic	Symptomatic AND Intervention indicated (e.g., diuretics or therapeutic paracentesis)	Symptomatic despite intervention	Life-threatening consequences

Basic Self-care Functions – Adult: Activities such as bathing, dressing, toileting, transfer/movement, continence, and feeding.

Basic Self-care Functions – Young Children: Activities that are age and culturally appropriate (e.g., feeding self with culturally appropriate eating implement).

Usual Social & Functional Activities – Adult: Adaptive tasks and desirable activities, such as going to work, shopping, cooking, use of transportation, pursuing a hobby, etc.

Usual Social & Functional Activities – Young Children: Activities that are age and culturally appropriate (e.g., social interactions, play activities, learning tasks, etc.).

PARAMETER	GRADE 1 MILD	GRADE 2 MODERATE	GRADE 3 SEVERE	GRADE 4 VERY SEVERE
Cholecystitis	NA	Symptomatic AND Medical intervention indicated	Radiologic, endoscopic, or operative intervention indicated	Life-threatening consequences (e.g., sepsis or perforation)
Constipation	NA	Persistent constipation requiring regular use of dietary modifications, laxatives, or enemas	Obstipation with manual evacuation indicated	Life-threatening consequences (e.g., obstruction)
Diarrhea				
**Adult and Pediatric ≥ 1 year**	Transient or intermittent episodes of unformed stools OR Increase of ≤ 3 stools over baseline per 24-hour period	Persistent episodes of unformed to watery stools OR Increase of 4 – 6 stools over baseline per 24-hour period	Bloody diarrhea OR Increase of ≥ 7 stools per 24-hour period OR IV fluid replacement indicated	Life-threatening consequences (e.g., hypotensive shock)
**Pediatric < 1 year**	Liquid stools (more unformed than usual) but usual number of stools	Liquid stools with increased number of stools OR Mild dehydration	Liquid stools with moderate dehydration	Liquid stools resulting in severe dehydration with aggressive rehydration indicated OR Hypotensive shock
Dysphagia-Odynophagia	Symptomatic but able to eat usual diet	Symptoms causing altered dietary intake without medical intervention indicated	Symptoms causing severely altered dietary intake with medical intervention indicated	Life-threatening reduction in oral intake
Mucositis/stomatitis (clinical exam)<br>Indicate site (e.g., larynx, oral)<br>See Genitourinary for Vulvovaginitis<br>See also Dysphagia-Odynophagia and Proctitis	Erythema of the mucosa	Patchy pseudomembranes or ulcerations	Confluent pseudomembranes or ulcerations OR Mucosal bleeding with minor trauma	Tissue necrosis OR Diffuse spontaneous mucosal bleeding OR Life-threatening consequences (e.g., aspiration, choking)
Nausea	Transient (< 24 hours) or intermittent nausea with no or minimal interference with oral intake	Persistent nausea resulting in decreased oral intake for 24 – 48 hours	Persistent nausea resulting in minimal oral intake for > 48 hours OR Aggressive rehydration indicated (e.g., IV fluids)	Life-threatening consequences (e.g., hypotensive shock)
Pancreatitis	NA	Symptomatic AND Hospitalization not indicated (other than emergency room visit)	Symptomatic AND Hospitalization indicated (other than emergency room visit)	Life-threatening consequences (e.g., circulatory failure, hemorrhage, sepsis)

Basic Self-care Functions – Adult: Activities such as bathing, dressing, toileting, transfer/movement, continence, and feeding.

Basic Self-care Functions – Young Children: Activities that are age and culturally appropriate (e.g., feeding self with culturally appropriate eating implement).

Usual Social & Functional Activities – Adult: Adaptive tasks and desirable activities, such as going to work, shopping, cooking, use of transportation, pursuing a hobby, etc.

Usual Social & Functional Activities – Young Children: Activities that are age and culturally appropriate (e.g., social interactions, play activities, learning tasks, etc.).

PARAMETER	GRADE 1<br>MILD	GRADE 2<br>MODERATE	GRADE 3<br>SEVERE	GRADE 4<br>VERY SEVERE
Proctitis (functional-symptomatic)<br>Also see Mucositis/stomatitis for clinical exam	Rectal discomfort AND No intervention indicated	Symptoms causing greater than minimal interference with usual social & functional activities OR Medical intervention indicated	Symptoms causing inability to perform usual social & functional activities OR Operative intervention indicated	Life-threatening consequences (e.g., perforation)
Vomiting	Transient or intermittent vomiting with no or minimal interference with oral intake	Frequent episodes of vomiting with no or mild dehydration	Persistent vomiting resulting in orthostatic hypotension OR Aggressive rehydration indicated (e.g., IV fluids)	Life-threatening consequences (e.g., hypotensive shock)
**NEUROLOGIC**				
Alteration in personality-behavior or in mood (e.g., agitation, anxiety, depression, mania, psychosis)	Alteration causing no or minimal interference with usual social & functional activities	Alteration causing greater than minimal interference with usual social & functional activities	Alteration causing inability to perform usual social & functional activities	Behavior potentially harmful to self or others (e.g., suicidal and homicidal ideation or attempt, acute psychosis) OR Causing inability to perform basic self-care functions
Altered Mental Status<br>For Dementia, see Cognitive and behavioral/attentional disturbance (including dementia and attention deficit disorder)	Changes causing no or minimal interference with usual social & functional activities	Mild lethargy or somnolence causing greater than minimal interference with usual social & functional activities	Confusion, memory impairment, lethargy, or somnolence causing inability to perform usual social & functional activities	Delirium OR obtundation, OR coma
Ataxia	Asymptomatic ataxia detectable on exam OR Minimal ataxia causing no or minimal interference with usual social & functional activities	Symptomatic ataxia causing greater than minimal interference with usual social & functional activities	Symptomatic ataxia causing inability to perform usual social & functional activities	Disabling ataxia causing inability to perform basic self-care functions
Cognitive and behavioral/attentional disturbance (including dementia and attention deficit disorder)	Disability causing no or minimal interference with usual social & functional activities OR Specialized resources not indicated	Disability causing greater than minimal interference with usual social & functional activities OR Specialized resources on part-time basis indicated	Disability causing inability to perform usual social & functional activities OR Specialized resources on a full-time basis indicated	Disability causing inability to perform basic self-care functions OR Institutionalization indicated

Basic Self-care Functions – Adult: Activities such as bathing, dressing, toileting, transfer/movement, continence, and feeding.

Basic Self-care Functions – Young Children: Activities that are age and culturally appropriate (e.g., feeding self with culturally appropriate eating implement).

Usual Social & Functional Activities – Adult: Adaptive tasks and desirable activities, such as going to work, shopping, cooking, use of transportation, pursuing a hobby, etc.

Usual Social & Functional Activities – Young Children: Activities that are age and culturally appropriate (e.g., social interactions, play activities, learning tasks, etc.).

PARAMETER	GRADE 1 MILD	GRADE 2 MODERATE	GRADE 3 SEVERE	GRADE 4 VERY SEVERE
CNS ischemia (acute)	NA	NA	Transient ischemic attack	Cerebral vascular accident (CVA, stroke) with neurological deficit
Developmental delay – **Pediatric ≤ 16 years**	Mild developmental delay, either motor or cognitive, as determined by comparison with a developmental screening tool appropriate for the setting	Moderate developmental delay, either motor or cognitive, as determined by comparison with a developmental screening tool appropriate for the setting	Severe developmental delay, either motor or cognitive, as determined by comparison with a developmental screening tool appropriate for the setting	Developmental regression, either motor or cognitive, as determined by comparison with a developmental screening tool appropriate for the setting
Headache	Symptoms causing no or minimal interference with usual social & functional activities	Symptoms causing greater than minimal interference with usual social & functional activities	Symptoms causing inability to perform usual social & functional activities	Symptoms causing inability to perform basic self-care functions OR Hospitalization indicated (other than emergency room visit) OR Headache with significant impairment of alertness or other neurologic function
Insomnia	NA	Difficulty sleeping causing greater than minimal interference with usual social & functional activities	Difficulty sleeping causing inability to perform usual social & functional activities	Disabling insomnia causing inability to perform basic self-care functions
Neuromuscular weakness (including myopathy & neuropathy)	Asymptomatic with decreased strength on exam OR Minimal muscle weakness causing no or minimal interference with usual social & functional activities	Muscle weakness causing greater than minimal interference with usual social & functional activities	Muscle weakness causing inability to perform usual social & functional activities	Disabling muscle weakness causing inability to perform basic self-care functions OR Respiratory muscle weakness impairing ventilation
Neurosensory alteration (including paresthesia and painful neuropathy)	Asymptomatic with sensory alteration on exam or minimal paresthesia causing no or minimal interference with usual social & functional activities	Sensory alteration or paresthesia causing greater than minimal interference with usual social & functional activities	Sensory alteration or paresthesia causing inability to perform usual social & functional activities	Disabling sensory alteration or paresthesia causing inability to perform basic self-care functions

Basic Self-care Functions – Adult: Activities such as bathing, dressing, toileting, transfer/movement, continence, and feeding.

Basic Self-care Functions – Young Children: Activities that are age and culturally appropriate (e.g., feeding self with culturally appropriate eating implement).

Usual Social & Functional Activities – Adult: Adaptive tasks and desirable activities, such as going to work, shopping, cooking, use of transportation, pursuing a hobby, etc.

Usual Social & Functional Activities – Young Children: Activities that are age and culturally appropriate (e.g., social interactions, play activities, learning tasks, etc.).

PARAMETER	GRADE 1 MILD	GRADE 2 MODERATE	GRADE 3 SEVERE	GRADE 4 VERY SEVERE
Seizure: (new onset) **– Adult ≥ 18 years** See also Seizure: (known pre-existing seizure disorder)	NA	1 seizure	2 – 4 seizures	Seizures of any kind which are prolonged, repetitive (e.g., status epilepticus), or difficult to control (e.g., refractory epilepsy)
Seizure: (known pre-existing seizure disorder) **– Adult ≥ 18 years** For worsening of existing epilepsy the grades should be based on an increase from previous level of control to any of these levels.	NA	Increased frequency of pre-existing seizures (non-repetitive) without change in seizure character OR Infrequent break-through seizures while on stable medication in a previously controlled seizure disorder	Change in seizure character from baseline either in duration or quality (e.g., severity or focality)	Seizures of any kind which are prolonged, repetitive (e.g., status epilepticus), or difficult to control (e.g., refractory epilepsy)
Seizure **– Pediatric < 18 years**	Seizure, generalized onset with or without secondary generalization, lasting < 5 minutes with < 24 hours post ictal state	Seizure, generalized onset with or without secondary generalization, lasting 5 – 20 minutes with < 24 hours post ictal state	Seizure, generalized onset with or without secondary generalization, lasting > 20 minutes	Seizure, generalized onset with or without secondary generalization, requiring intubation and sedation
Syncope (not associated with a procedure)	NA	Present	NA	NA
Vertigo	Vertigo causing no or minimal interference with usual social & functional activities	Vertigo causing greater than minimal interference with usual social & functional activities	Vertigo causing inability to perform usual social & functional activities	Disabling vertigo causing inability to perform basic self-care functions
**RESPIRATORY**				
Bronchospasm (acute)	FEV1 or peak flow reduced to 70 – 80%	FEV1 or peak flow 50 – 69%	FEV1 or peak flow 25 – 49%	Cyanosis OR FEV1 or peak flow < 25% OR Intubation
Dyspnea or respiratory distress				
**Adult ≥ 14 years**	Dyspnea on exertion with no or minimal interference with usual social & functional activities	Dyspnea on exertion causing greater than minimal interference with usual social & functional activities	Dyspnea at rest causing inability to perform usual social & functional activities	Respiratory failure with ventilatory support indicated

Basic Self-care Functions – Adult: Activities such as bathing, dressing, toileting, transfer/movement, continence, and feeding.

Basic Self-care Functions – Young Children: Activities that are age and culturally appropriate (e.g., feeding self with culturally appropriate eating implement).

Usual Social & Functional Activities – Adult: Adaptive tasks and desirable activities, such as going to work, shopping, cooking, use of transportation, pursuing a hobby, etc.

Usual Social & Functional Activities – Young Children: Activities that are age and culturally appropriate (e.g., social interactions, play activities, learning tasks, etc.).

PARAMETER	GRADE 1 MILD	GRADE 2 MODERATE	GRADE 3 SEVERE	GRADE 4 VERY SEVERE
**Pediatric < 14 years**	Wheezing OR minimal increase in respiratory rate for age	Nasal flaring OR Intercostal retractions OR Pulse oximetry 90 – 95%	Dyspnea at rest causing inability to perform usual social & functional activities OR Pulse oximetry < 90%	Respiratory failure with ventilatory support indicated
**MUSCULOSKELETAL**				
Arthralgia<br>See also Arthritis	Joint pain causing no or minimal interference with usual social & functional activities	Joint pain causing greater than minimal interference with usual social & functional activities	Joint pain causing inability to perform usual social & functional activities	Disabling joint pain causing inability to perform basic self-care functions
Arthritis<br>See also Arthralgia	Stiffness or joint swelling causing no or minimal interference with usual social & functional activities	Stiffness or joint swelling causing greater than minimal interference with usual social & functional activities	Stiffness or joint swelling causing inability to perform usual social & functional activities	Disabling joint stiffness or swelling causing inability to perform basic self-care functions
Bone Mineral Loss				
**Adult ≥ 21 years**	BMD t-score -2.5 to -1.0	BMD t-score < -2.5	Pathological fracture (including loss of vertebral height)	Pathologic fracture causing life-threatening consequences
**Pediatric < 21 years**	BMD z-score -2.5 to -1.0	BMD z-score < -2.5	Pathological fracture (including loss of vertebral height)	Pathologic fracture causing life-threatening consequences
Myalgia<br>(non-injection site)	Muscle pain causing no or minimal interference with usual social & functional activities	Muscle pain causing greater than minimal interference with usual social & functional activities	Muscle pain causing inability to perform usual social & functional activities	Disabling muscle pain causing inability to perform basic self-care functions
Osteonecrosis	NA	Asymptomatic with radiographic findings AND No operative intervention indicated	Symptomatic bone pain with radiographic findings OR Operative intervention indicated	Disabling bone pain with radiographic findings causing inability to perform basic self-care functions

Basic Self-care Functions – Adult: Activities such as bathing, dressing, toileting, transfer/movement, continence, and feeding.

Basic Self-care Functions – Young Children: Activities that are age and culturally appropriate (e.g., feeding self with culturally appropriate eating implement).

Usual Social & Functional Activities – Adult: Adaptive tasks and desirable activities, such as going to work, shopping, cooking, use of transportation, pursuing a hobby, etc.

Usual Social & Functional Activities – Young Children: Activities that are age and culturally appropriate (e.g., social interactions, play activities, learning tasks, etc.).

PARAMETER	GRADE 1<br>MILD	GRADE 2<br>MODERATE	GRADE 3<br>SEVERE	GRADE 4<br>VERY SEVERE
**GENITOURINARY**				
Cervicitis (symptoms)<br>(For use in studies evaluating topical study agents)<br>For other cervicitis see Infection: Infection (any other than HIV infection)	Symptoms causing no or minimal interference with usual social & functional activities	Symptoms causing greater than minimal interference with usual social & functional activities	Symptoms causing inability to perform usual social & functional activities	Symptoms causing inability to perform basic self-care functions
Cervicitis (clinical exam)<br>(For use in studies evaluating topical study agents)<br>For other cervicitis see Infection: Infection (any other than HIV infection)	Minimal cervical abnormalities on examination (erythema, mucopurulent discharge, or friability) OR Epithelial disruption < 25% of total surface	Moderate cervical abnormalities on examination (erythema, mucopurulent discharge, or friability) OR Epithelial disruption of 25 – 49% total surface	Severe cervical abnormalities on examination (erythema, mucopurulent discharge, or friability) OR Epithelial disruption 50 – 75% total surface	Epithelial disruption > 75% total surface
Inter-menstrual bleeding (IMB)	Spotting observed by participant OR Minimal blood observed during clinical or colposcopic examination	Inter-menstrual bleeding not greater in duration or amount than usual menstrual cycle	Inter-menstrual bleeding greater in duration or amount than usual menstrual cycle	Hemorrhage with life-threatening hypotension OR Operative intervention indicated
Urinary tract obstruction (e.g., stone)	NA	Signs or symptoms of urinary tract obstruction without hydronephrosis or renal dysfunction	Signs or symptoms of urinary tract obstruction with hydronephrosis or renal dysfunction	Obstruction causing life-threatening consequences
Vulvovaginitis (symptoms)<br>(Use in studies evaluating topical study agents)<br>For other vulvovaginitis see Infection: Infection (any other than HIV infection)	Symptoms causing no or minimal interference with usual social & functional activities	Symptoms causing greater than minimal interference with usual social & functional activities	Symptoms causing inability to perform usual social & functional activities	Symptoms causing inability to perform basic self-care functions

Basic Self-care Functions – Adult: Activities such as bathing, dressing, toileting, transfer/movement, continence, and feeding.

Basic Self-care Functions – Young Children: Activities that are age and culturally appropriate (e.g., feeding self with culturally appropriate eating implement).

Usual Social & Functional Activities – Adult: Adaptive tasks and desirable activities, such as going to work, shopping, cooking, use of transportation, pursuing a hobby, etc.

Usual Social & Functional Activities – Young Children: Activities that are age and culturally appropriate (e.g., social interactions, play activities, learning tasks, etc.).

PARAMETER	GRADE 1 MILD	GRADE 2 MODERATE	GRADE 3 SEVERE	GRADE 4 VERY SEVERE
Vulvovaginitis (clinical exam) (Use in studies evaluating topical study agents) For other vulvovaginitis see Infection: Infection (any other than HIV infection)	Minimal vaginal abnormalities on examination OR Epithelial disruption < 25% of total surface	Moderate vaginal abnormalities on examination OR Epithelial disruption of 25 - 49% total surface	Severe vaginal abnormalities on examination OR Epithelial disruption 50 - 75% total surface	Vaginal perforation OR Epithelial disruption > 75% total surface
**OCULAR/VISUAL**				
Uveitis	Asymptomatic but detectable on exam	Symptomatic anterior uveitis OR Medical intervention indicated	Posterior or pan-uveitis OR Operative intervention indicated	Disabling visual loss in affected eye(s)
Visual changes (from baseline)	Visual changes causing no or minimal interference with usual social & functional activities	Visual changes causing greater than minimal interference with usual social & functional activities	Visual changes causing inability to perform usual social & functional activities	Disabling visual loss in affected eye(s)
**ENDOCRINE/METABOLIC**				
Abnormal fat accumulation (e.g., back of neck, breasts, abdomen)	Detectable by study participant (or by caregiver for young children and disabled adults)	Detectable on physical exam by health care provider	Disfiguring OR Obvious changes on casual visual inspection	NA
Diabetes mellitus	NA	New onset without need to initiate medication OR Modification of current medications to regain glucose control	New onset with initiation of medication indicated OR Diabetes uncontrolled despite treatment modification	Life-threatening consequences (e.g., ketoacidosis, hyperosmolar non-ketotic coma)
Gynecomastia	Detectable by study participant or caregiver (for young children and disabled adults)	Detectable on physical exam by health care provider	Disfiguring OR Obvious on casual visual inspection	NA
Hyperthyroidism	Asymptomatic	Symptomatic causing greater than minimal interference with usual social & functional activities OR Thyroid suppression therapy indicated	Symptoms causing inability to perform usual social & functional activities OR Uncontrolled despite treatment modification	Life-threatening consequences (e.g., thyroid storm)

Basic Self-care Functions – Adult: Activities such as bathing, dressing, toileting, transfer/movement, continence, and feeding.

Basic Self-care Functions – Young Children: Activities that are age and culturally appropriate (e.g., feeding self with culturally appropriate eating implement).

Usual Social & Functional Activities – Adult: Adaptive tasks and desirable activities, such as going to work, shopping, cooking, use of transportation, pursuing a hobby, etc.

Usual Social & Functional Activities – Young Children: Activities that are age and culturally appropriate (e.g., social interactions, play activities, learning tasks, etc.).

PARAMETER	GRADE 1 MILD	GRADE 2 MODERATE	GRADE 3 SEVERE	GRADE 4 VERY SEVERE
Hypothyroidism	Asymptomatic	Symptomatic causing greater than minimal interference with usual social & functional activities OR Thyroid replacement therapy indicated	Symptoms causing inability to perform usual social & functional activities OR Uncontrolled despite treatment modification	Life-threatening consequences (e.g., myxedema coma)
Lipoatrophy (e.g., fat loss from the face, extremities, buttocks)	Detectable by study participant (or by caregiver for young children and disabled adults)	Detectable on physical exam by health care provider	Disfiguring OR Obvious on casual visual inspection	NA

Basic Self-care Functions – Adult: Activities such as bathing, dressing, toileting, transfer/movement, continence, and feeding.

Basic Self-care Functions – Young Children: Activities that are age and culturally appropriate (e.g., feeding self with culturally appropriate eating implement).

Usual Social & Functional Activities – Adult: Adaptive tasks and desirable activities, such as going to work, shopping, cooking, use of transportation, pursuing a hobby, etc.

Usual Social & Functional Activities – Young Children: Activities that are age and culturally appropriate (e.g., social interactions, play activities, learning tasks, etc.).

LABORATORY				
**PARAMETER**	**GRADE 1 MILD**	**GRADE 2 MODERATE**	**GRADE 3 SEVERE**	**GRADE 4 VERY SEVERE**
**HEMATOLOGY**	***Standard International Units are listed in italics***			
Absolute CD4+ count **– Adult and Pediatric > 13 years** (HIV NEGATIVE ONLY)	300 – 400/mm^3<br>*300 – 400/µL*	200 – 299/mm^3<br>*200 – 299/µL*	100 – 199/mm^3<br>*100 – 199/µL*	< 100/mm^3<br>*< 100/µL*
Absolute lymphocyte count **– Adult and Pediatric > 13 years** (HIV NEGATIVE ONLY)	600 – 650/mm^3<br>*0.600 x 10^9 – 0.650 x 10^9/L*	500 – 599/mm^3<br>*0.500 x 10^9 – 0.599 x 10^9/L*	350 – 499/mm^3<br>*0.350 x 10^9 – 0.499 x 10^9/L*	< 350/mm^3<br>*< 0.350 x 10^9/L*
**Comment:** Values in children ≤ 13 years are not given for the two parameters above because the absolute counts are variable.				
Absolute neutrophil count (ANC)				
**Adult and Pediatric, > 7 days**	1,000 – 1,300/mm^3<br>*1.000 x 10^9 – 1.300 x 10^9/L*	750 – 999/mm^3<br>*0.750 x 10^9 – 0.999 x 10^9/L*	500 – 749/mm^3<br>*0.500 x 10^9 – 0.749 x 10^9/L*	< 500/mm^3<br>*< 0.500 x 10^9/L*
**Infant*,†, 2 – ≤ 7 days**	1,250 – 1,500/mm^3<br>*1.250 x 10^9 – 1.500 x 10^9/L*	1,000 – 1,249/mm^3<br>*1.000 x 10^9 – 1.249 x 10^9/L*	750 – 999/mm^3<br>*0.750 x 10^9 – 0.999 x 10^9/L*	< 750/mm^3<br>*< 0.750 x 10^9/L*
**Infant*,†, ≤1 day**	4,000 – 5,000/mm^3<br>*4.000 x 10^9 – 5.000 x 10^9/L*	3,000 – 3,999/mm^3<br>*3.000 x 10^9 – 3.999 x10^9/L*	1,500 – 2,999/mm^3<br>*1.500 x 10^9 – 2.999 x 10^9/L*	< 1,500/mm^3<br>*< 1.500 x 10^9/L*
**Comment:** Parameter changed from "Infant, < 1 day" to "Infant, ≤1 day"				
Fibrinogen, decreased	100 – 200 mg/dL<br>*1.00 – 2.00 g/L*<br>OR<br>0.75 – 0.99 x LLN	75 – 99 mg/dL<br>*0.75 – 0.99 g/L*<br>OR<br>0.50 – 0.74 x LLN	50 – 74 mg/dL<br>*0.50 – 0.74 g/L*<br>OR<br>0.25 – 0.49 x LLN	< 50 mg/dL<br>*< 0.50 g/L*<br>OR<br>< 0.25 x LLN<br>OR<br>Associated with gross bleeding

* Values are for term infants. Preterm infants should be assessed using local normal ranges.

† Use age and sex appropriate values (e.g., bilirubin).

LABORATORY				
**PARAMETER**	**GRADE 1 MILD**	**GRADE 2 MODERATE**	**GRADE 3 SEVERE**	**GRADE 4 POTENTIALLY LIFE-THREATENING**
Hemoglobin (Hgb)				
**Comment:** The Hgb values in mmol/L have changed because the conversion factor used to convert g/dL to mmol/L has been changed from 0.155 to 0.6206 (the most commonly used conversion factor). For grading Hgb results obtained by an analytic method with a conversion factor other than 0.6206, the result must be converted to g/dL using the appropriate conversion factor for that lab.				
**Adult and Pediatric ≥ 57 days** (HIV POSITIVE ONLY)	8.5 – 10.0 g/dL<br>*5.24 – 6.23 mmol/L*	7.5 – 8.4 g/dL<br>*4.62–5.23 mmol/L*	6.50 – 7.4 g/dL<br>*4.03–4.61 mmol/L*	< 6.5 g/dL<br>*< 4.03 mmol/L*
**Adult and Pediatric ≥ 57 days** (HIV NEGATIVE ONLY)	10.0 – 10.9 g/dL<br>*6.18 – 6.79 mmol/L*<br>OR<br>Any decrease<br>2.5 – 3.4 g/dL<br>*1.58 – 2.13 mmol/L*	9.0 – 9.9 g/dL<br>*5.55 - 6.17 mmol/L*<br>OR<br>Any decrease<br>3.5 – 4.4 g/dL<br>*2.14 – 2.78 mmol/L*	7.0 – 8.9 g/dL<br>*4.34 - 5.54 mmol/L*<br>OR<br>Any decrease<br>≥ 4.5 g/dL<br>*> 2.79 mmol/L*	< 7.0 g/dL<br>*< 4.34 mmol/L*
**Comment:** The decrease is a decrease from baseline				
**Infant[*,†], 36 – 56 days** (HIV POSITIVE OR NEGATIVE)	8.5 – 9.4 g/dL<br>*5.24 – 5.86 mmol/L*	7.0 – 8.4 g/dL<br>*4.31 – 5.23 mmol/L*	6.0 – 6.9 g/dL<br>*3.72 – 4.30 mmol/L*	< 6.00 g/dL<br>*< 3.72 mmol/L*
**Infant[*,†], 22 – 35 days** (HIV POSITIVE OR NEGATIVE)	9.5 – 10.5 g/dL<br>*5.87 - 6.54 mmol/L*	8.0 – 9.4 g/dL<br>*4.93 – 5.86 mmol/L*	7.0 – 7.9 g/dL<br>*4.34 – 4.92 mmol/L*	< 7.00 g/dL<br>*< 4.34 mmol/L*
**Infant[*,†], ≤ 21 days** (HIV POSITIVE OR NEGATIVE)	12.0 – 13.0 g/dL<br>*7.42 – 8.09 mmol/L*	10.0 – 11.9 g/dL<br>*6.18 – 7.41 mmol/L*	9.0 – 9.9 g/dL<br>*5.59- 6.17 mmol/L*	< 9.0 g/dL<br>*< 5.59 mmol/L*
**Correction:** Parameter changed from "Infant < 21 days" to "Infant ≤ 21 days"				
International Normalized Ratio of prothrombin time (INR)	1.1 – 1.5 x ULN	1.6 – 2.0 x ULN	2.1 – 3.0 x ULN	> 3.0 x ULN
Methemoglobin	5.0 – 10.0%	10.1 – 15.0%	15.1 – 20.0%	> 20.0%
Prothrombin Time (PT)	1.1 – 1.25 x ULN	1.26 – 1.50 x ULN	1.51 – 3.00 x ULN	> 3.00 x ULN
Partial Thromboplastin Time (PTT)	1.1 – 1.66 x ULN	1.67 – 2.33 x ULN	2.34 – 3.00 x ULN	> 3.00 x ULN
Platelets, decreased	100,000 – 124,999/mm^3<br>*100.000 x 10^9 – 124.999 x 10^9/L*	50,000 – 99,999/mm^3<br>*50.000 x 10^9 – 99.999 x 10^9/L*	25,000 – 49,999/mm^3<br>*25.000 x 10^9 – 49.999 x 10^9/L*	< 25,000/mm^3<br>*< 25.000 x 10^9/L*
WBC, decreased	2,000 – 2,500/mm^3<br>*2.000 x 10^9 – 2.500 x 10^9/L*	1,500 – 1,999/mm^3<br>*1.500 x 10^9 – 1.999 x 10^9/L*	1,000 – 1,499/mm^3<br>*1.000 x 10^9 – 1.499 x 10^9/L*	< 1,000/mm^3<br>*< 1.000 x 10^9/L*

[*] Values are for term infants. Preterm infants should be assessed using local normal ranges.

[†] Use age and sex appropriate values (e.g., bilirubin).

LABORATORY				
**PARAMETER**	**GRADE 1 MILD**	**GRADE 2 MODERATE**	**GRADE 3 SEVERE**	**GRADE 4 VERY SEVERE**
**CHEMISTRIES**	***Standard International Units are listed in italics***			
Acidosis	NA	pH < normal, but ≥ 7.3	pH < 7.3 without life-threatening consequences	pH < 7.3 with life-threatening consequences
Albumin, serum, low	3.0 g/dL – < LLN<br>*30 g/L – < LLN*	2.0 – 2.9 g/dL<br>*20 – 29 g/L*	< 2.0 g/dL<br>*< 20 g/L*	NA
Alkaline Phosphatase	1.25 – 2.5 x ULN†	2.6 – 5.0 x ULN†	5.1 – 10.0 x ULN†	> 10.0 x ULN†
Alkalosis	NA	pH > normal, but ≤ 7.5	pH > 7.5 without life-threatening consequences	pH > 7.5 with life-threatening consequences
ALT (SGPT)	1.25 – 2.5 x ULN	2.6 – 5.0 x ULN	5.1 – 10.0 x ULN	> 10.0 x ULN
AST (SGOT)	1.25 – 2.5 x ULN	2.6 – 5.0 x ULN	5.1 – 10.0 x ULN	> 10.0 x ULN
Bicarbonate, serum, low	16.0 mEq/L – < LLN<br>*16.0 mmol/L – < LLN*	11.0 – 15.9 mEq/L<br>*11.0 – 15.9 mmol/L*	8.0 – 10.9 mEq/L<br>*8.0 – 10.9 mmol/L*	< 8.0 mEq/L<br>*< 8.0 mmol/L*
**Comment:** Some laboratories will report this value as Bicarbonate (HCO_3) and others as Total Carbon Dioxide (CO_2). These are the same tests; values should be graded according to the ranges for Bicarbonate as listed above.				
Bilirubin (Total)				
**Adult and Pediatric > 14 days**	1.1 – 1.5 x ULN	1.6 – 2.5 x ULN	2.6 – 5.0 x ULN	> 5.0 x ULN
**Infant*†, ≤ 14 days** (non-hemolytic)	NA	20.0 – 25.0 mg/dL<br>*342 – 428 µmol/L*	25.1 – 30.0 mg/dL<br>*429 – 513 µmol/L*	> 30.0 mg/dL<br>*> 513.0 µmol/L*
**Infant*†, ≤ 14 days** (hemolytic)	NA	NA	20.0 – 25.0 mg/dL<br>*342 – 428 µmol/L*	> 25.0 mg/dL<br>*> 428 µmol/L*
Calcium, serum, high				
**Adult and Pediatric ≥ 7 days**	10.6 – 11.5 mg/dL<br>*2.65 – 2.88 mmol/L*	11.6 – 12.5 mg/dL<br>*2.89 – 3.13 mmol/L*	12.6 – 13.5 mg/dL<br>*3.14 – 3.38 mmol/L*	> 13.5 mg/dL<br>*> 3.38 mmol/L*
**Infant*†, < 7 days**	11.5 – 12.4 mg/dL<br>*2.88 – 3.10 mmol/L*	12.5 – 12.9 mg/dL<br>*3.11 – 3.23 mmol/L*	13.0 – 13.5 mg/dL<br>*3.245 – 3.38 mmol/L*	> 13.5 mg/dL<br>*> 3.38 mmol/L*
Calcium, serum, low				
**Adult and Pediatric ≥ 7 days**	7.8 – 8.4 mg/dL<br>*1.95 – 2.10 mmol/L*	7.0 – 7.7 mg/dL<br>*1.75 – 1.94 mmol/L*	6.1 – 6.9 mg/dL<br>*1.53 – 1.74 mmol/L*	< 6.1 mg/dL<br>*< 1.53 mmol/L*
**Infant*†, < 7 days**	6.5 – 7.5 mg/dL<br>*1.63 – 1.88 mmol/L*	6.0 – 6.4 mg/dL<br>*1.50 – 1.62 mmol/L*	5.50 – 5.90 mg/dL<br>*1.38 – 1.51 mmol/L*	< 5.50 mg/dL<br>*< 1.38 mmol/L*
**Comment:** Do not adjust Calcium, serum, low or Calcium, serum, high for albumin				

*Values are for term infants. Preterm infants should be assessed using local normal ranges.

† Use age and sex appropriate values (e.g., bilirubin).

LABORATORY					
PARAMETER		GRADE 1 MILD	GRADE 2 MODERATE	GRADE 3 SEVERE	GRADE 4 VERY SEVERE
Cardiac troponin I (cTnI)		NA	NA	NA	Levels consistent with myocardial infarction or unstable angina as defined by the manufacturer
Cardiac troponin T (cTnT)		NA	NA	NA	≥ 0.20 ng/mL<br>OR<br>Levels consistent with myocardial infarction or unstable angina as defined by the manufacturer
Cholesterol (fasting)					
	**Adult ≥ 18 years**	200 – 239 mg/dL<br>*5.18 – 6.19 mmol/L*	240 – 300 mg/dL<br>*6.20 – 7.77 mmol/L*	> 300 mg/dL<br>*> 7.77 mmol/L*	NA
	**Pediatric < 18 years**	170 – 199 mg/dL<br>*4.40 – 5.15 mmol/L*	200 – 300 mg/dL<br>*5.16 – 7.77 mmol/L*	> 300 mg/dL<br>*> 7.77 mmol/L*	NA
Creatine Kinase		3.0 – 5.9 x ULN†	6.0 – 9.9 x ULN†	10.0 – 19.9 x ULN†	≥ 20.0 x ULN†
Creatinine		1.1 – 1.3 x ULN†	1.4 – 1.8 x ULN†	1.9 – 3.4 x ULN†	≥ 3.5 x ULN†
Glucose, serum, high					
	Nonfasting	116 – 160 mg/dL<br>*6.44 – 8.88 mmol/L*	161 – 250 mg/dL<br>*8.89 – 13.88 mmol/L*	251 – 500 mg/dL<br>*13.89 – 27.75 mmol/L*	> 500 mg/dL<br>*> 27.75 mmol/L*
	Fasting	110 – 125 mg/dL<br>*6.11 – 6.94 mmol/L*	126 – 250 mg/dL<br>*6.95 – 13.88 mmol/L*	251 – 500 mg/dL<br>*13.89 – 27.75 mmol/L*	> 500 mg/dL<br>*> 27.75 mmol/L*
Glucose, serum, low					
	**Adult and Pediatric ≥ 1 month**	55 – 64 mg/dL<br>*3.05 – 3.55 mmol/L*	40 – 54 mg/dL<br>*2.22 – 3.06 mmol/L*	30 – 39 mg/dL<br>*1.67 – 2.23 mmol/L*	< 30 mg/dL<br>*< 1.67 mmol/L*
	**Infant*†, < 1 month**	50 – 54 mg/dL<br>*2.78 – 3.00 mmol/L*	40 – 49 mg/dL<br>*2.22 – 2.77 mmol/L*	30 – 39 mg/dL<br>*1.67 – 2.21 mmol/L*	< 30 mg/dL<br>*< 1.67 mmol/L*
Lactate		ULN - < 2.0 x ULN without acidosis	≥ 2.0 x ULN without acidosis	Increased lactate with pH < 7.3 without life-threatening consequences	Increased lactate with pH < 7.3 with life-threatening consequences
**Comment:** Added ULN to Grade 1 parameter					

* Values are for term infants. Preterm infants should be assessed using local normal ranges.

† Use age and sex appropriate values (e.g., bilirubin).

LABORATORY				
**PARAMETER**	**GRADE 1 MILD**	**GRADE 2 MODERATE**	**GRADE 3 SEVERE**	**GRADE 4 VERY SEVERE**
LDL cholesterol (fasting)				
**Adult ≥ 18 years**	130 – 159 mg/dL<br>*3.37 – 4.12 mmol/L*	160 – 190 mg/dL<br>*4.13 – 4.90 mmol/L*	≥ 190 mg/dL<br>*≥ 4.91 mmol/L*	NA
**Pediatric > 2 - < 18 years**	110 – 129 mg/dL<br>*2.85 – 3.34 mmol/L*	130 – 189 mg/dL<br>*3.35 – 4.90 mmol/L*	≥ 190 mg/dL<br>*≥ 4.91 mmol/L*	NA
Lipase	1.1 – 1.5 x ULN	1.6 – 3.0 x ULN	3.1 – 5.0 x ULN	> 5.0 x ULN
Magnesium, serum, low	1.2 – 1.4 mEq/L<br>*0.60 – 0.70 mmol/L*	0.9 – 1.1 mEq/L<br>*0.45 – 0.59 mmol/L*	0.6 – 0.8 mEq/L<br>*0.30 – 0.44 mmol/L*	< 0.60 mEq/L<br>*< 0.30 mmol/L*
Pancreatic amylase	1.1 – 1.5 x ULN	1.6 – 2.0 x ULN	2.1 – 5.0 x ULN	> 5.0 x ULN
Phosphate, serum, low				
**Adult and Pediatric > 14 years**	2.5 mg/dL – < LLN<br>*0.81 mmol/L – < LLN*	2.0 – 2.4 mg/dL<br>*0.65 – 0.80 mmol/L*	1.0 – 1.9 mg/dL<br>*0.32 – 0.64 mmol/L*	< 1.00 mg/dL<br>*< 0.32 mmol/L*
**Pediatric 1 year – 14 years**	3.0 – 3.5 mg/dL<br>*0.97 – 1.13 mmol/L*	2.5 – 2.9 mg/dL<br>*0.81 – 0.96 mmol/L*	1.5 – 2.4 mg/dL<br>*0.48 – 0.80 mmol/L*	< 1.50 mg/dL<br>*< 0.48 mmol/L*
**Pediatric < 1 year**	3.5 – 4.5 mg/dL<br>*1.13 – 1.45 mmol/L*	2.5 – 3.4 mg/dL<br>*0.81 – 1.12 mmol/L*	1.5 – 2.4 mg/dL<br>*0.48 – 0.80 mmol/L*	< 1.50 mg/dL<br>*< 0.48 mmol/L*
Potassium, serum, high	5.6 – 6.0 mEq/L<br>*5.6 – 6.0 mmol/L*	6.1 – 6.5 mEq/L<br>*6.1 – 6.5 mmol/L*	6.6 – 7.0 mEq/L<br>*6.6 – 7.0 mmol/L*	> 7.0 mEq/L<br>*> 7.0 mmol/L*
Potassium, serum, low	3.0 – 3.4 mEq/L<br>*3.0 – 3.4 mmol/L*	2.5 – 2.9 mEq/L<br>*2.5 – 2.9 mmol/L*	2.0 – 2.4 mEq/L<br>*2.0 – 2.4 mmol/L*	< 2.0 mEq/L<br>*< 2.0 mmol/L*
Sodium, serum, high	146 – 150 mEq/L<br>*146 – 150 mmol/L*	151 – 154 mEq/L<br>*151 – 154 mmol/L*	155 – 159 mEq/L<br>*155 – 159 mmol/L*	≥ 160 mEq/L<br>*≥ 160 mmol/L*
Sodium, serum, low	130 – 135 mEq/L<br>*130 – 135 mmol/L*	125 – 129 mEq/L<br>*125 – 129 mmol/L*	121 – 124 mEq/L<br>*121 – 124 mmol/L*	≤ 120 mEq/L<br>*≤ 120 mmol/L*
Triglycerides (fasting)	NA	500 – 750 mg/dL<br>*5.65 – 8.48 mmol/L*	751 – 1,200 mg/dL<br>*8.49 – 13.56 mmol/L*	> 1,200 mg/dL<br>*> 13.56 mmol/L*

* Values are for term infants. Preterm infants should be assessed using local normal ranges.

† Use age and sex appropriate values (e.g., bilirubin).

LABORATORY				
**PARAMETER**	**GRADE 1 MILD**	**GRADE 2 MODERATE**	**GRADE 3 SEVERE**	**GRADE 4 VERY SEVERE**
Uric acid	7.5 – 10.0 mg/dL<br>*0.45 – 0.59 mmol/L*	10.1 – 12.0 mg/dL<br>*0.60 – 0.71 mmol/L*	12.1 – 15.0 mg/dL<br>*0.72 – 0.89 mmol/L*	> 15.0 mg/dL<br>*> 0.89 mmol/L*
**URINALYSIS**	***Standard International Units are listed in italics***			
Hematuria (microscopic)	6 – 10 RBC/HPF	> 10 RBC/HPF	Gross, with or without clots OR with RBC casts	Transfusion indicated
Proteinuria, random collection	1 +	2 – 3 +	4 +	NA
Proteinuria, 24 hour collection				
**Adult and Pediatric ≥ 10 years**	200 – 999 mg/24 h<br>*0.200 – 0.999 g/d*	1,000 – 1,999 mg/24 h<br>*1.000 – 1.999 g/d*	2,000 – 3,500 mg/24 h<br>*2.000 – 3.500 g/d*	> 3,500 mg/24 h<br>*> 3.500 g/d*
**Pediatric > 3 mo - < 10 years**	201 – 499 mg/m^2/24 h<br>*0.201 – 0.499 g/d*	500 – 799 mg/m^2/24 h<br>*0.500 – 0.799 g/d*	800 – 1,000 mg/m^2/24 h<br>*0.800 – 1.000 g/d*	> 1,000 mg/ m^2/24 h<br>*> 1.000 g/d*

* Values are for term infants. Preterm infants should be assessed using local normal ranges.

† Use age and sex appropriate values (e.g., bilirubin).

[1] UNAIDS. Report on the global HIV/AIDS epidemic year 2009. 2009, www.unaids.org.

[2] Excler JL, Kochhar S, Kapoor S, Das S, Bahri J et al. Preparedness for AIDS vaccine trials in India. *Indian J Med Res* 2008;127:531-8.

[3] Hemelaar J, Gouws E, Ghys PD, Osmanov S. Global and regional distribution of HIV-1 genetic subtypes and recombinants in 2004. *AIDS* 2006;20:W13-23.

[4] McBurney SP, Ross TM. Viral diversity: challenges for AIDS vaccine designs. *Expert Rev Vaccines* 2008;7:1405-17.

[5] Taylor BS, Hammer SM. The challenge of HIV-1 subtype diversity. *N Engl J Med* 2008;359:1965-6.

[6] Butler IF, Pandrea I, Marx PA, Apetrei C. HIV genetic diversity: biological and public health consequences. *Curr HIV Res* 2007; 5:23-45.

[7] Lihana RW, Khamadi SA, Lwembe RM, Ochieng W, Kinyua JG, *et al*. The changing trend of HIV type 1 subtypes in Nairobi. *AIDS Res Hum Retroviruses* 2009; 25:337-42

[8] Papathanasopoulos MA, Hunt GM, Tiemessen CT. Evolution and diversity of HIV-1 in Africa - A review. *Virus Genes* 2003;26:151-63.

[9] Harris ME, Serwadda D, Sewankambo N, Kim B, Kigozi G, *et al*. Among 46 near full length HIV type 1 genome sequences from Rakai District, Uganda, subtype D and AD recombinants predominate. *AIDS Res Hum Retroviruses* 2002;18:1281-90

[10] Yirrell DL, Kaleebu P, Morgan D, Watera C, Magambo B, *et al*. Inter- and intra-genic intersubtype HIV-1 recombination in rural and semi-urban Uganda. *AIDS* 2002;16:279-86.

[11] Herbeck JT, Lyagoba F, Moore SW, Shindo N, Biryahwaho B, *et al*. Prevalence and genetic diversity of HIV type 1 subtypes A and D in women attending antenatal clinics in Uganda. *AIDS Res Hum Retroviruses* 2007;23:755-60.

[12] Yirrell DL, Kaleebu P, Morgan D, Hutchinson S, Whitworth JA. HIV-1 subtype dynamics over 10 years in a rural Ugandan cohort. *Int J STD AIDS* 2004;15:103-6.

[13] Trask SA, Derdeyn CA, Fideli U, Chen Y, Meleth S, *et al*. Molecular epidemiology of human immunodeficiency virus type 1 transmission in a heterosexual cohort of discordant couples in Zambia. *J Virol* 2002;76:397-405.

[14] Gray CM, Williamson C, Bredell H, Puren A, Xia X, *et al*. Viral dynamics and CD4+ T cell counts in subtype C human immunodeficiency virus type 1-infected individuals from southern Africa. *AIDS Res Hum Retroviruses* 2005;21:285-91.

[15] Walker BD, Burton DR. Towards an AIDS vaccine. Science 2008;320:760-4.

[16] Barouch DH. Challenges in the development of an HIV-1 vaccine. Nature 2008;455:613-9.

[17] Sodora DL, Allan JS, Apetrei C, Brenchley JM, Douek DC, Else JG, *et al.* Toward an AIDS vaccine: lessons from natural simian immunodeficiency virus infections of African nonhuman primate hosts. *Nat Med* 2009;15:861-5.

[18] Johnston MI, Fauci AS. An HIV vaccine – Challenges and Prospects. *N Engl J Med* 2008;359:888-90.

[19] Kaufman DR, Barouch DH. Translational minireview series on vaccines for HIV: T lymphocyte trafficking and vaccine-elicited mucosal immunity. *Clinical and Experimental Immunology 2009;* 157: 165–173.

[20] Franchini G. Choosing the right memory T cell for HIV. *Nature Med* 2009;15:244-6.

[21] Masopust D. Developing an HIV cytotoxic T-lymphocyte vaccine: issues of CD8 T-cell quantity, quality and location. *J Intern Med* 2008;265:125–137.

[22] Coutsinos Z, Absi Z, Henin Y, Guillet JG, Launay O. Designing an effective AIDS vaccine: strategies and current status. *Rev Med Interne* 2008;29:632-41.

[23] Mascola, J. R., G. Stiegler, T. C. VanCott, H. Katinger, C. B. Carpenter, C. E. Hanson, H. Beary, D. Hayes, S. S. Frankel, D. L. Birx, and M. G. Lewis. Protection of macaques against vaginal transmission of a pathogenic HIV-1/SIV chimeric virus by passive infusion of neutralizing antibodies. *Nat Med* 2000;6:207-10.

[24] Pantophlet R, Burton DR. GP120: target for neutralizing HIV-1 antibodies. *Annu Rev Immunol* 2006;24:739-69.

[25] Hessell AJ, Poignard P, Hunter M, Hangartner L, Tehrani DM, Bleeker WK, *et al.* Effective, low-titer antibody protection against low-dose repeated mucosal SHIV challenge in macaques. *Nat Med* 2009;15:951-4.

[26] Zhang MY, Dimitrov DS. Novel approaches for identification of broadly cross-reactive HIV-1 neutralizing human monoclonal antibodies and improvement of their potency. *Curr Pharm Des* 2007;13:203-12.

[27] Montero M, van Houten NE, Wang X, Scott JK. The membrane-proximal external region of the human immunodeficiency virus type 1 envelope: dominant site of antibody neutralization and target for vaccine design. *Microbiol Mol Biol Rev* 2008;72:54-84.

[28] Burton DR, Desrosiers RC, Johnson PR, Koff WC. An AIDS vaccine: no time to give up. *Lancet* 2004;364:1938.

[29] Montefiori D, Sattentau Q, Flores J, Esparza J, Mascola J; Working Group convened by the Global HIV Vaccine Enterprise. Antibody-based HIV-1 vaccines: recent developments and future directions. *PLoS Med* 2007;4:e348.

[30] Borrow P, Lewicki H, Hahn BH, Shaw GM, Oldstone MB. Virus-specific CD8+ cytotoxic T lymphocyte activity associated with control of viremia in primary human immunodeficiency virus type 1 infection. *J Virol 1994;* 68: 6103–10.

[31] Koup RA, Safrit JT, Cao Y, Andrews CA, McLeod G, *et al.* Temporal association of cellular immune responses with the initial control of viremia in primary human immunodeficiency virus type 1 syndrome. *J Virol* 1994; 68: 4650–5.

[32] Burgers WA, Riou C, Mlotshwa M, Maenetje P, de Assis Rosa D*, et al*. Association of HIV-specific and total CD8+ T memory phenotypes in subtype C HIV-1 infection with viral set point. *J Immunol* 2009; 182:4751-61.

[33] Jin X, Bauer DE, Tuttleton SE, Lewin S, Gettie A, *et al.* Dramatic rise in plasma viremia after CD8+ T cell depletion in simian immunodeficiency virus-infected macaques. *J Exp Med* 1999; 189: 991–8.

[34] Friedrich TC, Valentine LE, Yant LJ, Rakasz EG, Piaskowski SM, *et al*. Subdominant CD8+ T cell responses are involved in durable control of AIDS virus replication. *J Virol* 2007*;* 81: 3465–76.

[35]Watkins DI, Burton DR, Kallas EG, Moore JP, Koff WC. Nonhuman primate models and the failure of the Merck HIV-1 vaccine in humans. *Nat Med* 2008;14:617-21.

[36] Wilson NA, Keele BF, Reed JS, Piaskowski SM, Mac Nair CE, *et al*. Vaccine-induced Cellular Responses Control SIV Replication After Heterologous Challenge*. J Virol* 2009; 83:6508-21.

[37] Masopust D. Developing an HIV cytotoxic T-lymphocyte vaccine: issues of CD8 T-cell quantity, quality and location. *J Intern Med* 2008; 265: 125–37.

[38] Sallusto F, Geginat J, Lanzavecchia A. Central memory and effector memory T cell subsets: function, generation, and maintenance. *Annu Rev Immunol* 2004; 22:745-63.

[39] Pahar B, Lackner AA, Piatak M Jr, Lifson JD, Wang X, *et al*. Control of viremia and maintenance of intestinal CD4(+) memory T cells in SHIV(162P3) infected macaques after pathogenic SIV(MAC251) challenge. *Virology* 2009; 387:273-84.

[40] Hess C, Altfeld M, Thomas SY, Addo MM, Rosenberg ES, *et al*. HIV-1 specific CD8+ T cells with an effector phenotype and control of viral replication. *Lancet* 2004; 363:863-6.

[41] Rosenberg ES, Walker BD. HIV type 1-specific helper T cells: a critical host defense. *AIDS Res Hum Retroviruses* 1998; 14(Suppl 2):S143-S147.

[42] Dyer WB, Zaunders JJ, Yuan FF, Wang B, Learmont JC, *et al*. Mechanisms of HIV non-progression; robust and sustained CD4+ T-cell proliferative responses to p24 antigen correlate with control of viremia and lack of disease progression after long-term transfusion-acquired HIV-1 infection. *Retrovirology* 2008; 5:112.

[43] Sacha JB, Giraldo-Vela JP, Buechler MB, Martins MA, Maness NJ, *et al*. Gag- and Nef-specific CD4+ T cells recognize and inhibit SIV replication in infected macrophages early after infection. *Proc Natl Acad Sci USA* 2009; 106:9791-6.

[44] Riddell SR, Greenberg PD. T-Cell therapy of cytomegalovirus and human immunodeficiency virus infection. *Journal of Antimicrobial Chemotherapy*; 2000; 45: 35-45

[45] Lichterfeld M, Kaufmann DE, Yu XG, Mui SK, Addo MM, *et al*. Loss of HIV-1-specific CD8+ T cell proliferation after acute HIV-1 infection and restoration by vaccine-induced HIV-1-specific CD4+ T cells. *J Exp Med* 2004; 200:701-12.

[46] Woodland DL. Jump-starting the immune system: prime-boosting comes of age. *Trends Immuno* 2004; 25:98-104.

[47] Radosević K, Rodriguez A, Lemckert A, Goudsmit J. Heterologous prime-boost vaccinations for poverty-related diseases: advantages and future prospects. *Expert Rev Vaccines* 2009; 8:577-92.

[48] Xu J, Ren L, Huang X, Qiu C, Liu Y, *et al.* Sequential priming and boosting with heterologous HIV immunogens predominantly stimulated T cell immunity against conserved epitopes. *AIDS* 2006;20:2293-303.

[49] Liu J, O'Brien KL, Lynch DM, Simmons NL, La Porte A, *et al*. Immune control of an SIV challenge by a T-cell-based vaccine in rhesus monkeys. *Nature* 2009;457:87-91.

[50] Stewart VA, McGrath SM, Dubois PM, Pau MG, Mettens P, *et al*. Priming with an adenovirus 35-circumsporozoite protein (CS) vaccine followed by RTS,S/AS01B boosting significantly improves immunogenicity to Plasmodium falciparum CS compared to that with either malaria vaccine alone. *Infect Immun* 2007;75:2283-90.

[51] Garçon N, Chomez P, Van Mechelen M. GlaxoSmithKline Adjuvant Systems in vaccines: concepts, achievements and perspectives. *Expert Rev Vaccines* 2007;6:723-39.

[52] Vandepapelière P, Horsmans Y, Moris P, Van Mechelen M, Janssens M, *et al*. Vaccine adjuvant systems containing monophosphoryl lipid A and QS21 induce strong and persistent humoral and T cell responses against hepatitis B surface antigen in healthy adult volunteers. *Vaccine* 2008;4:1375-86.

[53] Bejon P, Lusingu J, Olotu A, Leach A, Lievens M, *et al*. Efficacy of RTS,S/AS01E vaccine against malaria in children 5 to 17 months of age. *N Engl J Med* 2008;359:2521-32.

[54] Van Braeckel E, Bourguignon P, Koutsoukos M, Clement F, Janssens M, *et al.* Induction of a potent polyfunctional CD4 + T cell response by an adjuvanted protein HIV vaccine candidate in seronegative volunteers. AIDS Vaccine 2008, 13-16 Otctober 2008, Cape Town, South Africa, [Abstract OA05-02]. AIDS Res Human Restroviruses 2008; 24: Supp 1.

[55] Sivan AV, Lee T, Binn LN, Gaydos JC. Adenovirus-associated acute respiratory disease in healthy adolescents and adults: a literature review. *Mil Med* 2007;172: 1198-203.

[56] Vogels R, Zuijdgeest D, van Meerendonk M, Companjen A, Gillissen G, *et al*. High-level expression from two independent expression cassettes in replication-incompetent adenovirus type 35 vector. *J Gen Virol* 2007;88:2915-24

[57] Barouch DH, Nabel GJ. Adenovirus vector-based vaccines for human immunodeficiency virus type 1. *Hum Gene Ther* 2005;16:149-56.

[58] Letvin NL, Huang Y, Chakrabarti BK, Xu L, Seaman MS, *et al*. Heterologous envelope immunogens contribute to AIDS vaccine protection in rhesus monkeys. *J Virol* 2004;78:7490-7.

[59] Santra S, Seaman MS, Xu L, Barouch DH, Lord CI, *et al*. Replication-defective adenovirus serotype 5 vectors elicit durable cellular and humoral immune responses in nonhuman primates. *J Virol* 2005;79:6516-22.

[60] Shiver JW, Emini EA. Recent advances in the development of HIV-1 vaccines using replication-incompetent adenovirus vectors. *Annu Rev Med* 2004;55:355-72.

[61] Shiver JW, Fu TM, Chen L, Casimiro DR, Davies ME, *et al*. Replication-incompetent adenoviral vaccine vector elicits effective anti-immunodeficiency-virus immunity. *Nature* 2002;415:331-5.

[62] Graham BS, Koup RA, Roederer M, Bailer RT, Enama ME, *et al*. Phase 1 safety and immunogenicity evaluation of a multiclade HIV-1 DNA candidate vaccine. *J Infect Dis* 2006;194:1650-60.

[63] Priddy FH, Brown D, Kublin J, Monahan K, Wright DP, Lalezari J, *et al*. Safety and immunogenicity of a replication-incompetent adenovirus type 5 HIV-1 clade B gag/pol/nef vaccine in healthy adults. *Clin Infect Dis* 2008;46:1769-81.

[64] Patterson LJ, Robert-Guroff M. Replicating adenovirus vector prime/protein boost strategies for HIV vaccine development. *Expert Opin Biol Ther* 2008;8:1347-63.

[65] Catanzaro AT, Koup RA, Roederer M, Bailer RT, Enama ME, *et al.* Phase 1 safety and immunogenicity evaluation of a multiclade HIV-1 candidate vaccine delivered by a replication defective recombinant adenovirus vector. *J Infect Dis* 2006;194:1638-49.

[66] Klein MR, van Baalen CA, Holwerda AM, Kerkhof Garde SR, Bende RJ, *et al*. Kinetics of Gagspecific cytotoxic T lymphocyte responses during the clinical course of HIV-1 infection: a longitudinal analysis of rapid progressors and long-term asymptomatics. *J Exp Med* 1995;181:1365-72.

[67] Musey L, Hughes J, Schacker T, Shea T, Corey L, *et al*. Cytotoxic-T-cell responses, viral load, and disease progression in early human immunodeficiency virus type 1 infection. *N Engl J Med* 1997; 337: 1267-74.

[68] Schmitz JE, Kuroda MJ, Santra S, Sasseville VG, Simon MA, *et al*. Control of viremia in simian immunodeficiency virus infection by CD8+ lymphocytes. *Science* 1999;283:857-60.

[69] Yang O, Kalams SA, Rosenzweig M, Trocha A, Jones N, *et al*. Efficient lysis of human immunodeficiency virus type 1-infected cells by cytotoxic T lymphocytes. *J Virol* 1996;70:5799-806.
