# Supplementary material for: A Phase I Double Blind, Placebo-Controlled, Randomized Study of the Safety and Immunogenicity of an Adjuvanted HIV-1 Gag-Pol-Nef Fusion Protein and Adenovirus 35 Gag-RT-Int-Nef Vaccine in Healthy HIV-Uninfected African Adults
Source: PLoS One. 2015 May 11;10(5):e0125954. doi: 10.1371/journal.pone.0125954 (PMC4427332; doi:10.1371/journal.pone.0125954)
Supplement: S1 Table — (DOCX) [file pone.0125954.s005.docx]

**Table S1. IFN-γ ELISpot responders. Number of responders/total (% responders)**

| **Group** |  | **M0** | **M1** | **M2** | **M3** | **M4** | **M5** | **M16** |
| --- | --- | --- | --- | --- | --- | --- | --- | --- |
| **A** | **Any F4** | 0/27 (0) |  | 2/28 (7) |  | 1/31 (3) | 3/30 (10) | 1/29 (3) |
| **B** |  | 0/28 (0) |  | 8/25 (32) |  | 3/27 (11) | 6/26 (23.1) | 6/26 (23) |
| **C** |  | 0/28 (0) | 5/26 (19) |  | 7/24 (29) |  | 17/21 (81) | 13/25 (52) |
| **D** |  | 0/28 (0) | 6/24 (25) | 21/24 (88) |  | 15/22 (68) | 19/24 (79) | 14/21 (67) |
| **Placebo** |  | 0/25 (0) | 0/12 (0) | 0/21 (0) | 0/6 (0) | 0/19 (0) | 1/28 (4) | 0/27 (0) |
| **A** | **Any GRIN** | 0/27 (0) |  | 1/28 (4) |  | 0/31 (0) | 15/30 (50) | 7/29 (24) |
| **B** |  | 0/28 (0) |  | 5/25 (20) |  | 2/27 (7) | 14/26 (54) | 9/26 (35) |
| **C** |  | 0/28 (0) | 19/26 (73) |  | 18/24 (75) |  | 21/21 (100) | 20/25 (80) |
| **D** |  | 0/28 (0) | 20/24 (83) | 22/24 (92) |  | 19/22 (86) | 22/24 (92) | 18/21 (86) |
| **Placebo** |  | 0/25 (0) | 0/12 (0) | 0/21 (0) | 0/6 (0) | 0/19 (0) | 1/28 (4) | 0/27 (0) |
