# Supplementary material for: A Phase I Double Blind, Placebo-Controlled, Randomized Study of the Safety and Immunogenicity of an Adjuvanted HIV-1 Gag-Pol-Nef Fusion Protein and Adenovirus 35 Gag-RT-Int-Nef Vaccine in Healthy HIV-Uninfected African Adults
Source: PLoS One. 2015 May 11;10(5):e0125954. doi: 10.1371/journal.pone.0125954 (PMC4427332; doi:10.1371/journal.pone.0125954)
Supplement: S2 Table — (DOCX) [file pone.0125954.s006.docx]

**Table S2. F4- and GRIN-specific CD40L+ CD4+ / CD8+ T cell responders. Number of responders/total (% responders)**

| **Group** |  | **M0** | **M1** | **M1.5** | **M2** | **M3** | **M4** | **M5** | **M16** |
| --- | --- | --- | --- | --- | --- | --- | --- | --- | --- |
| **A** | **F4**  **CD4** | 0/29 (0) |  | 23/23 (100) | 25/26 (96) |  | 27/28 (96) | 27/27 (100) | 27/27 (100) |
| **B** |  | 0/28 (0) |  | 18/18 (100) | 23/24 (96) |  | 23/26 (89) | 24/26 (92) | 24/25 (96) |
| **C** |  | 0/27 (0) | 20/26 (77) |  |  | 10/26 (39) |  | 24/24 (100) | 23/26 (89) |
| **D** |  | 0/27 (0) | 20/22 (91) |  | 24/24 (100) |  | 21/21 (100) | 19/19 (100) | 21/21 (100) |
| **Placebo** |  | 2/29 (7) | 0/14 (0) | 1/8 (13) | 0/22 (0) | 0/6 (0) | 0/20 (0) | 1/28 (4) | 0/24 (0) |
| **A** | **GRIN**  **CD4** | 1/28 (4) |  | 18/22 (82) | 19/25 (76) |  | 20/28 (71) | 20/26 (77) | 17/26 (65) |
| **B** |  | 0/28 (0) |  | 13/18 (72) | 17/24 (71) |  | 19/26 (73) | 22/26 (85) | 21/25 (84) |
| **C** |  | 2/27 (7) | 20/26 (77) |  |  | 12/25 (48) |  | 22/24 (92) | 20/26 (77) |
| **D** |  | 1/27 (4) | 17/22 (77) |  | 23/23 (100) |  | 19/20 (95) | 19/19 (100) | 20/22 (91) |
| **Placebo** |  | 1/28 (4) | 0/12 (0) | 0/8 (0) | 1/20 (5) | 0/6 (0) | 1/19 (5) | 1/27 (4) | 2/23 (9) |
| **A** | **F4**  **CD8** | 1/29 (3) |  | 1/23 (4) | 0/26 (0) |  | 2/28 () | 7/27 (26) | 4/27 (15) |
| **B** |  | 1/28 (4) |  | 2/18 (11) | 2/24 (8) |  | 0/26 (0) | 8/26 (31) | 2/25 (8) |
| **C** |  | 2/27 (7) | 10/26 (39) |  |  | 7/26 (27) |  | 5/24 (21) | 4/26 (15) |
| **D** |  | 1/27 (4) | 9/22 (41) |  | 9/24 (38) |  | 8/21 (38) | 9/19 (47) | 10/21 (48) |
| **Placebo** |  | 1/29 (3) | 1/14 (7) | 0/8 (0) | 0/22 (0) | 1/6 (17) | 0/20 (0) | 0/28 (0) | 1/24 (4) |
| **A** | **GRIN**  **CD8** | 0/28 (0) |  | 0/22 (0) | 0/25 (0) |  | 0/28 (0) | 11/26 (42) | 9/26 (35) |
| **B** |  | 1/28 (4) |  | 1/18 (6) | 0/24 (0) |  | 0/26 (0) | 11/26 (42) | 9/25 (36) |
| **C** |  | 4/27 (15) | 23/26 (89) |  |  | 16/25 (64) |  | 10/24 (42) | 9/26 (35) |
| **D** |  | 1/27 (4) | 17/22 (77) |  | 20/23 (87) |  | 16/20 (80) | 16/19 (84) | 18/22 (82) |
| **Placebo** |  | 0/28 (0) | 1/12 (8) | 0/8 (0) | 0/20 (0) | 0/6 (0) | 0/19 (0) | 0/27 (0) | 1/23 (4) |
