# Supplementary material for: A Phase I Double Blind, Placebo-Controlled, Randomized Study of the Safety and Immunogenicity of an Adjuvanted HIV-1 Gag-Pol-Nef Fusion Protein and Adenovirus 35 Gag-RT-Int-Nef Vaccine in Healthy HIV-Uninfected African Adults
Source: PLoS One. 2015 May 11;10(5):e0125954. doi: 10.1371/journal.pone.0125954 (PMC4427332; doi:10.1371/journal.pone.0125954)
Supplement: S1 Text — Additional safety data. (DOCX) [file pone.0125954.s007.docx]

**Supplementary Safety *Results***

Local reactogenicity: Three volunteers experienced severe local reactions in Group B, one in Group C, and one in Group D. In all groups, there was a trend for lower frequency of local reactogenicity after the third administration.

Systemic reactogenicity: Nineteen volunteers had a severe (grade 3) reaction. Three volunteers in Group A, with lower dose adjuvant, and 5 in Group B, with higher dose adjuvant, had severe systemic reactions. Seven of these were after the first or second F4co/AS01 dose. Five of these (1 from Group A) had a combination of chills, malaise, myalgia, arthralgia, headache and fatigue beginning within 24 hours of the first, second or third vaccination and resolving within 2 days. One of these from Group A also had a fever of 39.5ºC on the day of first vaccination lasting 1 day and one from Group B also had a fever of 39.5ºC eight days after first vaccination lasting 1 day. The second volunteer from Group A had severe abdominal pain and moderate chills three days after first vaccination, resolving within 2 days. The third volunteer from Group A had severe headache and arthralgia within 24 hours after first vaccination together with moderate chills, myalgia, malaise, nausea, abdominal pain and diarrhea. The headache, arthralgia, chills and myalgia resolved within 5 days, but malaise, nausea, abdominal pain and diarrhea persisted to 13 days. This volunteer had a concurrent adverse event of prolonged menses, and three months later was diagnosed with urinary schistosomiasis. The fifth volunteer from Group B had severe headache, arthralgia, malaise, fatigue, myalgia and chills within 24 hours after first vaccination. Three vaccine recipients in Group C had severe systemic reactions. Two were after the second F4co/AS01B dose; one was after both the Ad35-GRIN prime and the second F4co/AS01B boost. One had severe chills, malaise, myalgias, arthralgias, headache, fatigue and moderate subjective fever within 24 hours after third vaccination which resolved within 5 days. One had severe fatigue within 24 hours of the first vaccination, resolving in 3 days, and severe chills within 24 hours after the third vaccination with moderate malaise and fatigue, all resolving in 3 days. One volunteer experienced severe myalgia 13 days after the third vaccination. Six vaccine recipients in Group D had severe systemic reactions. Two were after the first co-administration; three were after the second co-administration; one was after both the second and third co-administration. Five of these had a combination of chills, headache, malaise, fatigue, myalgia, arthralgia and abdominal pain typically beginning within 24 hours of the first, second or third vaccination and resolving in 1-2 days. One of these 5 had a severe fever of 39.5ºC for one day in addition to some of the above symptoms (all moderate) after the second vaccination. The sixth volunteer had severe arthralgia and moderate chills, myalgia, headache and fatigue within 24 hours after first vaccination but these symptoms were attributed to adverse events of herpes labialis and malaria (diagnostic blood smear), diagnosed on follow-up at Day +1 and rated as not related to vaccination. One placebo volunteer had severe fatigue six days after receiving the saline placebo injection, with moderate chills, arthralgia and headache from 0 to 7 days after vaccination but these symptoms were attributed to an upper respiratory infection, diagnosed clinically at Day +5. Another placebo volunteer had severe diarrhea seven days after vaccination, with moderate malaise, abdominal pain and subjective fever. All reactogenicity events were transient and resolved spontaneously.

Adverse events: Of 394 clinical adverse events, 7 were judged as probably or possibly related to vaccine - moderate (Grade 2) pain in the arm of vaccination 16 days after first injection and moderate (Grade 2) papular rash which were self-limited, and 5 mild (Grade 1) lens opacities (4 in vaccine recipients and 1 in a placebo recipient). There was no significant difference in the proportion of lens opacities between vaccine (3%) and placebo (3%) recipients (Fisher’s exact test; p=1.00). The percentage of volunteers with moderate or above non-serious adverse events within 28 days post-vaccination was 23% in Group A, 31% in Group B, 39% in Group C, 38% in Group D and 38% among the placebo recipients (p=0.61).

Three pregnancies occurred in vaccine recipients during the first 36 weeks of the study when contraception was required. Two resulted in the delivery of healthy full-term newborns. One resulted in an uncomplicated spontaneous abortion (SAB) at 27 weeks gestation. An additional 5 pregnancies occurred after week 36, 1 of which was in a placebo recipient. Two are continuing uncomplicated pregnancies, one resulted in delivery of healthy full-term newborn; one resulted in an uncomplicated SAB at 10 weeks gestation, one resulted in a SAB complicated by malaria at 12 weeks.

Of the 66 volunteers scheduled for ophthalmologic examination, 65 completed the post-trial examination. One volunteer left the country and was not available for the post-trial examination. Volunteers with AREDS scores ≥1 at baseline assessment were not eligible. (Of the 128 potential volunteers screened for enrollment, 3 were excluded due to abnormalities on eye examination; one of these was cataract.) At the post trial assessment, 5 of the 65 volunteers examined had cataracts identified with lens opacity of 1.0-1.9 on the AREDS scale and the other 60 volunteers had a reading of <1. Amongst the five cases, four of the volunteers received vaccine and one received placebo. Among the four vaccinees, three were in Group A and received two doses of F4co/AS01E and one dose of Ad35-GRIN, and one was in Group B and received two doses of F4co/AS01B and one dose of Ad35-GRIN. None of the five volunteers with lens opacity reported visual symptoms at the time of diagnosis. Visual acuity was unchanged from baseline in four of the volunteers; in one volunteer visual acuity was decreased in the affected eye from 20/15 to 20/20. Due to these findings, volunteers at the other two trial centers which did not perform baseline eye exams underwent post-trial eye exams by qualified ophthalmologists. Of the 79 volunteers enrolled at these centers, 72 were examined, and one vaccine recipient in Group C was diagnosed with a mild cataract. The volunteer was asymptomatic with no change in visual acuity. All volunteers have been screened for diabetes, and environmental and medication exposures, eye injuries and family history; no obvious cause for lens opacity has been identified. On follow-up, no progression has been observed. In one case, the cataracts were not detected on follow-up exam. In a second opinion by a qualified ophthalmologist, using slit-lamp digital photographs of the findings at the time of diagnosis, two of the four cases were interpreted as normal.

All volunteers will receive long-term ophthalmologic follow-up. While larger or progressive cataracts could result in disability, the cataracts observed were very small, Grade 1 on the AREDS scale, not considered clinically significant and no progression has been observed. The trial sponsor classified these events as probably not related to vaccine, since they were observed in both placebo and vaccine recipients and since second opinion and follow-up examinations suggest limitations in the use of AREDS grading scale for very small cataracts.
